# Site-specific incorporation of 5′-methyl DNA enhances the therapeutic profile of gapmer ASOs

**DOI:** 10.1093/nar/gkab047

**Published:** 2021-02-05

**Authors:** Guillermo Vasquez, Graeme C Freestone, W Brad Wan, Audrey Low, Cheryl Li De Hoyos, Jinghua Yu, Thazha P Prakash, Michael E Ǿstergaard, Xue-hai Liang, Stanley T Crooke, Eric E Swayze, Michael T Migawa, Punit P Seth

**Affiliations:** Ionis Pharmaceuticals Inc., 2855 Gazelle Court, Carlsbad, CA 92010, USA; Ionis Pharmaceuticals Inc., 2855 Gazelle Court, Carlsbad, CA 92010, USA; Ionis Pharmaceuticals Inc., 2855 Gazelle Court, Carlsbad, CA 92010, USA; Ionis Pharmaceuticals Inc., 2855 Gazelle Court, Carlsbad, CA 92010, USA; Ionis Pharmaceuticals Inc., 2855 Gazelle Court, Carlsbad, CA 92010, USA; Ionis Pharmaceuticals Inc., 2855 Gazelle Court, Carlsbad, CA 92010, USA; Ionis Pharmaceuticals Inc., 2855 Gazelle Court, Carlsbad, CA 92010, USA; Ionis Pharmaceuticals Inc., 2855 Gazelle Court, Carlsbad, CA 92010, USA; Ionis Pharmaceuticals Inc., 2855 Gazelle Court, Carlsbad, CA 92010, USA; Ionis Pharmaceuticals Inc., 2855 Gazelle Court, Carlsbad, CA 92010, USA; Ionis Pharmaceuticals Inc., 2855 Gazelle Court, Carlsbad, CA 92010, USA; Ionis Pharmaceuticals Inc., 2855 Gazelle Court, Carlsbad, CA 92010, USA; Ionis Pharmaceuticals Inc., 2855 Gazelle Court, Carlsbad, CA 92010, USA

## Abstract

We recently showed that site-specific incorporation of 2′-modifications or neutral linkages in the oligo-deoxynucleotide gap region of toxic phosphorothioate (PS) gapmer ASOs can enhance therapeutic index and safety. In this manuscript, we determined if introducing substitution at the 5′-position of deoxynucleotide monomers in the gap can also enhance therapeutic index. Introducing *R*- or *S*-configured 5′-Me DNA at positions 3 and 4 in the oligodeoxynucleotide gap enhanced the therapeutic profile of the modified ASOs suggesting a different positional preference as compared to the 2′-OMe gap modification strategy. The generality of these observations was demonstrated by evaluating *R*-5′-Me and *R*-5′-Ethyl DNA modifications in multiple ASOs targeting HDAC2, FXI and Dynamin2 mRNA in the liver. The current work adds to a growing body of evidence that small structural changes can modulate the therapeutic properties of PS ASOs and ushers a new era of chemical optimization with a focus on enhancing the therapeutic profile as opposed to nuclease stability, RNA-affinity and pharmacokinetic properties. The 5′-methyl DNA modified ASOs exhibited excellent safety and antisense activity in mice highlighting the therapeutic potential of this class of nucleic acid analogs for next generation ASO designs.

## INTRODUCTION

Chemical modifications are essential for improving the drug-like properties of nucleic acid therapeutics (1,2). Historically, chemical modifications have been used to enhance RNA-binding and nuclease stability of nucleic acid therapeutics. More recently, site-specific incorporation of chemical modifications has been used to enhance the therapeutic properties of antisense oligonucleotide (ASO) and siRNA therapeutics (3,4).

**Figure 1.**
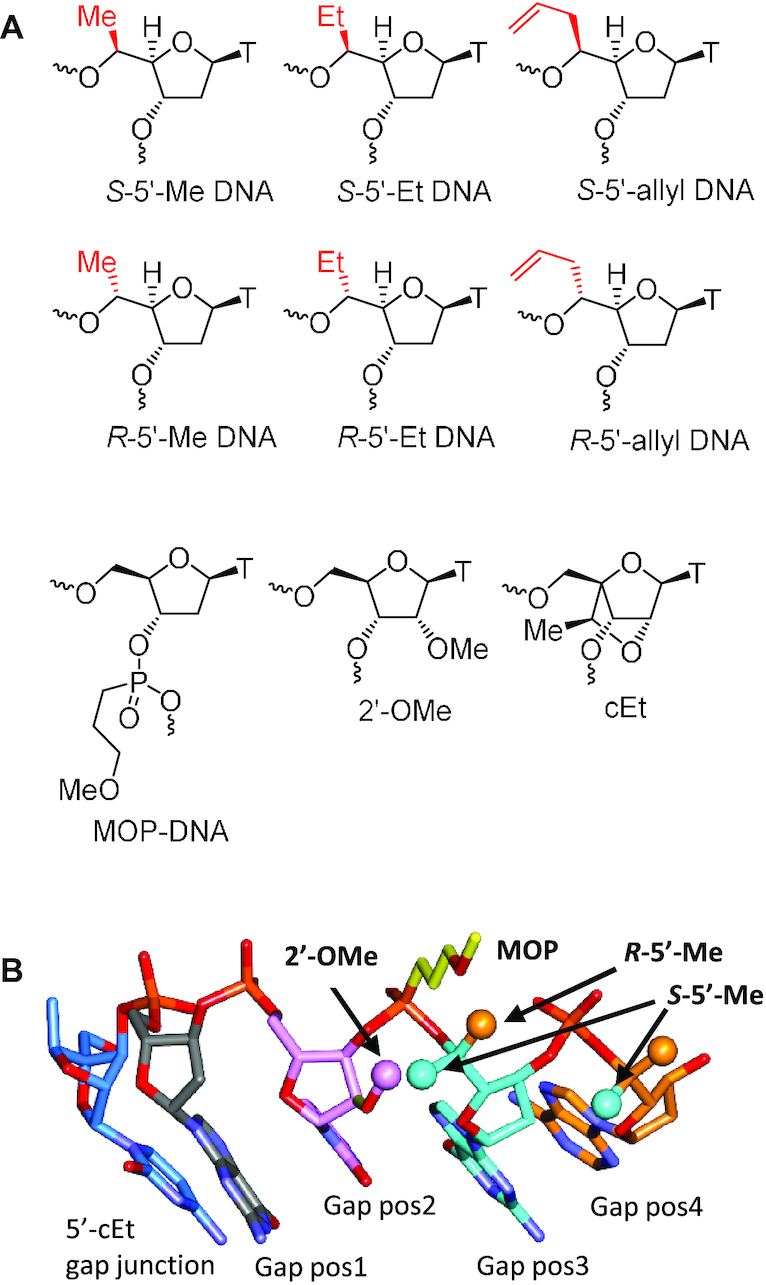
(**A**) Structures of *R-* and *S*-5′-alkyl DNA monomers evaluated in this work. (**B**) Structural model of an ASO showing the 5′-cEt-DNA gap junction. The 5′-methyl group at position 3 in the DNA gap occupies the same chemical space as the 2′-OMe group at position 2.

We recently showed that site-specific incorporation of 2′-modifications or neutral linkages in the deoxynucleotide gap region of toxic phosphorothioate (PS) gapmer antisense oligonucleotides (ASOs) can enhance therapeutic index and safety (5). We hypothesized that the 2′-or backbone modifications modulate interactions of PS-ASOs with important cellular proteins implicated in toxicity, by modulating steric bulk or conformation in the vicinity of the PS backbone. We further hypothesized that introducing substitution at the 5′-position of deoxynucleotide monomers in the gap can also create steric bulk or modulate local conformation in the vicinity of the PS backbone and improve safety (Figure 1). To test this hypothesis, we undertook the synthesis and evaluation of gapmer ASOs where deoxynucleotides in the gap were replaced with 5′-alkyl DNA nucleotides in a site-specific manner.

The family of 5′-modified nucleic acid analogs is broad and can be classified into two major structural classes. The first class includes 5′-alkyl DNA or RNA analogs that have been evaluated to improve the nuclease stability of both DNA- and RNA-based nucleic acid therapeutics (6–9). 5′-methyl DNA isomers in the gap region were shown to enhance the allele selectivity of PS ASOs targeting single nucleotide polymorphisms in a position and configuration specific manner (10). Additional variants of 5′-modified nucleic acids include 5′-methyl analogs of LNA, α-L-LNA and HNA (11–13). Changing the configuration of the 5′-alkyl substituents in these analogs had a significant impact on RNA-affinity and immunostimulatory profiles of the PS ASOs (14). The second class of 5′-modified nucleic acids include modifications such as bicyclo- and tricyclo-DNA (15–17), α,β-CNA (18,19), backbone-constricted (20,21), and dual constrained analogs of LNA (22) and α-L-LNA (23) that show significant increases in RNA-affinity and interesting biological properties (24,25). In these analogs, the 5′-substitutent is covalently tethered to restrict rotation around multiple backbone torsion angles depending on the mode of constraint employed. Additionally, 5′-alkyl analogs have also been used to modulate stability and conformation of the 5′-phosphate group to facilitate loading of siRNA into Ago2 (26).

In this manuscript, we report the synthesis and evaluation of PS ASOs modified with both stereoisomers of 5′-methyl and related 5′-alkyl DNA nucleotides. The 5′-methyl DNA nucleotides were incorporated in the deoxynucleotide gap region in a site-specific manner and the effects on potency and cytotoxicity were characterized. We found that both configurations of 5′-methyl DNA can enhance the therapeutic profile of the modified PS ASOs when incorporated at positions 3 and 4 in the deoxynucleotide gap suggesting that these modifications have a different positional preference as compared to the 2′-OMe gap modification strategy described previously (5). Furthermore, the 5′-methyl DNA ASOs can at times show improved potency relative to 2′-OMe and alkyl phosphonate gap-modified ASOs highlighting the therapeutic potential of this class of nucleic acid analogs.

## MATERIALS AND METHODS

### Oligonucleotide synthesis and purification

Oligonucleotides were synthesized on a 40 μmol scale using Nittophase UnyLinker support (317 μmol/g) on an AKTA 10 Oligopilot. Fully protected nucleoside phosphoramidites were incorporated using standard solid-phase oligonucleotide synthesis, i.e. 15% dichloroacetic acid in toluene for deblocking, 1 M 4,5-dicyanoimidazole 0.1 M *N*-methylimidazole in acetonitrile as activator for amidite couplings, 20% acetic anhydride in THF and 10% 1-methylimidazole in THF/pyridine for capping and 0.1 M xanthane hydride in pyridine:acetonitrile 1:1 (v:v) for thiolation. 3-Methoxypropylphosphonate (MOP) couplings were oxidized instead of thiolated using 20% t-BuOOH in ACN. Amidites were dissolved to 0.1 M in acetonitrile:toluene 1:1 (v:v) and incorporated using 6 min. coupling recycling time for DNA amidites and 10 min. for all other amidites. At the end of the solid phase synthesis cyanoethyl protecting groups were removed by a 30 min. treatment with 20% diethylamine in toluene. ASOs with a single MOP incorporation can be deprotected and cleaved using conc. aq. ammonia at room temperature for 48 h. For multiple MOP incorporations, the following mild deprotection conditions were used:

Protected oligonucleotide on support (400 mg) was suspended in dry THF (5 ml) and stirred for 5 minutes in a glass pressure vial. Ethylenediamine (EDA, 5 ml) was added via syringe with stirring at room temperature. The reaction was heated 55°C with stirring (oil bath) for 15 min. The reaction was cooled in an ice bath and was diluted with THF (5 ml). The reaction was centrifuged (300 rpm, 5 min), and the solvent was removed via pipette. The residue was washed with dry THF (2 × 5 ml). The pellet was suspended in 50% EtOH and the spent resin was removed by filtration. The filtrate was concentrated under reduced pressure.

Oligonucleotides were purified by ion-exchange chromatography using aqueous buffers with 100 mM NH4OAc and up to 2 M NaBr. The DMT group was removed on-column by treatment with 6% dichloroacetic acid in water. Pure fractions were desalted on a C18 reverse phase column, eluted in 50% acetonitrile in water (v:v) and lyophilized. Purity and mass of oligonucleotides was determined using ion-pair LCMS. Analytical data for the ASOs is provided in Supplementary Table S6.

### Thermal denaturation measurements

ASO and RNA were mixed in 1:1 ratio (4 μM duplex) in a buffer containing 100mM NaCl, 10 mM phosphate and 10 mM EDTA at pH 7. Oligos were hybridized with the complementary RNA strand by heating duplex to 85°C for 5 min and allowed to cool at room temperature. Thermal denaturation temperatures (Tm values) were measured in quartz cuvettes (pathlength 1.0 cm) on a Cary 100 ultraviolet (UV)/visible spectrophotometer equipped with a Peltier temperature controller. Absorbance at 260 nm was measured as a function of temperature using a temperature ramp of 0.5°C per min. *T*_m_ values were determined using the hyperchromicity method incorporated into the instrument software.

### ASO in vitro activity assay

NIH3T3 cells were mixed with PS-ASOs at the specified final concentrations in a final volume of 100 μl and added to a BTX high-throughput electroporation plate. The cells were then electroporated using the ECM 830 high-throughput electroporation system. Twenty-four hours after electroporation, total RNA was prepared using RNeasy mini Kit (Qiagene). The targeted mRNA levels were quantified through quantitation real-time PCR (qRT-PCR) assay, using TaqMan One-step qRT–PCR system with AgPath-ID One-Step RT–PCR Reagents (Thermo Fisher Scientific). Briefly, reverse transcription was performed at 45°C for 10 min and stopped by heating at 95°C for 10 min. Subsequently, 40 cycles of qPCR reactions were conducted at 95°C for 15 s and 60°C for 60 s within each cycle. qRT–PCR data were analyzed with StepOne Software v.3 (Applied Biosystems). The expression levels of target RNAs were normalized to total RNA in duplicate RNA samples quantified using Quant-iT RiboGreen RNA Reagent (Thermo Fisher Scientific). IC50 was calculated using PRISM.

### Caspase 3/7 assays

For quantitative analysis of caspase activation, Caspase-Glo 3/7 Reagent (Promega) was added directly to Hepa1-6 cells in a 96- or 384-well plate at a volume equal to the sample volume. Luminescence was recorded after 30 min using a TECAN Infinite M200 plate reader. Background readings determined from wells containing culture medium only were subtracted. Relative caspase activity was calculated as 100% × luminescence reading of a treated sample/luminescence reading of a mock-treated control.

### PS-ASO-protein binding study

Affinity selection of binding proteins using a biotinylated 5–10–5 PS-MOE gapmer PS-ASO precoated on neutravidin beads was performed essentially as described (5). Beads bound proteins were then eluted by competition with 25 ul of 25 uM ASOs of the same sequence but different design, as shown in figure legend. Eluted proteins were separated on 4–12% SDS-PAGE, following by silver staining using silver staining kit (), based on the manufactorer's protocol. Additionally, proteins in PAGE gel were transferred to membrane, and detected by western alayses. The membranes were blocked with 5% non-fat dry milk in 1 × PBS at room temperature for 30 min. Membranes were then incubated with primary antibodies at room temperature for 2 h or at 4°C overnight. After three washes with 1× PBS, the membranes were incubated with appropriate HRP-conjugated secondary antibodies (1:2000) at room temperature for 1 h to develop the image using Immobilon Forte Western HRP Substrate (Millipore). Primary antiobodies used for western analysis are: P54nrb (sc-376865, Santa Cruz Biotech.), PSF (Sc-271796, Santa Cruz Biotech.), RNase H1 (15606-1-AP, ProteinTech), Ku70 (ab3114, Abcam).

### Immunoflurescence staining and comnfocal imaging

HeLa cells grown in glass-bottom dishes were transfected for 2 h with 200 nM ASOs using Lipofactamine 2000 (Life Technologies), based on manufacturer's instruction. Cells were washed with 1× PBS, fixed with 4% paraformaldehyde for 0.5–1 h at room temperature, and permeabilized for 5 min with 0.1% Triton in 1 × PBS. After blocking at room temperature for 30 min with 1 mg/ml BSA in 1× PBS, cells were incubated with antibody (sc-376865, Santa Cruz Biotech, 1:200) against P54nrb in block buffer (1 mg/ml BSA in 1× PBS) for 2 h, washed three times (5 min each) with 0.1% nonyl phenoxypolyethoxylethanol-40 (NP-40) in 1× PBS, and incubated for 1 h with secondary antibody conjugated with AF488 (1:200). After washing three times, cells were mounted with anti-fade reagent containing DAPI (Life Technologies), and images were acquired using confocal microscope (Olympus FV-1000) and processed using FV-10 ASW 3.0 Viewer software (Olympus). Cells were counted manually for those containing misolocalized P54nrb proteins.

### RNase H1 cleavage assay

Purified human RNase H1 was diluted in buffer containing 100 mM Tris–HCl, pH 7.4, 50 mM NaCl, 30% glycerol and 10 mM DTT. ASO/RNA duplex was formed with ASO and 5′-FAM-labeled complementary RNA at a final concentration of 0.33 μM each by annealing in reaction buffer containing 20 mM Tris–HCl, pH 7.4, 50 mM NaCl, 10 mM MgCl_2_ and 10 mM DTT. RNase H1 protein was added to the duplex at a final concentration of 1 ng per reaction and the reaction was incubated at 37°C for 15 min. The reaction was stopped by adding 10 μl stop solution containing 8 M urea and 120 mm EDTA for every 20 μl of reaction mix. Samples were heated at 95°C for 5 min and separated on a 20% denaturing polyacrylamide gel, and cleavage products were visualized using a Storm PhosphorImager and analyzed with ImageQuantTL software.

### *In vivo* studies

Animal experiments were conducted according to American Association for the Accreditation of Laboratory Animal Care guidelines and were approved by the institution's Animal Welfare Committee (Cold Spring Harbor Laboratory′s Institutional Animal Care and Use Committee guidelines). Male BALB/c mice aged 6–8 weeks were obtained from Charles River Laboratories. If not otherwise specified, three animals were used per treatment. Animals were randomly grouped and all animals were included in data analysis (unless the animals were found dead before the end of the study). PS-ASOs or saline was administered subcutaneously. 72 h after injection, animals were anesthetized using 2–4% isoflurane and blood was collected by cardiac puncture or tail bleeding. Blood samples were processed to plasma and evaluated for ALT using a Beckman Coulter AU480 Bioanalyzer. Liver samples were homogenized in guanidinium isothiocyanate with 8% Beta-mercaptoethanol, and total RNA was prepared using the RNeasy (Qiagen) or the Purelink RNA purification kit (Thermo Fisher Scientific). mRNA levels were quantified through qRT-PCR using TaqMan primer probes sets with the EXPRESS One-Step Superscript™ qRT-PCR Kit. qRT–PCR data were analyzed with StepOne Software v.3 (Applied Biosystems). The expression levels of target RNAs were run in technique duplicate and normalized to total RNA quantitated by the Quant-iT RiboGreen RNA Reagent (Thermo Fisher Scientific). Average values and standard deviation were calculated from three different mice in each group. EC50 was calculated using GraphPad PRISM software.

Primer Probe sets used for qRT-PCR

**Table:** 

Mouse *FXI*	
	Forward: 5′-ACATGACAGGCGCGATCTCT-3′
	Reverse: 5′-TCTAGGTTCACGTACACATCTTTGC-3′
	Probe: 5′-TTCCTTCAAGCAATGCCCTCAGCAAT-3′
Mouse *Cxcl12*	
	Forward: 5′-CCAGAGCCAACGTCAAGCAT-3′
	Reverse: 5′-CAGCCGTGCAACAATCTGAA-3′
	Probe: 5′-TGAAAATCCTCAACACTCCAAACTGTGCC-3′
Mouse *Hdac2*	
	Forward: 5′-TGATGGTGTTGAGGAAGCTTTTT-3′
	Reverse: 5′-TCCCTCAAGTCTCCTGTTCCA-3′
	Probe: 5′-ACAACAGATCGCGTGATGACCGTCTC-3′
Mouse *Dynamin 2*	
	Forward: 5′- AGAGGAGACCGAGCGAAT-3′
	Reverse: 5′- CATGGTTTGTGTTGATGTACGAC-3′
	Probe: 5′- CCTACATCAGGGAGCGAGAAGGGA-3′

## RESULTS

### Synthesis of *R*- and *S*-5′-methyl DNA nucleoside phosphoramidites starting from diacetone glucose

**Figure 2.**
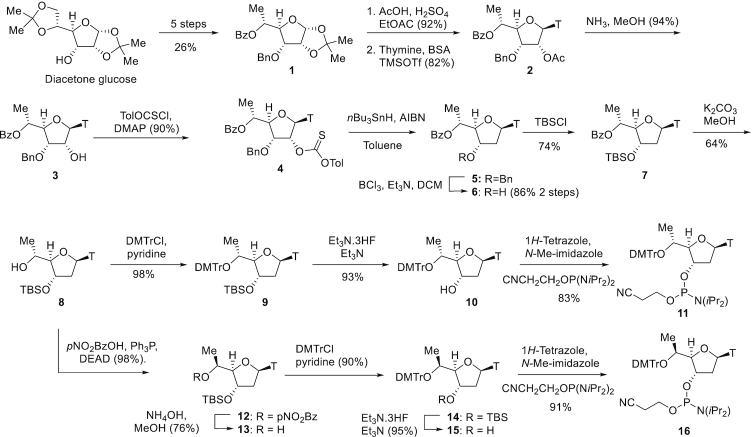
Synthesis of *R*- and *S*-5′-Me DNA nucleoside phosphoramidites

The synthesis of *R*- and *S*-5′-methyl DNA thymidine phosphoramidites is described in Figure 2. The sugar intermediate 1 was prepared in multi-gram quantities in 5 steps starting from diacetone glucose and was the key intermediate for the stereoselective synthesis of 5′-methyl DNA nucleosides (Supplementary Scheme S1). 1 was subjected to acetolysis using acetic acid and concentrated sulfuric acid in ethyl acetate to provide the corresponding bis-acetate, that was further subjected to the Vorbruggen reaction (27) using per-silylated thymine to provide nucleoside 2. The 2′-*O*-acetate group was selectively removed in the presence of the benzoyl group using cold methanolic ammonia, followed by a Barton deoxygenation (28) to provide the 2′-deoxynucleoside 5. The 3′-*O*-benzyl group was deprotected using boron trichloride to provide 6, which was further protected as the 3′-*O*-(*tert*-butyldimethylsilyl) ether to provide 7. The 5′-*O*-benzoyl group was deprotected using potassium carbonate in methanol to provide 8 followed by protection as the 4,4′-dimethoxytrityl (DMTr) ether to provide 9. Removal of the 3′-*O*-(*tert*-butyldimethylsilyl) protecting group provided nucleoside 10, which was phosphitylated to provide the desired *R*-5′-methyl DNA thymidine phosphoramidite 11. The stereochemistry at the 5′-position was confirmed by x-ray crystallography following deprotection of the 3′-*O*-*tert*-butyldimethylsilyl group in nucleoside 8 (Supplementary Figure S1 and Tables S1–S6).

Synthesis of the *S*-5′-methyl DNA thymidine phosphoramidite was accomplished by inversion of the secondary 5′-hydroxyl group in 8 using the Mitsunobu reaction (29) with *p*-nitrobenzoic acid. Deprotection of the 5′-*O*-(*p*-nitrobenzoyl) group provided 13 which was further protected as the DMTr ether and subjected to desilylation to remove the 3′-*O*-(*tert*-butyldimethylsilyl) protecting group to provide 15. Phosphitylation of 15 provided the *R*-5′-Me DNA thymidine phosphoramidite 16. Synthesis of the 5-Me cytosine, adenosine and guanosine analogs was accomplished using analogous procedures as described in Supplementary Schemes S2–S7. Synthesis of the 5′-ethyl nucleoside monomers (Supplementary Schemes S8–S10) and 5′-allyl nucleoside monomers (Supplementary Schemes S11-S14) was also accomplished as described in the supplementary information (30-33).

### Effect of site-specific incorporation of *R*- and *S*-5′-methyl DNA on duplex thermal stability, ASO activity and toxicity in cells

We evaluated the effect of replacing each PS deoxynucleotide in the gap region of ION 558807 – a model hepatotoxic ASO – with *R*- and *S*-5′-methyl DNA monomers. The *R*- and *S*-5′-methyl DNA phosphoramidites were incorporated into gapmer ASOs using standard automated chemistry and the resulting ASOs were evaluated in duplex thermal stability measurements for RNA-binding affinity and in cells for effects on antisense activity and cytotoxicity.

**Figure 3.**
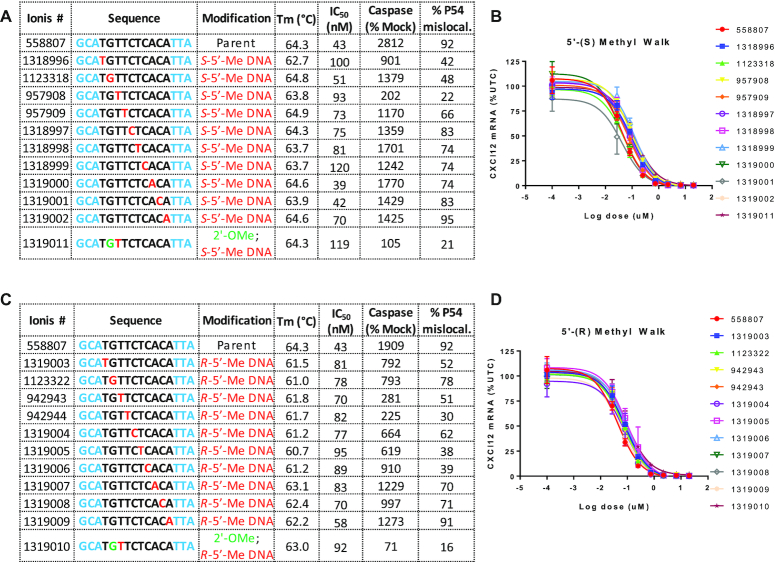
(**A**) 5′-*S*-Me DNA (**C**) 5′-*R*-Me DNA monomers were walked across the gap region and effect on duplex stability versus complementary RNA, antisense activity in NIH3T3 cells and cytotoxicity as measured by caspase activation in Hepa1-6 cells were determined. P54nrb mislocalization was detected by immunofluorescence staining in Hela cells. The percentage of cells containing mis-localized P54nrb protein was calculated based on manual counting of ∼100 cells. Dose response curves for reducing CXCl12 mRNA in NIH3T3 following delivery of (**B**) 5′-*S*-Me DNA and (**D**) 5′-*R*-Me DNA ASOs cells by electroporation. Blue letters indicate constrained Ethyl (cEt), black indicate DNA and red indicate 5′-alkyl DNA nucleotides. All ASOs were fully phosphorothioate (PS) modified.

Incorporation of a single *S*-5′-methyl DNA modification in the gap resulted in minimal changes in duplex thermal stability (average –0.3°C/mod.) relative to the control ASO (Figure 3A). These results are consistent with our previous data that *S*-5′-methyl LNA exhibited significantly improved duplex forming abilities as compared to the *R*-5′-methyl LNA stereoisomer (11). In contrast, incorporation of a single *R*-5′-methyl DNA modification in the gap resulted in a modest destabilization (average –2.6°C/mod.) relative to the parent control ASO (Figure 3C). However, all the modified ASOs exhibited excellent duplex stability when paired with complementary RNA suggesting no significant changes in duplex forming ability of these ASOs.

The modified ASOs were also evaluated for antisense activity in mouse 3T3 cells following delivery by electroporation where all of them exhibited comparable activity (within 2-fold IC_50_) as the parent ASO (Figure 3A–D). The ASOs were also evaluated for cytotoxicity in Hepa1-6 cells using a caspase assay as described previously (5). The parent model toxic ASO ION558807 exhibited dramatic increase in caspase activation that was mitigated by introducing a *S*-5′-Me DNA monomer at position 3 in the gap or with *R*-5′-Me DNA monomer at position 4 in the gap. This positional preference was different from 2′-analogs such as OMe which mitigate toxicity when present at position 2 in the DNA gap (5). The preference for positions 3 and 4 for mitigating toxicity was also borne out in the P54 mislocalization assay where introducing *S*-5′-Me DNA at position 3 and *R*-5′-Me DNA at position 4 showed reduced nucleolar mislocalization of P54 (Figure 3A, C and Supplementary Figure S2). We also evaluated ASOs with OMe at gap position 2 and 5′-Me DNA at gap position 3 but combining the two modifications did not provide a benefit over using the modifications individually, and as a result, these ASOs were not evaluated any further.

### Effect of site-specific incorporation of 5′-methyl, 5′-allyl and 5′-ethyl DNA on potency and hepatotoxicity in mice

**Figure 4.**
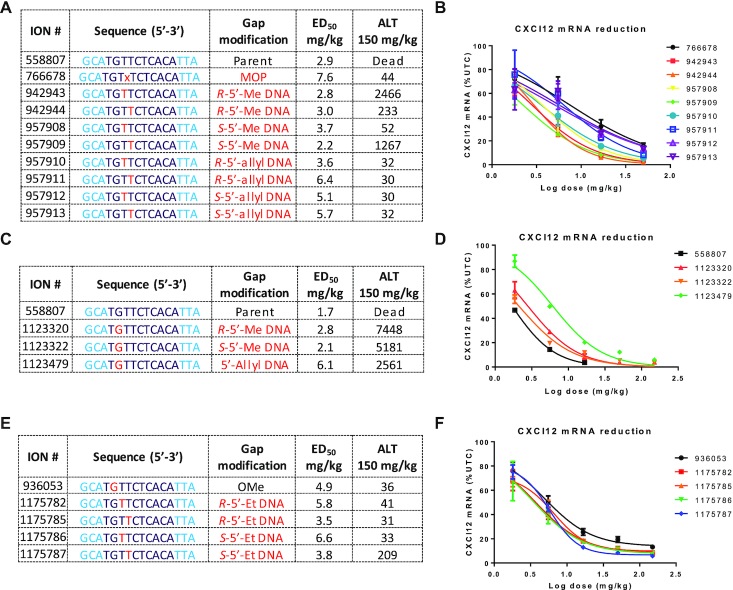
Characterizing the effect of inserting *R*- and *S*-5′-Me DNA and 5′-allyl DNA monomers at (**A** and **B**) positions 3 and 4 and (**C** and **D**) position 2 in the DNA gap on potency as determined by the dose required to reduce CXCL12 mRNA by 50% in the liver relative to untreated control animals (ED_50_) and hepatotoxicity as measured by increases in plasma ALT (IU/l) in mice. (**E** and **F**) Characterizing the effect of introducing *R*- and *S*-5′-ethyl DNA monomers at positions 3 and 4 in the DNA gap on potency and hepatotoxicity in mice. For ED_50_ determination, mice (Balb/c, *n* = 3/group) were injected subcutaneously with ASO (50, 16.7, 5.6 and 1.9 mg/kg) and euthanized after 72 hours. Livers were harvested and reduction in CXCl12 mRNA was measured using qRT-PCR. For plasma ALT, mice (Balb/c, *n* = 3/group) were injected subcutaneously with 150 mg/kg of ASO and euthanized after 72 h and ALT values were measured on a clinical analyzer. Blue letters indicate constrained Ethyl (cEt), black indicate DNA and red indicate 5′-alkyl DNA nucleotides. All ASOs were fully phosphorothioate (PS) modified.

ASOs with different 5′-alkyl DNA analogs were evaluated in mice to characterize the effect of the size, configuration and placement of the 5′-alkyl DNA monomers on potency and hepatotoxicity in mice. The model toxic parent ASO 558807 and a variant with 3-methoxypropyl phosphonate (MOP) at gap position 2 were included as controls. Mice were injected subcutaneously with increasing doses of ASOs substituted with *R*- or *S*-5′-methyl DNA, *R*- or *S*-5′-allyl DNA at gap positions 3 and 4. The animals were sacrificed 72 h after injection and livers were homogenized and reductions in CXCl12 mRNA were determined by quantitative RT-PCR. The levels of alanine aminotransferase (ALT) in plasma were also measured at the time of sacrifice. Most of the *R*- and *S*-5′-methyl DNA modified ASOs exhibited comparable potency but significantly reduced toxicity even at the highest dose of 150 mg/kg, as compared to the parent ASO 558807 which can be lethal when administered at doses above 50 mg/kg (Figure 4A and B). Interestingly, ASOs with *R*-5′-Me DNA at position 3 or *S*-5′-Me at position 4 showed elevations in ALT suggestive of a configuration dependent positional preference for these modifications. In contrast, the *R*- and *S*-5′-allyl DNA modified ASOs exhibited no ALT elevations even at the highest dose of 150 mg/kg but were slightly less potent than the parental ASO.

We also determined the effect of introducing *R*- and *S*-5′-methyl and 5′-allyl DNA at gap position 2 to confirm if these ASOs would be hepatotoxic in mice as predicted by the cellular cytotoxicity assays (Figure 3A and C). Substitution at position 2 with 2′-OMe was previously shown to be beneficial for broadly reducing toxicity of several hundred cEt gapmer ASOs (5). The 5′-allyl monomer was synthesized as a mixture of isomers as were not able to introduce this substituent stereoselectively into 2′-deoxyguanosine nucleosides (Supplementary Scheme S14). Mice were injected subcutaneously with increasing doses of ASOs and sacrificed 72 hours after injection. Both 5′-methyl DNA ASOs were found to be toxic in mice suggesting that these analogs have a different positional preference in the gap for reducing toxicity as compared to 2′- and backbone-modified analogs (Figure 4C and D). Consistent with the animal data, ASOs with *R*- and *S*-5′-allyl DNA are positions 3 and 4 showed reduced protein binding and P54 mislocalization relative to ASOs with 5′-allyl DNA at position 2 or bicycloDNA at positions 3 and 4 (Supplementary Figures S3 and S4).

Lastly, given the stronger effect on reducing toxicity observed with the 5′-allyl DNA analog, we investigated whether 5′-ethyl DNA could improve potency relative to the 5′-allyl DNA but reduce toxicity sometimes observed with high doses of ASOs with 5′-methyl DNA analogs. The *R*- and *S*-5′-ethyl nucleoside phosphoramidites were synthesized as described in Supplementary Schemes S11–S14. The monomers were incorporated at positions 3 and 4 in the gap region of ASO 558807 and the modified ASOs were evaluated for potency and toxicity in mice. Both *R*- and *S*-5′-ethyl DNA reduced the hepatotoxicity observed with the parent ASO and, when placed at gap position 4, were slightly more potent than the control ASO 936053 that contains a 2′-OMe at gap position 2 of parental ASO 558807 (Figure 4E and F).

### Effect of site-specific incorporation of 5′-methyl and 5′-ethyl DNA on potency and hepatotoxicity for multiple hepatotoxic ASO sequences

**Figure 5.**
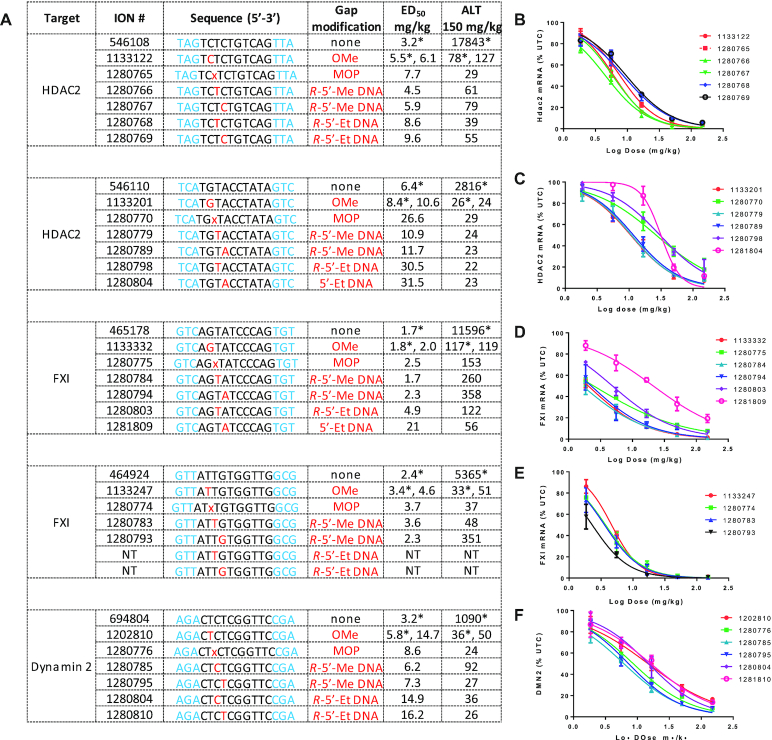
(**A**) Comparing the effect of introducing 2′-OMe, MOP, *R*-5′-methyl and *R*-5′-ethyl DNA modifications on potency and hepatotoxicity of ASOs targeting HDAC2, FXI and Dynamin 2 mRNA (Average ALT = 27 ± 5 IU/l for the PBS group). (**B–F**) Dose response curves for reducing the target mRNA in the liver in mice. Mice (Balb/c, *n* = 3/group) were injected subcutaneously with ASO (150, 50, 16.7, 5.6 and 1.9 mg/kg) and euthanized after 72 h. Livers were harvested and reduction in the levels of target mRNAs was measured using qRT-PCR. Plasma ALT values were measured on a clinical analyzer at study termination. Data indicated with asterix were collected from a different experiment using the same study design as indicated above. Blue letters indicate constrained Ethyl (cEt), black indicate DNA and red indicate 5′-alkyl DNA nucleotides. All ASOs were fully phosphorothioate (PS) modified.

Given that ASOs with *R*-5′-alkyl DNA were slightly more potent than ASOs with *S*-5′-alkyl DNA substitutions and because of the slightly shorter synthetic route to the *R*-5′-Me DNA monomers, we determined the effect of introducing *R*-5′-methyl DNA and *R*-5′-ethyl DNA in additional toxic ASO sequences. We picked five hepatotoxic ASOs targeting mouse FXI, HDAC2 and Dynamin2 mRNAs for evaluation in mice and compared these to control ASOs with 2′-OMe or MOP substitution at gap position 2 (Figure 5A). The parent 3–10–3 gapmer ASOs were not included in this evaluation as they were previously shown to be toxic in mice (5).

For the first set of ASOs targeting HDAC2 mRNA, mice were injected subcutaneously with increasing doses of the gap modified ASOs. Animals were sacrificed 72 h after ASO administration and reductions in HDAC2 mRNA in the liver were quantified by qRT-PCR. Plasma ALT levels were also measured at the time of sacrifice. All the gap-modified ASOs showed no overt hepatotoxicity when dosed up to 150 mg/kg, but the ASO with *R*-5′-methyl DNA at gap position 3 showed the best potency (Figure 5A and B). Similar results were obtained for the second HDAC2 sequence where the ASO with *R*-5′-methyl DNA at gap position 3 and the ASO with 2′-OMe at gap position 2 showed the best potency (Figure 5C).

We next evaluated ASOs targeting mouse FXI mRNA. Mice were injected subcutaneously with increasing doses of the modified ASOs and reduction of liver FXI mRNA were quantified post sacrifice. As seen with the HDAC2 ASOs above, all the gap modified ASOs showed no overt hepatotoxicity at the highest dose of 150 mg/kg (Figure 5A). In the first set, ASOs with 5′-alkyl DNA showed comparable potency to the ASO with 2′-OMe at position 2 in one sequence (Figure 5D). For the second toxic FXI ASO, we only evaluated ASOs with *R*-5′-methyl DNA substitution and the best potency was observed when this modification was incorporated at position 4 in the deoxynucleotide gap (Figure 5E).

Lastly, we evaluated the effect of gap modifications on potency and toxicity using a gapmer ASO targeting Dynamin2 mRNA (Figure 5A). Mice were injected with increasing doses of the ASOs and mRNA reduction in the liver and plasma ALT levels were measured after sacrifice. In this series, ASOs with *R*-5′-methyl at gap positions 3 and 4 exhibited excellent potency while ASOs with *R*-5′-ethyl DNA at the same position were less active (Figure 5F). All ASOs were safe when dosed at 150 mg/kg.

### Effect of *R*- and *S*-5′-methyl DNA on RNaseH1 cleavage patterns

**Figure 6.**
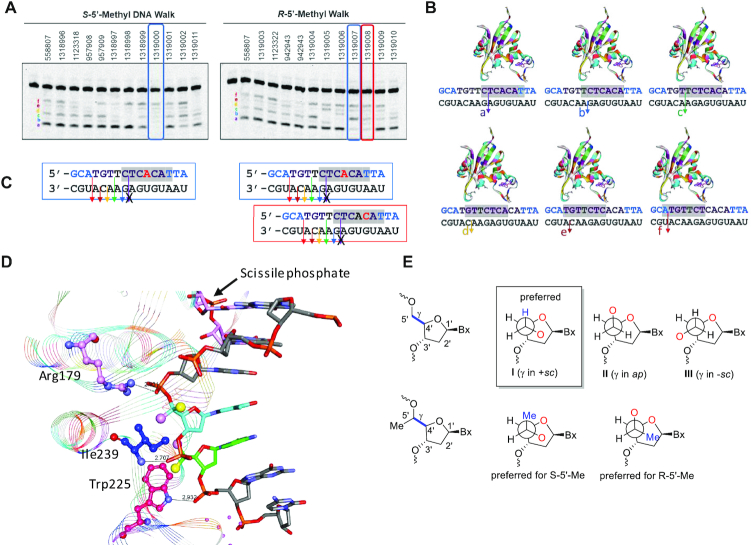
(**A**) Characterizing the effect of introducing *R-* and S-5′-Me DNA monomers in the gap on RNaseH1 cleavage patterns of ASO/RNA duplexes. (**B**) Structural model showing the 7-base footprint of the catalytic domain of recombinant human RNaseH1 for cleavage sites a-f on the ASO/RNA duplex. (**C**) Incorporation of *S*-5′-Me DNA at position 8 in the DNA gap ablates cleavage site **a** while incorporation of *R*-5′-Me DNA at position 8 and 9 ablates cleavage site **a** on the ASO/RNA duplex. (**D**) Structural model showing how 5′-Me substituents can modulates important backbone contacts between Arg 179, Ile239 and Trp225 on human RNAseH1 and the sugar-phosphate backbone of the ASO. (**E**) Newmann projections depicting how configuration of the 5′-methyl group can change the rotational preference around torsion angle γ of the sugar phosphate backbone. Blue letters indicate constrained Ethyl (cEt), black indicate DNA and red indicate 5′-alkyl DNA nucleotides. All ASOs were fully phosphorothioate (PS) modified.

We examined the effect of introducing *R*- and *S*-5′-methyl DNA in the gap region of 558807 on RNaseH1 cleavage patterns by the recombinant full-length human enzyme (34). The ASOs were duplexed with a 5′-FAM labeled complementary RNA and the heteroduplexes were subjected to cleavage by RNaseH1. The cleavage products were separated on a denaturing gel and sites of cleavage on the RNA were determined. RNaseH1 produces six distinct cleavage sites on the RNA for the parent ASO 558807 which were labeled a–f respectively (Figure 6A). Introducing *R* and *S*-5′-methyl DNA had a position specific effect on cleavage patterns that can be better understood by looking at the position of each modification within the distinct but over-lapping seven-nucleotide foot-print of the catalytic domain of RNaseH1 for each cleavage site on the RNA (Figure 6B) (35).

The effect of the modifications on cleavage patterns can be exemplified by looking at the sites where *R*- and *S*-5′-methyl DNA cause ablation of the main cleavage site a on the RNA (Figure 6C). *R*-5′-methyl DNA causes ablation of cleavage site a when introduced at positions 8 or 9 in the DNA gap (positions 4 and 5 in the seven-nucleotide footprint). In contrast, *S*-5′-methyl DNA causes ablation of cleavage site a only when incorporated at position 8 in the DNA gap (position 4 in the seven-nucleotide footprint).

The results from the cleavage analysis can be rationalized by examining the crystal structure of the catalytic domain of human RNaseH1 (Figure 6D) (36). The enzyme makes three important contacts with the DNA backbone with Arg179 in the phosphate binding pocket at position 3 in the footprint, backbone amide of Ile239 at position 4 in the footprint and the ring-NH of Trp225 at position 5 in the footprint. Introducing *R*- and *S*-5′-methyl DNA at position 4 can interfere with two important contacts (Arg179 and Ile 239) resulting in ablation of cleavage. Interestingly, introducing *R*-5′-methyl DNA at position 5 can interfere with the backbone contact made by Trp225 since this methyl group points in the direction of the indole ring. In contrast, the *S*-5′-methyl group points away from the indole ring and presumably does not interfere with this important contact at position 5 of the footprint.

It should also be noted that introducing substitution at the 5′-position of nucleoside monomers can cause conformational changes around torsion angle gamma (γ) of the sugar phosphate backbone (Figure 6E) (37). There are at least three distinct conformations around γ where the 5′-oxygen atom is either, in a *gauche* orientation with the ring oxygen atom (γ in +*sc* and *ap* ranges), or where the two oxygen atoms are in an *anti* orientation (γ in -*sc* range). Previous work has shown that introducing methyl groups at the 5′-position can alter conformational equilibrium around γ with +*sc* being the preferred orientation for *S*-5′-methyl and *ap* being preferred for *R*-5′-methyl (11,13). Thus, the effects on cleavage patterns could also result from changes in conformational preferences around the backbone torsion angles in addition to unfavorable steric contacts as discussed above.

## DISCUSSION

PS ASO therapeutics have made tremendous strides over the last decade. Six PS ASOs have been approved by regulatory agencies to date and an additional 45 PS ASOs are in clinical evaluation for disease indications ranging from rare genetic diseases to broad cardiovascular diseases (38). In parallel, significant advances have been made in our understanding of how PS ASOs interact with plasma, cell-surface and cellular proteins to further improve the profile of PS ASOs for therapeutic applications (39,40).

We recently showed that toxic cEt gapmer ASOs bind paraspeckle proteins such as P54nrb, FUS and PSF more tightly than non-toxic ASOs and cause their nucleolar mislocalization resulting in cytotoxicity in cells and hepatotoxicity in mice (5). We further showed that simple chemical strategies such as introducing 2′-OMe nucleosides or an alkyl phosphonate linkage at position 2 in the DNA gap can mitigate these toxicities and improve therapeutic index in animals (3). However, these strategies resulted in modest (2-fold) reductions in antisense potency for some ASO sequences. In this study, we examined if introducing small structural changes at other positions along the sugar-phosphate backbone can also enhance the TI of toxic cEt gapmer ASOs while maintaining potency relative to the parent ASO.

Given the precise nature of the SAR for mitigating toxicity (near positions 2–3 in the deoxynucleotide gap), we investigated the effect of introducing substitution at the 5′-position of nucleoside monomers. The 5′-position is an interesting site for introducing substitution as it is in close proximity of the PS linkage, and configuration of the substituent group can alter conformational dynamics around one or more backbone torsion angles of the sugar phosphate backbone. Interestingly, a substituent group at the 5′-position would be predicted to occupy similar chemical space as a 2′-substituent on the 5′-adjacent nucleotide in an ASO (Figure 1B).

The *R*- and *S*-5′-methyl DNA nucleosides were incorporated at every position of the DNA gap and the modified ASO were evaluated in duplex thermal stability experiments and for antisense activity and cytotoxicity in cells. In general, introducing either modification was well tolerated and resulted in modest reductions in duplex thermal stability versus complementary RNA (–0.3 to –2.3°C/modification). All ASOs also exhibited good activity in cells comparable to the parent ASO but only ASOs with modifications at positions 3 and 4 in the gap showed greatly reduced cytotoxicity.

To confirm these results in mice, we investigated the effect of introducing *R*- and *S*-5′-methyl DNA at positions 2, 3 and 4 in the gap on hepatotoxicity and potency in the liver. As suggested by the cellular cytotoxicity data, introducing 5′-alkyl DNA nucleosides at position 2 in the gap was not effective at mitigating hepatotoxicity in mice suggesting a change in positional preference from the 2′- and backbone-modifications investigated previously. Instead, greater mitigation in toxicity was observed by introducing 5′-alkyl DNA modifications at positions 3 and 4 in the gap.

We also examined if introducing larger 5′-alkyl substituents such as 5′-allyl DNA were more useful for mitigating toxicity. We found that while the larger substituents were more effective at ablating toxicity, they were ∼2-fold less potent than ASOs with 5′-methyl DNA. Furthermore, ASOs with *R*-5′-methyl DNA were slightly more potent than ASOs with *S*-5′-Me DNA, while preserving a strong effect on reducing toxicity. As a result, *R*-5′-Me DNA was chosen for broader evaluation in additional ASO sequences.

Given the strong effect of the 5′-allyl group for reducing hepatotoxicity, we also investigated if an intermediate sized substituent group such as 5′-ethyl could be more effective at reducing toxicity while preserving potency. Indeed, *R*-configured 5′-methyl and 5′-ethyl DNA analogs were broadly effective at mitigating toxicity for multiple ASO sequences targeting different mRNAs. However, ASOs with the larger 5′-ethyl group showed slightly reduced potency as compared to ASOs with *R*-5′-methyl DNA monomers. These data suggest that even small changes in the size of the substituent group can affect the biological profile of PS ASOs. These are remarkable observations given that ASOs are macromolecules with molecular masses close to 6 kDa.

We have previously shown that PS-ASOs bind proteins, and toxic PS-ASOs tend to bind more proteins more tightly, leading to mislocalization of paraspeckle proteins to the nucleolus, protein degradation, and apoptotic cell death (3,5). Introducing 2′-OMe at gap position 2, or the MOP neutral backbone at gap positon 3, all reduced protein binding and dramatically mitigated toxicity. We observed similar effects with 5′-alkyl DNA modified ASOs where introducing these modifications at positions 3 or 4, but not position 2, resulted in reduced protein binding and P54 mislocalization suggesting that 5′-alkyl DNA also reduce cytotoxicity by the same mechanism.

We also evaluated if small structural perturbations could influence the interactions of ASOs with RNaseH1. Indeed, site specific introduction of 5′-methyl DNA in the gap region produced configuration and position dependent changes in cleavage patterns. Superimposing the structural changes onto the crystal structure of the catalytic domain of RNaseH1 suggested that the *R*-5′-methyl group ablates cleavage when introduced at positions 4 and 5 of the seven-nucleotide footprint (35). In contrast, the *S*-5′-methyl group ablates cleavage only when present at position 4 of the footprint. Further structural analysis suggested that the *R*- and *S*-5′-methyl groups interfere with important contacts between the enzyme and the DNA backbone near position 4 of the footprint. In contrast, only the *R*-5′-methyl group had a steric clash with Trp225 near position 5 in the footprint, thus rationalizing the observed changes in cleavage patterns. The changes in RNaseH1 cleavage preferences suggest that proteins in biological systems are capable of detecting even small changes in ASO structure. Interestingly, inserting 5′-Me DNA at specific positions in the gap resulted in ablation/mitigation of the main cleavage site but this did not result in reduced activity in cells. While we did not evaluate cleavage rates in these experiments, it has been difficult to demonstrate a relationship between cleavages rates in the biochemical assay with antisense activity in cells, except when cleavage was ablated completely resulting in inactive ASOs. Indeed, this strategy was used to enhance allele selectivity of gapmer ASOs when targeting SNPs associated with expanded CAR transcripts in the *huntingtin* gene (10).

In conclusion, the strategy for the chemical optimization of ASO drugs continues to evolve. In the early days, chemical optimization was focused on enhancing the nuclease stability and pharmacokinetic properties of ASO drugs (41). This work was done in parallel with strategies to enhance the RNA-affinity by mimicking or locking the furanose ring in the RNA-like C3′-endo conformation. This work culminated in the identification of cEt gapmer ASOs that showed similar potency but reduced toxicity relative to LNA gapmers. Indeed, there are currently ∼15 cEt gapmers in clinical development demonstrating that it is possible to identify safe and effective cEt gapmer ASOs (38). However, identification of these medicines represents a significant challenge and several thousand gapmer ASO are screened to identify active and safe leads. To address these challenges, we recently reported that introducing 2′-modification at position 2 in the DNA gap or replacing the backbone PS with a neutral linkage could reduce toxicity while maintaining antisense potency.

In this report, we show that introducing substitution at the 5′-position of the nucleoside monomers can also mitigate toxicity, presumably by hindering access to the PS backbone or by changing the rotational preference around the C4′–C5-exocyclic bond. Interestingly, all the structural changes which mitigate toxicity cluster near nucleotides 2, 3, 4 on the 5′-side of the DNA gap suggesting that this region is an important structural recognition site for proteins that are involved in cellular toxicities. Thus, this work ushers a new era of chemical optimization with a focus on optimizing the therapeutic profile of the ASO as opposed to nuclease stability, RNA-affinity and pharmacokinetic properties. The 5′-methyl DNA modified ASOs exhibited excellent safety and antisense activity in mice highlighting the therapeutic potential of this class of nucleic acid analogs for next generation ASO designs.

## Supplementary Material

gkab047_Supplemental_File
