# Supplementary material for: Site-specific incorporation of 5′-methyl DNA enhances the therapeutic profile of gapmer ASOs
Source: Nucleic Acids Res. 2021 Feb 5;49(4):1828–39. doi: 10.1093/nar/gkab047 (PMC7913697; doi:10.1093/nar/gkab047)
Supplement: gkab047_Supplemental_File [file gkab047_supplemental_file.pdf]

`Supporting Information

Site-specific incorporation of 5’-methyl DNA enhances the therapeutic profile of gapmer ASOs

Guillermo Vasquez, Graeme Freestone, W. Brad Wan, Audrey Low, Cheryl Li De Hoyos, Jinghua Yu, Thazha P. Prakash, Michael E. Oestergaard, Xue-hai Liang, Stanley T. Crooke, Eric E. Swayze, Michael T. Migawa*, Punit P. Seth*

Ionis Pharmaceuticals, 2855 Gazelle Court, Carlsbad, CA, 92010, USA

*mmigawa@ionisph.com, tel 760-603-2713

*pseth@ionisph.com, tel 760-760-2587

Table of Contents

Content	Page #
Synthesis of Monomers	**S01-S69**
X-ray Structural Data	**S71-S81**
Analytical data for synthesized oligonucleotides	**S82-S83**
Protein binding and P54 mislocalization	**S84-S86**

1) Synthesis of Monomers

General. All reagents were purchased from commercial vendors and used without any further purification. Unless specified otherwise, glassware was dried in an oven and the reactions were carried out under an atmosphere of nitrogen. ^{1}H-NMR spectra were obtained on a Bruker 300 MHz instrument. Low resolution mass spectrometry analysis were carried out on an Agilent 1100 series LCMS system equipped with a S.E.D.E.R.E. (France) Sedex 75 Evaporative Light Scattering detector.

Scheme S1. Synthesis of 5'-(R)- and (S)-Methyl Thymidine Nucleosides.

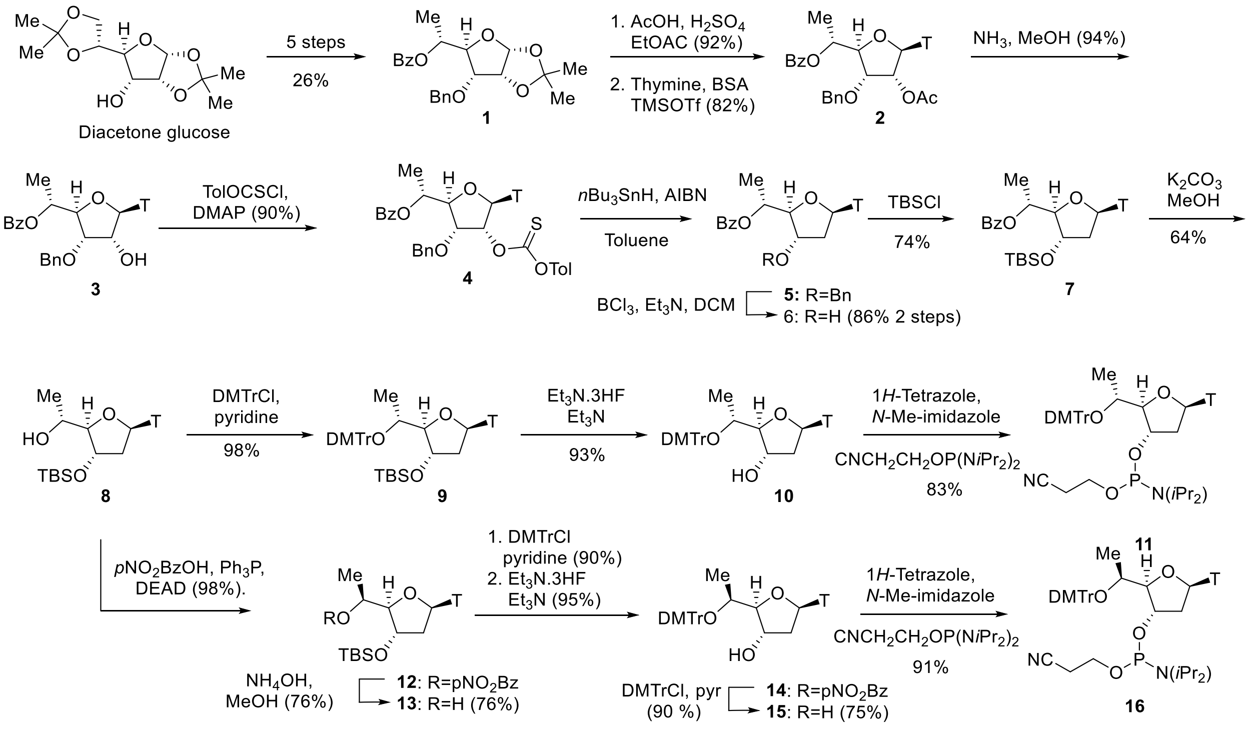

Compound 1: 1,2:5,6-Di-O-isopropylidene-alpha-D-allofuranose (100 g, 0.385 mol) was dissolved in dry *N*,*N*-dimethylformamide (1L) and the solution was cooled in an ice bath. NaH (23 g, 0.577 mol) was added to the above solution carefully in portions over 15-20 minutes. The reaction mixture was stirred in an ice bath for 30 minutes and BnBr (50.25 mL, 0.423 mol) was introduced slowly. The reaction was warmed to room temperature and stirred for 12 hours. The

reaction was quenched with ice water (2 L) and extracted with ethyl acetate (2 x 800 mL). The resulting ethyl acetate solution was dried over Na_2SO_4 and concentrated to dryness. The crude product was purified by silica gel column chromatography and eluted with 20% ethyl acetate in hexanes. Desired product was obtained as white solid (109 g, 81%). ^{1}H NMR (300 MHz, $CDCl_3$) d 7.29-7.47 (m, 5H), 5.76 (d, *J*=3.77 Hz, 1H), 4.55-4.84 (m, 3H), 4.38 (dt, *J*=3.11, 7.02 Hz, 1H), 4.15 (dd, *J*=3.20, 8.67 Hz, 1H), 3.94-4.06 (m, 2H), 3.89 (dd, *J*=4.62, 8.57 Hz, 1H), 1.60 (s, 3H), 1.29-1.42 (m, 9H); ^{13}C NMR (75 MHz, $CDCl_3$) d 137.5, 128.4, 128.2, 128.0, 112.9, 109.7, 103.9, 78.0, 77.8, 77.5, 74.8, 72.2, 65.0, 26.8, 26.6, 26.2, 25.1; LRMS (ES, positive): m/z calcd for $C_{19}H_{26}O_6$: 350.2, found 373.1 $[m+23]^+$

Water (55 mL) was added to a solution of1,2:5,6-Di-O-isopropylidene-3-OBn-alpha-D-allofuranose (109 g, 0.31 mol) in acetic acid (500 mL). The mixture was warmed to 40 ^oC and stirred for 12 hours. Ethyl acetate (1 L) was added to the reaction and the organic phase was washed with brine, saturated $NaHCO_3$ (careful), brine, dried over Na_2SO_4 and concentrated to dryness. The crude product was obtained as light-yellow goo and used without any further purification (56.46 g, 59%). ^{1}H NMR (300 MHz, $CDCl_3$) d 7.29-7.42 (m, 5H), 5.77 (d, *J*=3.77 Hz, 1H), 4.79 (d, *J*=11.30 Hz, 1H), 4.62 (t, *J*=4.05 Hz, 1H), 4.56 (d, *J*=11.30 Hz, 1H), 4.09-4.15 (m, 1H), 3.98-4.06 (m, 1H), 3.89-3.97 (m, 1H), 3.65-3.73 (m, 2H), 2.57 (br d, *J*=2.83 Hz, 1H), 2.49 (br s, 1H), 1.60 (s, 3H), 1.37 (s, 3H); LRMS (ES, positive): m/z calcd for $C_{16}H_{22}O_6$: 310.1, found 333.1 $[m+23]^+$

The bis-alcohol (56.10 g, 0.18 mol) obtained above was dissolved in a mixture of dichloromethane (270 mL) and pyridine (147 mL) and the reaction was cooled in an ice bath. To this solution, a solution of TsCl (42 g, 0.22 mol) in dichloromethane (160 mL) was added slowly. The reaction was stirred in an ice bath for 3 hours and carefully quenched with saturated $NaHCO_3$. The mixture was extracted with dichloromethane, washed with water, brine, dried over Na_2SO_4 and concentrated to dryness (105 g brown oil). The tosylated sugar (105 g, 0.18 mol) obtained above was dissolved in THF (800 mL) and the reaction was cooled in an ice bath. Lithium aluminum hydride (25 g, 0.66 mol) was added slowly to the reaction over 15-20 minutes. The reaction was stirred in an ice bath for 2 hours and water (18 mL) was carefully added to quench the unreacted hydride. After 20 minutes of stirring, 4 N NaOH (18 mL) was

added and the reaction mixture was stirred for another 20 minutes. To the mixture, more water (54 mL) was added and the reaction was stirred for another 15 minutes. Diethyl ether (1.5 L) was added to the reaction and mixture was stirred for an hour. The mixture was filtered through a pad of celite and rinsed sequentially with diethyl ether, dichloromethane, ethyl acetate. The organic solution was concentrated to dryness. The crude product was purified by column chromatography (silica gel, eluting with ethyl acetate/hexanes and MeOH/dichloromethane). Desired product was obtained as light-yellow oil (37.4 g, 72%). ^{1}H NMR (300 MHz, $CDCl_3$) δ 7.29-7.48 (m, 5H), 5.74 (d, *J*=3.77 Hz, 1H), 4.52-4.82 (m, 3H), 3.98-4.18 (m, 2H), 3.82-3.96 (m, 1H), 2.15 (d, *J*=2.45 Hz, 1H), 1.61 (s, 4H), 1.37 (s, 3H), 1.23 (d, *J*=6.59 Hz, 3H); LRMS (ES, positive): m/z calcd for $C_{16}H_{22}O_5$: 294.1, found 317.1 $[m+23]^+$

The 5'-methyl sugar (35.4 g, 0.12 mol) obtained above was dissolved in dichloromethane (700 mL) and triethylamine (33.6 mL, 0.24 mol) and the reaction was cooled in an ice bath. BzCl (16.7 mL, 0.14 mol) was added slowly and the reaction mixture was warmed to room temperature and stirred for 12 hours. The reaction was quenched with MeOH (10 mL) and stirred for 30 minutes. The dichloromethane solution was washed with 1 N HCl aqueous solution, saturated sodium bicarbonate, brine, dried over Na_2SO_4 and concentrated to dryness. The crude product was purified by column chromatography (silica gel, eluting with ethyl acetate/hexanes). Desired product was obtained as white solid (38.9 g, 81%). ^{1}H NMR (300 MHz, $CDCl_3$) δ 7.90-7.98 (m, 2H), 7.50-7.59 (m, 1H), 7.34-7.44 (m, 2H), 7.31 (s, 5H), 5.65-5.84 (m, 1H), 5.26-5.51 (m, 1H), 4.78 (dd, *J*=1.04, 11.77 Hz, 1H), 4.63 (t, *J*=3.96 Hz, 1H), 4.52 (dd, *J*=1.32, 11.68 Hz, 1H), 4.21 (ddd, *J*=1.13, 4.10, 8.71 Hz, 1H), 3.92 (ddd, *J*=1.32, 4.43, 8.76 Hz, 1H), 1.51-1.70 (m, 3H), 1.31-1.43 (m, 6H); ^{13}C NMR (75 MHz, $CDCl_3$) δ 165.7, 137.2, 132.8, 130.4, 129.7, 128.4, 128.2, 128.0, 113.0, 104.0, 79.9, 78.4, 77.4, 72.0, 70.1, 26.8, 26.6, 16.2; LRMS (ES, positive): m/z calcd for $C_{23}H_{26}O_6$: 398.2, found 421.1 $[m+23]^+$

Compound 2: Sugar (**1**) (150 g, 376 mmol) was dissolved in ethyl acetate (1.0 L) and the solution was stirred under nitrogen. Acetic anhydride (116 g, 107 mL g, 113 mmol, 3 eq.) was added followed by dropwise addition of sulfuric acid (7.4 g, 8.0 mL 125 mmol, 0.4eq). The reaction continued to stir for 3 hours at which time, TLC analysis (40% EtOAc in hexanes)

indicated that the reaction was completed. The reaction was concentrated to a reduced volume under reduced pressure, diluted with ethyl acetate and the organic layer was carefully washed with aqueous saturated sodium bicarbonate. The organic layer was separated, washed with brine, dried over Na_2SO, filtered, concentrated to a crude oil and evaporated three times with acetonitrile at 40°C and then dried under high vacuum over P_2O_5 for 16 hours to afford the desired product as a colorless oil (153 g, 92 % yield). This material was split into portions and used as is without further purification. ^{1}H NMR (300MHz, CHLOROFORM-d) δ = 8.10 - 7.87 (m, 2H), 7.60 - 7.31 (m, 4H), 7.27 - 7.13 (m, 2H), 5.43 - 5.06 (m, 2H), 4.65 - 4.36 (m, 2H), 4.33 - 4.25 (m, 1H), 4.23 - 4.08 (m, 1H), 4.04 (dd, *J*=3.5, 5.7 Hz, 1H), 2.19 - 1.98 (m, 4H), 1.52 - 1.18 (m, 3H). ^{13}C NMR (75MHz, CHLOROFORM-d) δ = 176.78, 166.05, 165.77, 137.02, 136.63, 133.23, 133.16, 133.10, 133.08, 130.20, 130.14, 130.00, 129.87, 129.80, 129.78, 129.66, 128.66, 128.59, 129.03 (br dd, *J*=9.0, 95.6 Hz, 1C), 128.46 (q, *J*=8.4 Hz, 1C), 128.28, 128.13 (q, *J*=5.6 Hz, 1C), 127.91, 101.97, 99.90, 96.87, 83.40, 82.80, 82.51, 79.16, 73.50, 73.13, 73.10, 73.05, 72.95, 72.42, 71.81, 71.31, 70.98, 70.87, 70.64, 20.86, 16.54, 16.48, 16.36, 20.74.

Thymine (31.9 g, 133 mmol, 1.3 eq) and N,O-Bis(trimethylsilyl)acetamide (339.0 mL, 1.387 mol, 4.0 eq) were added to a solution of the crude sugar above (153 g, 0.347mol) in acetonitrile (1.0 L) . The reaction was heated at 80°C for 20 minutes to obtain a clear solution, after which the solution was cooled in an ice bath and trimethylsilyl trifluoromethanesulfonate (123 g, 0.555mol, 1.6 eq) was added slowly over 10 minutes. The reaction was warmed to 80°C and stirred for 3 hours at which time TLC analysis (50% ethyl acetate in hexane/EtOAc) indicated that the reaction was complete. The solvent was evaporated under reduce pressure to obtain a crude oil which was dissolved in ethyl acetate (500 mL) and the organic layer was sequentially washed with water, saturated sodium bicarbonate, brine, dried over Na_2SO_4, filtered and concentrated under reduced pressure to obtain a crude oil. Purification by column chromatography (silica gel, eluting with 0-10% dichloromethane/acetone) afforded the desired product as a white solid. 146 g, 83 % yield. ^{1}H NMR (300MHz, CHLOROFORM-d) δ = 8.79 (br s, 1H), 8.04 (d, *J*=7.4 Hz, 3H), 7.64 - 7.54 (m, 1H), 7.53 - 7.41 (m, 3H), 7.41 - 7.23 (m, 7H), 6.93 - 6.77 (m, 1H), 6.09 (d, *J*=5.6 Hz, 1H), 5.46 (br dd, *J*=3.0, 6.7 Hz, 2H), 5.32 (t, *J*=5.6 Hz, 1H), 4.71 - 4.39 (m, 4H), 4.21 (dd, *J*=3.1, 4.4 Hz, 1H), 2.20 - 2.02 (m, 4H), 1.52 (s, 1H), 1.45 - 1.26 (m, 7H). ^{13}C NMR (75MHz, CHLOROFORM-d) δ = 170.29, 165.44, 163.38, 150.16,

136.86, 134.94, 133.65, 129.61, 128.84, 128.59, 128.26, 128.10, 111.70, 86.90, 84.34, 75.11, 73.82, 73.11, 70.25, 20.75, 16.12, 11.74; LCMS (ESI) m/z: [M+H] calcd, 508.2; found, 509.2

Compound 3. A solution of methanolic ammonia (7.0 M, 229 mL, 1.606 mol, 6.0 eq) was added to a solution of Compound **2** (139.30 g, 0.273 mol) in methanol (150.0 mL) that was previously cooled in an ice bath. The reaction was stirred at 0°C for 16 hours, and then evaporated under reduce pressure to an oil while maintaining the temperature around 0°C. The crude oil was purified by flash chromatography to afford the desired product as a white solid (126 g, 94 % yield). ^{1}H NMR (300MHz, CHLOROFORM-d) δ = 9.53 (br s, 1H), 8.00 (d, *J*=7.3 Hz, 2H), 7.65 - 7.54 (m, 1H), 7.52 - 7.41 (m, 2H), 7.41 - 7.26 (m, 6H), 6.85 (s, 1H), 5.98 (d, *J*=5.3 Hz, 1H), 5.42 (br dd, *J*=3.0, 6.6 Hz, 1H), 4.82 - 4.60 (m, 2H), 4.30 - 4.20 (m, 3H), 2.13 (s, 1H), 1.52 (s, 1H), 1.42 - 1.23 (m, 7H). ^{13}C NMR (75MHz, CHLOROFORM-d) δ = 165.51, 163.64, 150.93, 136.69, 134.82, 133.64, 129.56, 128.88, 128.68, 128.36, 128.19, 111.53, 88.40, 84.41, 75.94, 74.03, 72.69, 70.51, 16.33, 11.70; LCMS (ESI) m/z: [M+H] calcd, 466.2; found, 467.2

Compound 4. Compound **3** (120 g, 0.257mol) and 4-dimethylaminopyridine (62.9 g, 0.514 mol, 2.0 eq) were dissolved in anhydrous acetonitrile (360 mL) and the solution was cooled in an ice bath. O-4-methylphenyl chlorothioformate (46.70 mL, 0.309 mol, 1.2 eq) was added dropwise to the solution and the ice bath was removed. The reaction was stirred for 2 hours during which the reaction gradually warmed up to room temperature. The reaction was monitored by TLC (70% ethyl acetate in hexanes) to ensure conversion of the starting material. The solvent was removed under reduced pressure and the residue was partitioned between ethyl acetate and water. The aqueous layer was removed, and the organic layer washed successively with 10% HCl, saturated sodium bicarbonate, and brine. The organic layer was dried over Na_2SO_4, filtered and evaporated under reduce pressure. Purification by flash chromatography (silica gel, eluting with 70% diethyl ether/hexanes, followed by 100% ethyl acetate) afforded the desired product as a white solid. 143 g, 90 % yield. ^{1}H NMR (300MHz, CHLOROFORM-d) δ = 8.90 (s, 1H), 8.15 - 7.98 (m, 2H), 7.63 - 7.52 (m, 1H), 7.47 - 7.30 (m, 7H), 7.29 - 7.12 (m, 2H), 6.99 - 6.89 (m, 2H), 6.84 (d, *J*=1.1 Hz, 1H), 6.25 (d, *J*=6.0 Hz, 1H), 5.83 (t, *J*=5.8 Hz, 1H), 5.46 (dd, *J*=2.8, 6.7 Hz,

1H), 4.82 - 4.58 (m, 3H), 4.34 - 4.08 (m, 1H), 2.37 (s, 3H), 1.49 - 1.24 (m, 6H). ^{13}C NMR (75MHz, CHLOROFORM-d) δ = 194.84, 165.46, 163.42, 151.31, 150.18, 136.87, 136.74, 135.34, 133.67, 130.19, 129.49, 128.80, 128.61, 129.76, 128.30, 128.17, 121.26, 111.86, 86.67, 84.82, 81.55, 74.59, 73.39, 70.49, 21.00, 16.07, 11.76; LCMS (ESI) m/z: [M+H] calcd, 616.2; found, 617.2

Compound 5. A solution of azobisisobutyronitrile (AIBN) (4.36 g, 0.292 mol) and tributyltin hydride (156.40 mL, 0.583 mol) in toluene (200 mL) was added slowly to a degassed (with nitrogen) solution of compound **4** (144 g, 0.233 mol) in toluene (1L) at 80°C. After the addition was complete, the reaction was heated at 80°C for 1 hour and cooled to room temperature and the solvent was removed under reduced pressure. Purification by flash chromatography (silica gel, eluting with 0-40% ethyl acetate/hexanes) afforded 116g of 2'-deoxy nucleoside contaminated with some Bu_3SnH. ^{1}H NMR (300MHz, CHLOROFORM-d) δ = 8.71 (s, 1H), 8.09 - 7.88 (m, 2H), 7.66 - 7.53 (m, 1H), 7.51 - 7.42 (m, 2H), 7.39 - 7.25 (m, 6H), 7.10 - 6.87 (m, 2H), 6.86 - 6.61 (m, 1H), 6.32 (dd, *J*=5.5, 8.8 Hz, 1H), 5.57 - 5.32 (m, 2H), 4.69 - 4.48 (m, 2H), 4.42 - 4.28 (m, 1H), 4.19 (dd, *J*=2.5, 3.6) Hz, 1H), 2.57 (ddd, *J*=1.4, 5.5, 13.7 Hz, 1H), 2.26 (s, 1H), 1.99 (ddd, *J*=6.4, 8.6, 13.8 Hz, 1H), 1.75 (s, 1H), 1.64 - 1.47 (m, 1H), 1.46 - 1.31 (m, 6H), 1.11 - 0.83 (m, 1H); LCMS (ESI) m/z: [M+H] calcd, 450.2; found, 451.2.

Compound 6. Boron trichloride (BCl_3) (644 mL, 0.644 mol, 2.5 eq) was added slowly to a solution of compound **5** (110.6 g, 36.40 mmol) dissolved in anhydrous dichloromethane (500 mL) at -78°C. After the addition, the reaction was continued to stir at -78°C for 30 minutes. The reaction was quenched by slow addition of a mixture of Et_3N/MeOH/DCM/ (300 mL/300 mL/300 mL), always maintaining the temperature below -60°C. The mixture was then allowed to stir for an additional hour. This solution was slowly added to a cold saturated $NaHCO_3$ solution and stirred for 20 minutes. After separating the aqueous layer and washing the organics successively with saturated $NaHCO_3$ and brine, the organic layer was dried over Na_2SO_4, filtered and evaporated under reduced pressure. The crude material was purified by flash chromatography (silica gel, eluting with 0-5% methanol/dichloromethane) to afford the desired product as a white solid. 68 g, 77 % yield. ^{1}H NMR (300MHz, DMSO-d6) δ = 11.31 (s, 1H),

8.07 - 7.98 (m, 2H), 7.74 - 7.65 (m, 1H), 7.61 - 7.49 (m, 2H), 7.08 (d, *J*=1.1 Hz, 1H), 6.20 (t, *J*=7.0 Hz, 1H), 5.50 (d, *J*=4.7 Hz, 1H), 5.29 (dd, *J*=3.8, 6.6 Hz, 1H), 4.60 - 4.45 (m, 1H), 3.90 (t, *J*=3.4 Hz, 1H), 3.34 (s, 1H), 2.25 - 2.08 (m, 2H), 1.41 - 1.26 (m, 7H), 1.19 (t, *J*=7.3 Hz, 1H). ^{13}C NMR (75MHz, DMSO-d6) δ = 165.52, 163.88, 150.80, 135.44, 134.11, 130.04, 129.73, 129.44, 110.23, 87.95, 83.71, 71.08, 69.88, 16.46, 11.94; LCMS (ESI) m/z: [M+H] calcd, 360.1; found, 361.1

Compound 7. Compound **6** (62 g, 0.172 mol,) was dissolved in DMF (500 mL) and the solution was cooled in an ice bath and imidazole (23.4 g, 0.344 mol, 2eq) was added as a solution of DMF (50 mL) followed by *tert*-butyl-chlorodimethylsilane (31.6 g, 0.210 mol 1.2 eq). The reaction was stirred overnight, and then slowly poured on ice. Hexanes (800 mL) were added and the mixture was stirred for 90 minutes to affect a precipitate. The solvents were then decanted and the white solid was washed with water and hexanes twice. The white solid was co-evaporated with toluene under reduced pressure at 60°C twice to give the desired product as a white solid. 60 g, 74 % yield. ^{1}H NMR (300MHz, CHLOROFORM-d) δ = 8.20 (br s, 1H), 7.98 - 7.85 (m, 2H), 7.54 - 7.45 (m, 1H), 7.43 - 7.31 (m, 2H), 7.15 (s, 1H), 6.86 (s, 1H), 6.19 (dd, *J*=5.6, 8.4 Hz, 1H), 5.30 (br dd, *J*=3.8, 6.6 Hz, 1H), 4.55 - 4.44 (m, 1H), 3.95 - 3.88 (m, 1H), 2.26 - 2.12 (m, 1H), 2.04 - 1.85 (m, 1H), 1.53 (br s, 2H), 1.38 - 1.26 (m, 6H), 1.14 (s, 1H), 0.79 (s, 9H). ^{13}C NMR (75MHz, CHLOROFORM-d) δ = 165.56, 163.27, 150.04, 134.65, 133.64, 129.75, 129.48, 128.78, 111.19, 89.19, 84.63, 71.34, 70.64, 41.17, 25.67, 17.85, 16.75, 11.83; LCMS (ESI) m/z: [M+H] calcd, 474.2; found, 475.2

Compound 8. Compound **7** (60 g, 0.126 mol) was dissolved in methanol (1L). K_2CO_3 (70 g, 0.506 mol) was added and the reaction was stirred overnight. The solids were then removed by filtration and rinsed with DCM/MeOH (8/2). The filtrate was concentrated under reduced pressure and the resultant crude solid was suspend in ethyl acetate (500 mL), and washed successively with water, saturated $NaHCO_3$ and brine. The organics were dried over Na_2SO_4, filtered and evaporated under reduced pressure to obtained crude material that was purified by flash chromatography (silica gel, eluting with 40-60 % ethyl acetate/hexanes) afforded the desired product as a white solid. 32 g, 68 % yield. ^{1}H NMR (300MHz, CHLOROFORM-d) δ =

8.68 (br s, 1H), 7.24 (d, *J*=1.1 Hz, 1H), 7.17 (s, 1H), 5.97 (dd, *J*=6.0, 8.3 Hz, 1H), 4.48 - 4.41 (m, 1H), 4.01 - 3.91 (m, 1H), 3.69 (t, *J*=2.6 Hz, 1H), 2.80 (d, *J*=2.8 Hz, 1H), 2.36 - 2.23 (m, 1H), 2.03 (ddd, *J*=2.4, 6.0, 13.2 Hz, 1H), 1.81 (d, *J*=0.9 Hz, 3H), 1.66 (s, 1H), 1.17 (d, *J*=6.6 Hz, 3H), 0.80 (s, 9H). ^{13}C NMR (75MHz, CHLOROFORM-d) δ = 163.65, 150.37, 137.55, 111.04, 91.40, 87.60, 70.62, 67.62, 40.34, 25.70, 19.30, 17.83, 12.51; LCMS (ESI) m/z: [M+H] calcd, 370.2; found, 371.2

Compound 9. DMTrCl (52.2 g, 154 mmol) was added to a solution of compound **8** (16.3 g, 4.40 mmol) and 2,6-lutidine (20.20 mL, 176 mmol, 4 eq), in pyridine (250 mL) at room temperature and the solution was stirred at 45°C for 14 hours. The reaction was then quenched by the addition of methanol (10 mL), followed by water and ethyl acetate. The resulting suspension was extracted with ethyl acetate and the combined organics were washed with saturated $NaHCO_3$ and brine. After the removal of the solvents under reduced pressure, purification by flash chromatography (silica gel, eluting with 40-60% diethyl ether/hexanes) afforded the desired product as a white solid. 29 g, 97 % yield. ^{1}H NMR (300MHz, CHLOROFORM-d) d = 8.66 (s, 1H), 7.42 - 7.34 (m, 2H), 7.33 - 7.24 (m, 4H), 7.20 - 7.05 (m, 4H), 7.00 - 6.82 (m, 1H), 6.71 (dd, *J*=2.2, 8.9 Hz, 4H), 6.15 (dd, *J*=5.7, 8.6 Hz, 1H), 4.57 - 4.43 (m, 1H), 3.73 - 3.62 (m, 7H), 3.33 (s, 1H), 2.41 (s, 1H), 1.97 (s, 1H), 1.81 (s, 2H), 1.40 (s, 3H), 0.82 - 0.76 (m, 12H), 0.01 (d, *J*=3.2 Hz, 6H). ^{13}C NMR (75MHz, CHLOROFORM-d) d = 163.67, 158.62, 158.60, 150.24, 146.28, 136.85, 136.59, 135.49, 130.29, 130.24, 129.16, 128.12, 127.85, 127.78, 126.89, 120.22, 113.17, 113.12, 113.07, 110.97, 90.81, 86.57, 84.07, 71.46, 69.93, 55.23, 40.68, 25.71, 18.04, 17.86, 11.98. LCMS (ESI) m/z: [M+H] calcd, 672.3; found, 673.2

Compound 10. Triethylamine (7.77 mL, 55.7 mmol) was added to a solution of compound **9** (15 g, 22.3 mmol) in THF (60 mL). The reaction was then cooled in an ice bath and triethylamine trihydrofluoride (18.2 mL, 111 mmol, 5 eq) was added slowly. The reaction was warmed to room temperature and stirred overnight. The solvents were then removed under reduced pressure and the resultant material was purified by flash chromatography (silica gel, eluting with 50-70% ethyl acetate/hexanes) to afford the desired product as a white solid. 11.6 g, 93 % yield; LCMS (ESI) m/z: [M-H] calcd, 555.2; found, 554.2

Compound 11. 1*H*-Tetrazole (1.14 g, 16.4 mmol,0.8 eq) and 1-methylimidazole (0.408 mL, 5.14 mmol, 0.25 eq) were added to a solution of nucleoside **10** (15.0 g, 20.60 mmol) in DMF (200 mL) at room temperature under an atmosphere of nitrogen. 3-bis(diisopropylamino)-phosphanyloxypropanenitrile (9.80 mL, 30.8 mmol, 1.5 eq) was then added drop-wise and the reaction was stirred at room temperature for 3 hours. Water (10.0 mL) was added to quench the reaction. A 3:1 mixture of toluene/hexanes (80 mL) was added and the organic layer was washed (4x50 mL) with a 3:2 mixture of DMF/H_2O. The organics were then washed with saturated sodium bicarbonate solution, brine, dried over solid sodium sulfate and concentrated under reduced pressure to afford a white foam. Purification by flash chromatography (silica gel, 50g column, eluting with 50-60% ethyl acetate/hexanes + 1 % triethylamine) afforded the desired product as a white solid. 12.8 g, 82 % yield. ^{1}H NMR (300MHz, CHLOROFORM-d) d = 7.54 - 7.46 (m, 2H), 7.44 - 7.35 (m, 4H), 7.30 - 7.17 (m, 4H), 7.03 (d, *J*=8.7 Hz, 1H), 6.86 - 6.78 (m, 4H), 3.91 - 3.62 (m, 10H), 2.67 - 2.35 (m, 3H), 1.61 - 1.36 (m, 4H), 1.33 - 1.12 (m, 16H), 1.00 - 0.82 (m, 7H). ^{31}P NMR (121MHz, CHLOROFORM-d) d = 148.94 (s, 1P), 148.49 (s, 1P); LCMS (ESI) m/z: [M-H] calcd, 758.3; found, 757.3

Compound 12. Triphenylphosphine (Ph_3P) (16.7 g, 63.6 mmol, 4.0eq) and p-nitrobenzoic acid (10.6 g, 63.6 mmol, 4 eq) were added to a solution of compound **11** in anhydrous THF (50 mL). The reaction was cooled in an ice bath and diisopropyl azodicarboxylate (DIAD) (12.2 mL, 62.1 mmol, 4.0 eq) was added dropwise. The reaction was stirred for 4 hours, at which time the solvent was removed under reduced pressure and the residue was partitioned between ethyl acetate and water. The water layer was extracted twice more with ethyl acetate, and the organics were combined and washed successively with saturated $NaHCO_3$ and brine. The organic layer was then dried over Na_2SO_4, filtered and the solvent was removed under reduced pressure to a crude oil. Purification by flash chromatography (silica gel, eluting with 0-60% diethyl ether/hexanes) afforded the inverted ester as a white solid. 8.12 g, 98 % yield. ^{1}H NMR (300MHz, CHLOROFORM-d) d = 8.27 - 8.20 (m, *J*=8.9 Hz, 2H), 8.19 - 8.07 (m, *J*=8.8 Hz, 2H), 6.17 (t, *J*=6.6 Hz, 2H), 5.43 - 5.23 (m, 1H), 4.98 - 4.86 (m, 1H), 4.35 - 4.11 (m, 1H), 3.91 (dd, *J*=4.0, 5.8 Hz, 1H), 2.25 (br dd, *J*=3.9, 6.2 Hz, 1H), 2.14 - 1.95 (m, 1H), 1.88 (s, 3H), 1.44 (d,

J=6.5 Hz, 4H), 1.21 (d, *J*=6.3 Hz, 5H), 0.84 (s, 8H). ^{13}C NMR (75MHz, CHLOROFORM-d) d = 164.21, 163.59, 150.74, 150.04, 135.39, 135.34, 130.69, 123.71, 111.11, 88.52, 85.14, 72.01, 71.42, 70.12, 41.01, 25.70, 25.65, 21.96, 17.84, 17.17, 12.59; LCMS (ESI) m/z: [M+H] calcd, 519.2; found, 520.2

Compound 13. Ester **12** (8.67 g, 16.70 mmol) obtained above was dissolved in methanol (200 mL) and K_2CO_3 (9.22 g, 6.67 mmol, 4.0 eq) was added. The reaction was stirred for 14 hours and the solids were filtered and rinsed with DCM/MeOH (8/2) and the filtrate was evaporated under reduced pressure. The crude solid was suspended in ethyl acetate (100 mL), and washed successively with water (2x100 mL), saturated $NaHCO_3$, and brine. The organic layer was dried over Na_2SO_4, filtered and evaporated under reduced pressure to obtained crude material which was purified by flash chromatography (silica gel, 50-60 % ethyl acetate/hexanes) to afford the desired product as a white solid. 4.68 g, 76 % yield. ^{1}H NMR (300MHz, CHLOROFORM-d) d = 8.42 (br s, 1H), 7.32 - 7.27 (m, 1H), 7.18 (s, 1H), 6.05 (t, *J*=6.9 Hz, 1H), 4.38 - 4.31 (m, 1H), 3.83 (br d, *J*=3.0 Hz, 1H), 3.63 (t, *J*=3.2 Hz, 1H), 2.39 - 2.22 (m, 2H), 2.11 (br dd, *J*=3.4, 6.3 Hz, 1H), 1.87 - 1.81 (m, 3H), 1.58 (s, 3H), 1.26 - 1.15 (m, 4H), 0.81 (s, 9H). ^{13}C NMR (75MHz, CHLOROFORM-d) d = 163.53, 150.22, 137.17, 111.05, 90.65, 87.09, 72.73, 67.42, 40.12, 25.73, 20.54, 17.94, 12.56; LCMS (ESI) m/z: [M+H] calcd, 370.1; found, 371.1

Compound 14. DMTrCl (17.1 g, 50.5 mmol, 4 eq.) was added to a solution of compound **13** (4.68 g, 12.6 mmol), 2,6-Lutidine (5.88 mL, 50.5 mmol, 4 eq) and pyridine (150 mL) and the reaction was stirred for 14 hours at 45°C. The reaction was quenched by the addition of methanol (5 mL) and diluted with water and ethyl acetate. The organic layer was separated, and the aqueous layer was further extracted with ethyl acetate. The organic layers were combined and washed with saturated $NaHCO_3$ and brine. The solvent was removed under reduced pressure and the residue was purified by flash chromatography (silica gel, eluting with 40-60% diethyl ether/hexanes) afforded the desired product as a white solid. 7.64 g, 90 % yield. ^{1}H NMR (300MHz, CHLOROFORM-d) d = 8.44 (s, 1H), 7.90 (s, 1H), 7.51 - 7.20 (m, 11H), 6.86 (dd, *J*=3.3, 8.8 Hz, 4H), 6.35 (dd, *J*=5.7, 7.8 Hz, 1H), 4.36 - 4.28 (m, 1H), 3.86 - 3.73 (m, 7H), 3.63 (br dd, *J*=2.4, 6.4 Hz, 1H), 2.57 (s, 1H), 2.38 - 2.02 (m, 2H), 1.81 (s, 3H), 1.69 (br s, 2H), 1.31

(d, J=6.3 Hz, 1H), 1.03 (d, J=6.4 Hz, 3H), 0.94 - 0.82 (m, 9H). ^{13}C NMR (75MHz, CHLOROFORM-d) d = 163.64, 158.67, 158.65, 150.11, 145.90, 136.57, 136.29, 135.69, 130.48, 130.35, 128.32, 127.71, 126.99, 113.04, 113.03, 111.01, 91.84, 86.92, 84.62, 72.48, 69.93, 55.25, 41.85, 25.70, 18.91, 17.79, 12.30; LCMS (ESI) m/z: [M-H] calcd, 672.3; found, 671.2

Compound 15. Triethylamine (1.86 mL, 13.4 mmol 2.5eq) was added to a solution of compound **14** (3.60 g, 5.35 mmol) in THF (60 mL). The reaction was cooled in an ice bath and triethylamine trihydrofluoride (4.32 mL, 26.8 mmol, 5 eq) was added slowly. The reaction was warmed to room temperature and stirred for 14 hours after which the solvents were removed under reduced pressure and the crude residue was purified by flash chromatography (silica gel, eluting with 50-70% ethyl acetate/hexanes) to afford the desired product as a white solid. 2.24 g, 75 % yield. ^{1}H NMR (300MHz, DMSO-d6) d = 11.36 (s, 1H), 7.88 (s, 1H), 7.56 (s, 1H), 7.44 (d, J=7.5 Hz, 2H), 7.35 - 7.17 (m, 10H), 7.07 (d, J=8.7 Hz, 2H), 6.92 - 6.80 (m, 6H), 6.23 - 6.18 (m, 1H), 6.14 (br d, J=6.6 Hz, 1H), 5.21 (br d, J=4.8 Hz, 1H), 4.23 (br s, 1H), 3.80 - 3.51 (m, 12H), 2.50 (s, 10H), 1.76 (s, 1H), 1.67 (s, 3H), 1.13 (d, J=6.4 Hz, 2H), 0.70 (d, J=6.2 Hz, 3H). ^{13}C NMR (75MHz, DMSO-d6) δ = 164.22, 164.16, 158.62, 158.57, 158.28, 150.91, 150.85, 146.71, 140.70, 136.98, 136.78, 136.73, 135.99, 130.64, 130.51, 129.38, 128.36, 128.11, 127.88, 127.13, 113.49, 113.23, 110.10, 109.67, 90.80, 89.74, 86.38, 84.15, 83.83, 80.37, 71.70, 70.56, 70.20, 66.47, 55.49, 20.69, 18.06, 12.83, 12.56; LCMS (ESI) m/z: [M-H] calcd, 558.2; found, 557.4

Compound 16. 1*H*-Tetrazole (221 mg, 3.21 mmol,0.8 eq) and 1-methylimidazole (0.80 µL, 1.0 mmol, 0.25 eq) were added to a solution of compound **15** (2.24 g, 3.21 mmol) in DMF (40 mL) at room temperature. 3-bis(diisopropylamino)phosphanyloxypropanenitrile (1.91 mL, 6.01 mmol, 1.5 eq) was then added drop wise and the reaction was stirred at room temperature for 3 hours. Water (3.0 mL) was added to quench the reaction. A 3:1 mixture of toluene/hexanes (50 mL) was added and the organic layer was washed (4x50 mL) with a 3:2 mixture of DMF/H_2O. The organics were then washed with saturated sodium bicarbonate solution, brine, dried over solid sodium sulfate and concentrated under reduced pressure to a white foam. Purification by flash chromatography (silica gel, 50-60% ethyl acetate/hexanes + 1 % triethylamine) afforded the desired product as a white solid, 1.76 g, 91 % yield. ^{1}H NMR (300MHz, CHLOROFORM-d) d = 8.06 (s, 1H), 7.52 - 7.23 (m, 11H), 6.90 - 6.82 (m, 4H), 6.33 (t, J=6.2 Hz, 1H), 4.33 (br d,

J=6.4 Hz, 1H), 4.17 (d, *J*=7.0 Hz, 1H), 3.89 - 3.75 (m, 7H), 3.68 (s, 1H), 2.19 (s, 1H), 2.09 (s, 1H), 1.75 (s, 3H), 1.31 (t, *J*=7.1 Hz, 1H), 1.05 (d, *J*=6.4 Hz, 3H), 0.87 - 0.81 (m, 9H), 0.08 - 0.03 (m, 2H). ^{31}P NMR (121MHz, CHLOROFORM-d) δ = 149.24 (s, 1P), 149.11 (s, 1P); LCMS (ESI) m/z: [M-H] calcd, 758.3; found, 757.4

Scheme S2. Synthesis of 5'-(R)-Methyl Cytidine Nucleosides.

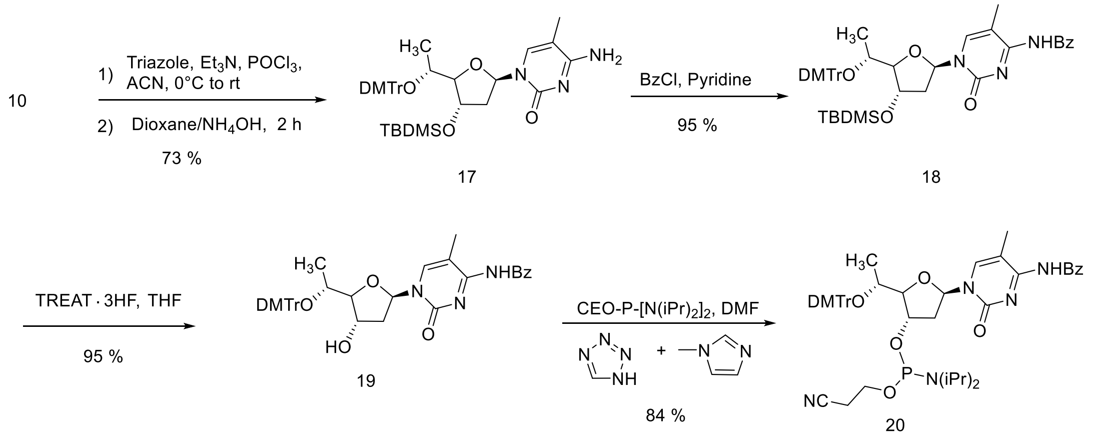

Compound 17. $POCl_3$ (4.88 mL, 53.3 mmol, 8 eq) was added dropwise to a suspension of 1,2,4-1*H*-triazole (20.7 g, 299 mmol, 34 eq.) in acetonitrile (150 mL) at room temperature and the reaction was stirred for 20 minutes. The reaction was cooled down in an ice bath and triethylamine (49.10 mL, 352 mmol, 40 eq.) added dropwise, after the addition was complete, the ice bath was removed and the reaction continued to stir for an additional 30 minutes at room temperature. The reaction was then cooled down to 0°C and a solution of compound **10** (4.48 g, 6.66 mmol) in acetonitrile (30 mL) was added dropwise. This was stirred at room temperature overnight. The next day, the reaction was concentrated to small volume under reduced pressure,

diluted with ethyl acetate and the organic layer was washed with aqueous saturated sodium bicarbonate (2x), water, brine and concentrated to a yellow oil. Without any further purification, the crude material was suspended in dioxane/NH_4OH (30 mL/10 mL) solution and stirred at room temperature for 2 hours. TLC in EtOAc/hexanes (8/2) indicated reaction was completed. The solvent was concentrated under reduced pressure and the resulting oil was diluted with ethyl acetate and washed with 1x200 ml water, 1x200 ml sat. $NaHCO_3$, 1x200 ml brine, and dried over Na_2SO_4, filtered and concentrated under reduce pressure to obtain a crude oil. The crude material was dissolved in DCM and purified by silica gel chromatography (silica gel, 40-100% EtOAc/hexanes) to afford the desired product as a white solid. 3.37 g, 73 % yield. ^{1}H NMR (300MHz, CHLOROFORM-d) d = 7.41 (d, *J*=7.2 Hz, 2H), 7.35 - 7.26 (m, 4H), 7.24 - 7.05 (m, 5H), 6.72 (dd, *J*=3.1, 8.9 Hz, 4H), 6.17 (dd, *J*=5.9, 7.6 Hz, 1H), 4.54 - 4.47 (m, 1H), 3.69 (d, *J*=2.2 Hz, 7H), 3.42 (br dd, *J*=3.4, 6.4 Hz, 1H), 2.29 - 2.03 (m, 1H), 1.76 (td, *J*=7.0, 13.8 Hz, 3H), 1.43 (s, 3H), 0.84 - 0.73 (m, 11H). ^{13}C NMR (75MHz, CHLOROFORM-d) d = 165.31, 158.56, 155.84, 146.37, 138.22, 136.96, 136.72, 130.35, 130.27, 128.18, 127.73, 126.81,113.07, 113.04, 101.35, 90.52, 86.46, 85.13, 71.38, 69.79, 55.23, 41.81, 25.73, 17.92, 17.85, 12.74; LCMS (ESI) m/z: [M-H] calcd, 671.3; found, 670.3

Compound 18. **(11a)** 4-amino-1-((2R,4S)-5-((R)-1-(bis(4-methoxyphenyl)(phenyl)methoxy)ethyl)-4-((tert-butyldimethylsilyl)oxy)tetrahydrofuran-2-yl)-5-methylpyrimidin-2(1H)-one (3.24 g, 4.81 mmol) was dissolved in anhydrous pyridine (20 mL) and the reaction was cooled in an ice bath. Benzoyl chloride (0.61 mL, 5.29 mmol, 1.1 3q.) was added dropwise and the reaction was stirred for room temperature for 14 hours at which point TLC analysis in (EtOAc/hexanes (6/4)) indicated that the reaction was complete. The reaction was cooled in an ice bath and 20 ml of water was slowly added followed by EtOAc (50 mL). The organic layer was washed with water, saturated $NaHCO_3$, brine and dried over Na_2SO_4 for 10 minutes, filtered and solvent was concentrated under reduce pressure to obtained crude oil. The crude material was dissolved in DCM and purified by chromatography (silica gel, 40-70% EtOAc/hexanes) afforded the desired product as a white solid. 3.57 g, 95 % yield.^{1}H NMR (300MHz, CHLOROFORM-d) δ = 8.23 - 8.16 (m, 5H), 7.45 - 7.26 (m, 18H), 7.21 - 7.04 (m, 19H), 6.79 - 6.65 (m, 11H), 6.16 (dd, *J*=5.7, 8.3 Hz, 2H), 5.99 (dd, *J*=6.0, 8.1 Hz, 1H), 4.60 - 4.52 (m, 1H), 4.51 - 4.42 (m, 1H), 3.98 (dd, *J*=2.8, 6.6 Hz, 1H), 3.73 - 3.64 (m, 17H), 3.36 (dd,

J=3.6, 6.4 Hz, 2H), 2.29 (br dd, *J*=6.0, 7.9 Hz, 2H), 2.21 - 2.05 (m, 3H), 2.05 - 1.95 (m, 4H), 1.92 - 1.68 (m, 4H), 1.59 (s, 5H), 1.51 (s, 2H), 1.35 -1.0 (m, 5H) ^{13}C NMR (75MHz, CHLOROFORM-d) δ = 179.59, 179.57, 159.67, 159.63, 158.65, 147.90, 147.33, 146.28, 139.47, 138.48, 137.20, 137.12, 136.85, 136.72, 136.56, 132.48, 132.42, 130.09 (dd, *J*=3.5, 26.4 Hz, 1C), 129.14, 128.13, 127.86, 127.80, 127.78, 127.10, 126.92, 113.18, 113.15,113.10, 111.99, 111.85, 91.58, 91.09, 88.16, 86.62, 84.72, 81.45, 71.46, 70.67, 69.91, 67.73, 55.25, 41.08, 40.65, 25.71, 19.49, 18.12, 17.86, 13.64,13.06; LCMS (ESI) m/z: [M-H] calcd, 775.3; found, 774.3

Compound 19. Triethylamine (1.60 mL, 11.5 mmol, 2.5 eq.) was added to a solution of compound **(12b)** N-(1-((2R,4R,5R)-5-((bis(4-methoxyphenyl)(phenyl)methoxy)methyl)-4-((tert-butyldimethylsilyl)oxy)tetrahydrofuran-2-yl)-5-methyl-2-oxo-1,2-dihydropyrimidin-4-yl)benzamide (3.57 g, 4.60 mmol) in tetrahydrofuran (45.0 mL). The reaction was cooled down in an ice bath and triethylamine trihydrofluoride (3.74 mL, 22.9 mmol, 5 eq.) was added slowly. The reaction was stirred for 16 hours at room temperature after which triethylamine (1.60 ml) was added to the reaction. The solvent was removed under reduced pressure to obtained crude solid that was dissolved in EtOAc and the organic layer was washed with water, sat. $NaHCO_3$, brine, dried over Na_2SO_4;, filtered and concentrated under reduce pressure to obtained crude oil. Purification by flash chromatography (silica gel, 20-100 % EtOAc/hexanes) afforded the desired product as a white solid. 2.90 g, 95 % yield. ^{1}H NMR (300MHz, DMSO-d6) δ = 12.99 (br s, 1H), 8.25 - 8.12 (m, 3H), 8.07 (br s, 2H), 7.73 - 7.43 (m, 8H), 7.40 - 7.16 (m, 13H), 7.11 - 7.03 (m, 6H), 6.93 - 6.79 (m, 9H), 6.29 - 6.12 (m, 3H), 5.34 (d, *J*=4.7 Hz, 1H), 5.23 (d, *J*=4.3 Hz, 1H), 5.08 (d, *J*=4.8 Hz, 1H), 4.41 - 4.05 (m, 1H), 3.83 - 3.63 (m, 14H), 2.32 - 2.08 (m, 3H), 2.07 - 1.93 (m, 4H), 1.83 (s, 1H), 1.63 (s, 2H), 1.24 - 1.09 (m, 4H), 0.80 (br d, *J*=6.2 Hz, 2H).^{13}C NMR (75MHz, DMSO-d6) δ = 158.62, 158.28, 148.83, 140.71, 130.53, 129.83, 129.39, 128.80, 128.22, 128.12, 127.88, 126.90, 113.61, 113.23, 80.38, 69.68, 66.79, 55.52, 55.47, 20.35; LCMS (ESI) m/z: [M-H] calcd, 661.2; found, 660.2

Compound 20. 1*H*-Tetrazole (0.23 g, 16.4 mmol,0.8 eq) and 1-methylimidazole (0.085 mL, 1.0 mmol, 0.25 eq) were added to a solution of compound (**13c**) N-(1-((2R,4S)-5-((R)-1-(bis(4-methoxyphenyl)(phenyl)methoxy)ethyl)-4-hydroxytetrahydrofuran-2-yl)-5-methyl-2-oxo-1,2-dihydropyrimidin-4-yl)benzamide (15.0 g, 20.60 mmol) in DMF (51 mL) at room temperature.

3-bis(diisopropylamino)phosphanyloxypropanenitrile (2.06 mL, 6.49 mmol, 1.5 eq) was added drop wise and the reaction was stirred at room temperature for 3 hours. Water (5.0 mL) was added to quench the reaction. A 3:1 mixture of toluene/hexanes (80 mL) was added and the organic layer was washed (4x50 mL) with a 3:2 mixture of DMF/H_2O. The organics were then washed with saturated sodium bicarbonate solution, brine, dried over solid sodium sulfate and concentrated under reduced pressure to a white foam. Purification by flash chromatography (Silica gel, eluting with 40% ethyl acetate/hexanes + 1 % triethylamine) afforded the desired product (**14d**) as a white solid. 3.14 g, 84 % yield ^{1}H NMR (300MHz, CHLOROFORM-d) δ = 8.33 - 8.27 (m, 2H), 7.55 - 7.37 (m, 9H), 7.31 - 7.19 (m, 5H), 6.87 - 6.80 (m, 4H), 4.12 (d, *J*=7.0 Hz, 1H), 3.93 (br s, 1H), 3.78 (d, *J*=2.8 Hz, 9H), 2.69 - 2.44 (m, 3H), 2.04 (s, 2H), 1.63 (s, 4H), 1.32 - 1.13 (m, 14H), 1.05 - 0.89 (m, 3H) ^{31}P NMR (121MHz, CHLOROFORM-d) δ = 149.00 (s, 1P), 148.57 (s, 1P); LCMS (ESI) m/z: [M-H] calcd, 861.4; found, 860.4

Scheme S3. Synthesis of 5'-(S)-Methyl Cytidine Nucleosides.

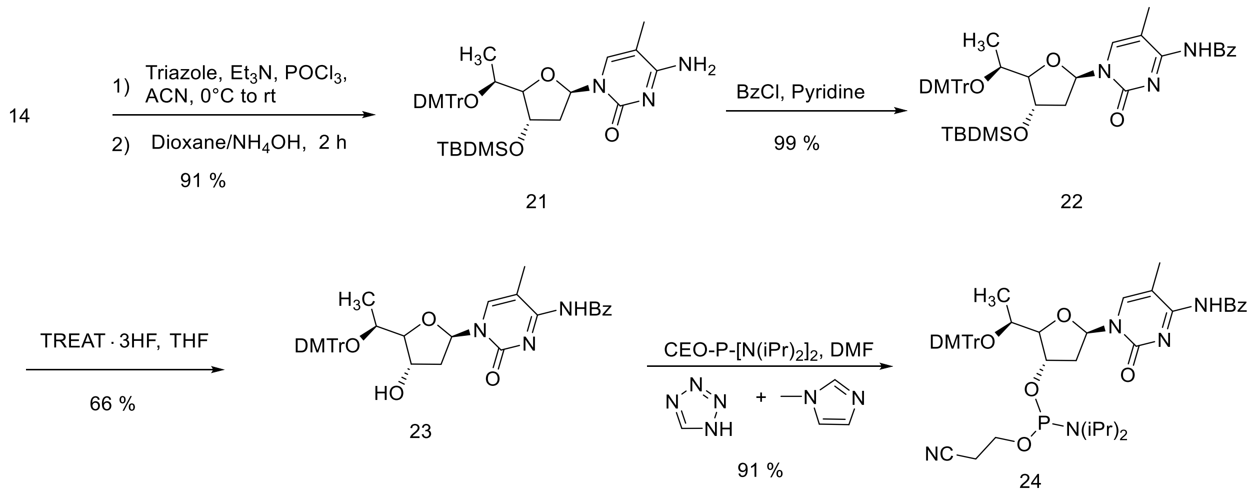

Compound 21. $POCl_3$ (4.53 mL, 49.5 mmol, 8 eq) was added dropwise to a suspension of 1,2,4-1*H*-Triazole (14.5 g, 210 mmol, 34 eq.) in acetonitrile (200 mL) and the reaction was stirred for 20 minutes at room temperature. The reaction was cooled in an ice bath and triethylamine (34.5

mL, 247 mmol, 40 eq.) was added. The ice bath was removed and the reaction was stirred at room temperature for another 30 minutes. The reaction was cooled in an ice bath and a solution of compound **(12c)** 1-((2R,4S)-5-((S)-1-(bis(4-methoxyphenyl)(phenyl)methoxy)ethyl)-4-((tert-butyldimethylsilyl)oxy)tetrahydrofuran-2-yl)-5-methylpyrimidine-2,4(1H,3H)-dione (4.16 g, 6.18 mmol) in acetonitrile (30 mL) was added dropwise to reaction. This was stirred at room temperature for 16 hours and concentrated under reduced pressure and diluted with ethyl acetate. The organic layer was washed with aqueous saturated sodium bicarbonate (2x), water, brine and concentrated to a yellow oil and used without any further purification. The crude material obtained above was suspended in dioxane/NH_4OH (30 mL/10 mL) solution and stirred at room temperature for 2 hours. TLC in EtOAc/hexanes (8/2) indicated reaction was completed. Solvent was concentrated under reduce pressure and remaining oil was diluted with ethyl acetate and the organic layer was washed with water, sat. NaHCO3, brine and dried over sodium sulfate, filtered and concentrated under reduce pressure to obtain a crude oil. The crude material was dissolved in purified by column chromatography (silica gel, 40-60% EtOAc/hexanes) to afford the desired product as a white solid. 3.77 g, 91 % yield. ^{1}H NMR (300MHz, CHLOROFORM-d) δ = 8.06 (s, 1H), 7.52 - 7.22 (m, 10H), 6.86 (dd, *J*=6.0, 8.1 Hz, 4H), 6.32 (t, *J*=6.1 Hz, 1H), 3.86 - 3.82 (m, 6H), 3.80 - 3.60 (m, 2H), 1.75 (s, 4H), 1.05 (d, *J*=6.3 Hz, 3H), 0.88 - 0.80 (m, 9H) ^{13}C NMR (75MHz, CHLOROFORM-d) δ = 165.53, 158.63, 158.60, 155.93, 146.08, 138.54, 136.76, 136.46, 130.56, 130.40, 128.40, 127.66, 126.92, 112.98, 101.21, 91.50, 86.70, 85.76, 71.77, 69.64, 55.25, 42.59, 25.71, 19.13, 17.76, 12.93, -4.41, -4.93; LCMS (ESI) m/z: [M-H] calcd, 671.3; found, 670.3

Compound 22. Compound **(13a)** 4-amino-1-((2R,4S)-5-((R)-1-(bis(4-methoxyphenyl)(phenyl)methoxy)ethyl)-4-((tert-butyldimethylsilyl)oxy)tetrahydrofuran-2-yl)-5-methylpyrimidin-2(1H)-one (3.77 g, 5.59 mmol) was dissolved in anhydrous pyridine (50 mL) and the reaction was cooled in an ice bath. Benzoyl chloride (0.71 mL, 6.51 mmol, 1.1 3q.) was added dropwise and the reaction was stirred at room temperature for 16 hours at which time TLC analysis in (EtOAc/hexanes (6/4)) indicated that the reaction was completed. The reaction was diluted with ethyl acetate and the organic layer was washed with water, sat. $NaHCO_3$, brine, dried over sodium sulfate, filtered and concentrated under reduce pressure to obtain a crude oil. The crude material was dissolved in DCM and purified by column chromatography (silica gel,

40-70% EtOAc/hexanes) afforded the desired product as a white solid. 3.35 g, 99 % yield. ^{1}H NMR (300MHz, CHLOROFORM-d) δ = 7.35 (br d, *J*=7.8 Hz, 5H), 7.27 - 7.14 (m, 5H), 7.13 - 7.02 (m, 3H), 6.73 (d, *J*=8.8 Hz, 4H), 3.69 (s, 6H), 1.98 (d, *J*=18.5 Hz, 3H), 1.31 - 1.11 (m, 3H), 0.87 - 0.76 (m, 7H), 0.71 (s, 2H), 0.03 - -0.05 (m, 5H), -0.11 (d, *J*=11.7 Hz, 1H) ^{13}C NMR (75MHz, CHLOROFORM-d) δ = 158.62, 147.36, 139.50, 132.48, 130.04, 129.92, 128.64, 128.35, 128.31, 128.14, 127.86, 127.80, 129.16, 127.39, 127.08, 113.17, 113.06, 111.93, 90.85, 87.41, 81.45, 72.64, 67.30, 55.26, 40.49, 25.75, 25.70, 25.67, 20.60, 17.95, 13.68; LCMS (ESI) m/z: [M-H] calcd, 775.3; found, 674.3

Compound 23. Triethylamine (1.95 mL, 14.0 mmol, 2.5 eq.) was added to a solution of compound **(14b)** N-(1-((2R,4S)-5-((S)-1-(bis(4-methoxyphenyl)(phenyl)methoxy)ethyl)-4-((tert-butyldimethylsilyl)oxy)tetrahydrofuran-2-yl)-5-methyl-2-oxo-1,2-dihydropyrimidin-4-yl)benzamide (4.35 g, 5.59 mmol) in tetrahydrofuran (50.0 mL). The reaction was cooled in an ice bath. Triethylamine trihydrofluoride (4.56 mL, 28.0 mmol, 5 eq.) was slowly added and the reaction was stirred for 16 hours at room temperature after which triethylamine (1.95 ml) was added to the reaction and the solvent was removed under reduced pressure. The crude material was dissolved in ethyl acetate and the organic layer was washed with water, saturated NaHCO3, brine, dried over Na_2SO_4, filtered and the solvent was concentrated under reduced pressure. Purification by flash chromatography (silica gel, 20-50 % EtOAc/hexanes) afforded the desired product as a white solid. 2.45 g, 66 % yield. 1H NMR (300MHz, DMSO-d6) δ = 13.02 (br s, 1H), 8.32 - 8.12 (m, 3H), 7.69 - 7.39 (m, 4H), 7.35 - 7.16 (m, 7H), 7.11 - 7.03 (m, 4H), 6.92 - 6.77(m, 5H), 6.24 - 6.15 (m, 2H), 5.25 (d, *J*=4.4 Hz, 1H), 5.10 (d, *J*=4.6 Hz, 1H), 4.38 - 4.07 (m, 1H), 4.03 - 3.76 (m, 1H), 3.70 - 3.53 (m, 2H), 3.33 (s, 4H), 2.48 - 2.10 (m, 2H), 2.07 - 1.94 (m, 3H), 1.90 (s, 1H), 1.83 (s, 1H) 13C NMR (75MHz, DMSO-d6) δ = 158.28, 148.83, 140.71, 132.97, 129.83, 129.38, 128.79, 128.12, 127.88, 126.90, 113.23, 91.23, 80.38, 71.29, 66.18, 55.47, 20.85; LCMS (ESI) m/z: [M-H] calcd, 661.2; found, 660.2

Compound 24. 1*H*-Tetrazole (0.22 g, 3.21 mmol,0.8 eq) and 1-Methylimidazole (0.080 mL, 1.0 mmol, 0.25 eq) were added to a solution of compound (**15c**) N-(1-((2R,4S)-5-((S)-1-(bis(4-methoxyphenyl)(phenyl)methoxy)ethyl)-4-hydroxytetrahydrofuran-2-yl)-5-methyl-2-oxo-1,2-

dihydropyrimidin-4-yl)benzamide (2.24 g, 4.0 mmol) in DMF (40 mL) at room temperature under an atmosphere of nitrogen. 3-bis(Diisopropylamino)phosphanyloxypropanenitrile (1.91 mL, 6.0 mmol, 1.5 eq) was then added drop wise and the reaction was stirred at room temperature for 3 hrs. Water (5.0 mL) was added to quench the reaction. A 3:1 mixture of toluene/hexanes (50 mL) was added and the organic layer was washed (4x50 mL) with a 3:2 mixture of DMF/H_2O. The organics were then washed with saturated sodium bicarbonate solution, brine, dried over solid sodium sulfate and concentrated under reduced pressure to a white foam. Purification by flash chromatography (Si, 50g col, 40-60% ethyl acetate/hexanes + 1 % triethylamine) afforded the desired product (**16d**) as a white solid.

2.76 g, 91 % yield. 1H NMR (300MHz, CHLOROFORM-d) δ = 7.55 - 7.33 (m, 9H), 7.26 (s, 6H), 6.84 (br d, *J*=4.4 Hz, 4H), 3.83 - 3.51 (m, 12H), 2.35 (s, 3H), 2.04 -1.93 (m, 3H), 1.22 - 1.11 (m, 14H), 1.05 (br d, *J*=6.8 Hz, 6H). 31P NMR (121MHz, CHLOROFORM-d) δ = 149.35 (s, 1P), 149.13 (s, 1P); LCMS (ESI) m/z: [M-H] calcd, 861.3; found, 860.3

Scheme S4. Synthesis of 5’-(R)-Methyl Adenosine Nucleosides.

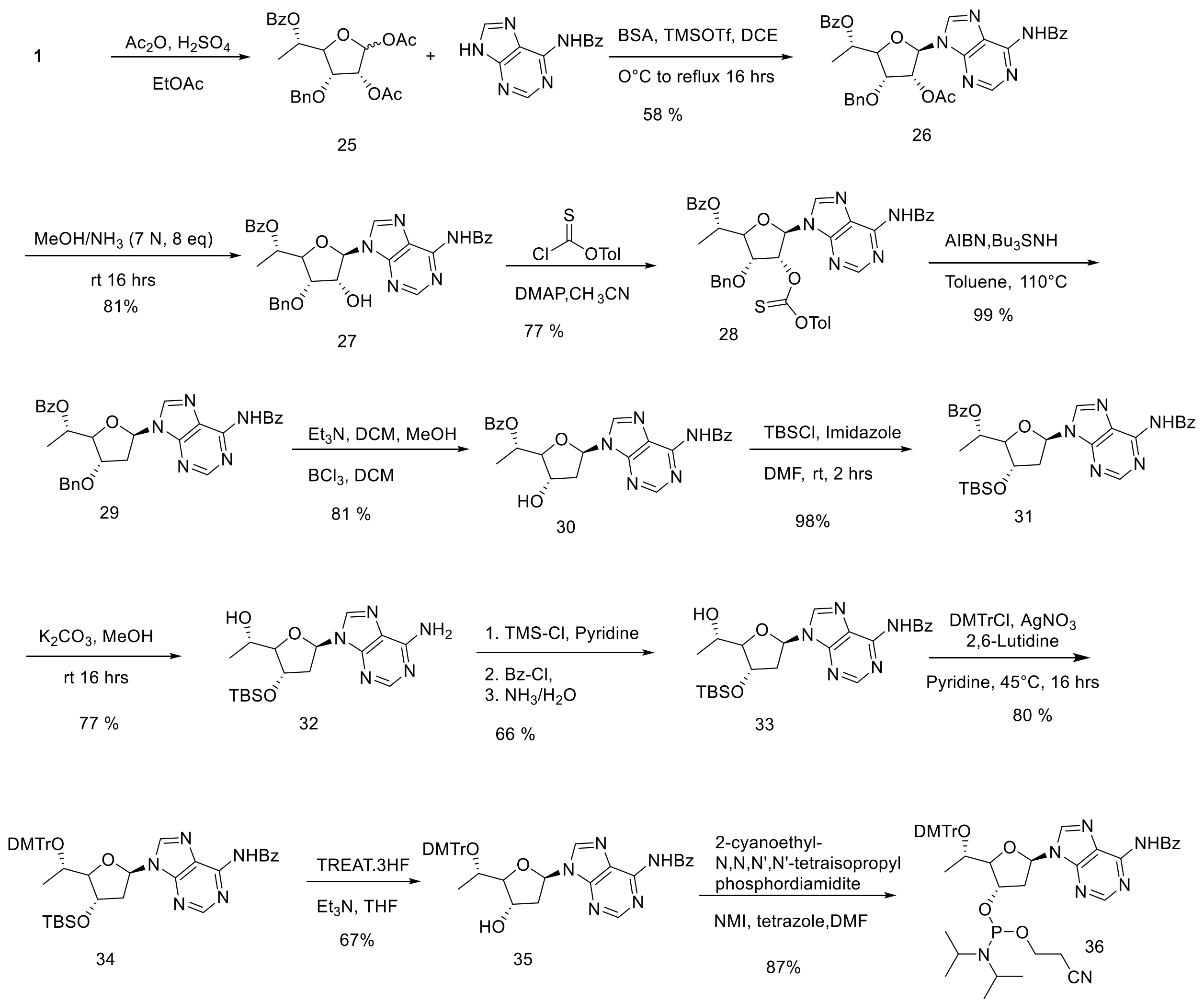

C**ompound 26.** *N*-(9H-purin-6-yl)benzamide (31.9 g, 133 mmol, 1.3 eq) and *N*,*O*-Bis(trimethylsilyl)acetamide (83.30 g, 100 mL, 410 mmol, 4.0 eq) were added to a solution of (3R,4R)-5-((S)-1-(benzoyloxy)ethyl)-4-(benzyloxy)tetrahydrofuran-2,3-diyl diacetate (2) (45.3 g, 102 mmol) in dichloroethane (600 mL). After heating at 80°C for 1 hour to obtain a clear solution, the reaction was cooled in an ice bath and trimethylsilyl trifluoromethanesulfonate (36.40 g, 164mmol, 1.6 eq) was added dropwise. The reaction was then warmed to 80 ^{o}C and stirred for 14 hours after which it was concentrated under reduced pressure to obtain a crude oil. The crude material was dissolved in ethyl acetate (500 mL) and the organic layer was washed with water, saturated sodium bicarbonate solution, brine, dried over Na_2SO_4 for 15 minutes, filtered and concentrated under reduced pressure to obtain a crude oil. Purification by flash chromatography (silica gel, 0-5% dichloromethane/methanol) afforded the desired product as a white solid. 35.18 g, 58 % yield. ^{1}H NMR (300MHz, CHLOROFORM-d) δ = 8.84 (s, 1H), 8.54

(s, 1H), 8.01 - 7.87 (m, 5H), 7.64 - 7.47 (m, 4H), 7.44 - 7.26 (m, 9H), 6.19 - 6.13 (m, 2H), 5.50 (dd, *J*=3.3, 6.7 Hz, 1H), 4.88 (t, *J*=4.9 Hz, 1H), 4.76 - 4.57 (m, 2H), 4.33 (dd, *J*=3.3, 5.4 Hz, 1H), 2.14 (s, 3H), 2.04 (s, 1H), 1.79 (s, 1H), 1.41 - 1.20 (m, 4H) ^{13}C NMR (75MHz, CHLOROFORM-d) δ = 170.00, 165.47, 164.40, 152.77, 151.22, 149.56, 142.08, 136.87, 133.57, 133.22, 132.82, 129.72, 129.53, 128.89, 128.58, 128.44, 128.38, 128.34, 127.83, 123.30, 87.05, 84.61, 75.46, 73.42, 73.10, 69.66, 20.72, 15.96. LCMS (ESI) m/z: [M-H] calcd, 671.3; found, 670.4; LCMS (ESI) m/z: [M+H] calcd, 621.2; found, 622.2

C**ompound 27.** Methanolic ammonia (7.00 M, 45 mL, 319 mmol, 6.0 eq) was added to a solution of (1S)-1-((3R,4R,5R)-4-acetoxy-5-(6-benzamido-9H-purin-9-yl)-3-(benzyloxy)tetrahydrofuran-2-yl)ethyl benzoate (3) (35 g, 56.30 mmol) in methanol (150.0 mL). The reaction was stirred in an ice bath for 16 hours and concentrated under reduced pressure while maintaining the bath temperature close to 0 °C. Purification by flash chromatography (silica gel, 0 - 3% dichloromethane/methanol) afforded the desired product as a white solid. 26.30 g, 81 % yield. ^{1}H NMR (300MHz, CHLOROFORM-d) δ = 9.24 (s, 1H), 8.54 (s, 1H), 7.99 - 7.87 (m, 5H), 7.58 - 7.27 (m, 12H), 5.97 (d, *J*=5.4 Hz, 1H), 5.48 (dd, *J*=4.5, 6.6 Hz, 1H), 5.04 (br d, *J*=5.6 Hz, 1H), 4.80 - 4.70 (m, 2H), 4.57 (dd, *J*=3.9, 5.3 Hz, 1H), 4.32 (t, *J*=4.2 Hz, 1H), 4.21 (d, *J*=6.3 Hz, 1H), 2.26 (s, 2H), 1.34 (d, *J*=6.5 Hz, 3H). ^{13}C NMR (75MHz, CHLOROFORM-d) δ = 165.51, 164.44, 152.58, 151.20, 149.57, 141.92, 136.50, 133.61, 133.31, 132.81, 129.65, 129.47, 128.88, 128.76, 128.57, 128.45, 128.35, 127.83, 123.19, 89.69, 84.88, 73.27, 72.90, 69.87, 16.34; LCMS (ESI) m/z: [M+H] calcd, 579.2; found, 579.1

C**ompound 28.** (1S)-1-((3S,4R,5R)-5-(6-benzamido-9H-purin-9-yl)-3-(benzyloxy)-4-hydroxytetrahydrofuran-2-yl)ethyl benzoate (4) (26.3 g, 45.50 mmol) and 4-Dimethylaminopyridine (11.10 g, 90.80 mmol, 2.0 eq) were dissolved in anhydrous acetonitrile (360 mL) followed by slow addition of *O*-4-methylphenyl chlorothioformate (10.20 g, 8.33 mL, 54.50 mmol, 1.20 eq). The reaction was stirred at room temperature for 2 hours. The solvent was removed under reduced pressure and the residue was partitioned between ethyl acetate and water. The aqueous layer was extracted with ethyl acetate and the combined organics were washed with

10% aqueous HCl, water, saturated sodium bicarbonate solution, and brine. The organic was dried over magnesium sulfate and concentrated. Purification by flash chromatography (350 g col, 0-3% dichloromethane/methanol) afforded the desired product as a white solid. 25.30 g, 77 % yield. ^{1}H NMR (300MHz, CHLOROFORM-d) δ = 9.02 (s, 1H), 8.39 (s, 1H), 8.03 - 7.94 (m, 5H), 7.61 - 7.34 (m, 11H), 7.26 - 7.16 (m, 2H), 6.91 (d, *J*=7.7 Hz, 2H), 6.58 (t, *J*=5.2 Hz, 1H), 6.35 (d, *J*=4.9 Hz, 1H), 5.53 (dd, *J*=3.0, 6.7 Hz, 1H), 5.11 (t, *J*=5.1 Hz, 1H), 4.83 - 4.72 (m, 2H), 4.42 (dd, *J*=3.0, 4.7 Hz, 1H), 4.11 (q, *J*=7.2 Hz, 1H), 2.36 (s, 3H), 2.04 (s, 1H), 1.38 - 1.22 (m, 4H). ^{13}C NMR (75MHz, CHLOROFORM-d) δ = 194.43, 165.52, 151.36, 151.24, 142.28, 136.84, 136.77, 133.56, 133.27, 132.83, 130.05, 129.68, 129.50, 128.64, 128.57, 128.42, 129.29 (dd, *J*=37.6, 99.6 Hz, 1C), 127.86, 123.40, 121.21, 120.50, 86.36, 85.22, 81.01, 74.81, 73.39, 69.68, 20.98, 16.13; LCMS (ESI) m/z: [M+H] calcd, 729.2; found, 730.3

C**ompound 29.** Azobisisobutyronitrile (AIBN) (0.72 g, 0.70 mL 4.38 mmol, 0.12 eq) and tributyltin hydride (26 g, 23.60 mL, 87.60 mmol, 2.5 eq) in toluene (30 mL) were added dropwise to a degassed (with nitrogen) solution of (1S)-1-((3R,4R,5R)-5-(6-benzamido-9H-purin-9-yl)-3-(benzyloxy)-4-((phenoxycarbonothioyl)oxy)tetrahydrofuran-2-yl)ethyl benzoate (5) (25 g, 35.10 mmol) in toluene (300 mL) and the solution was refluxed at 110°C for two hours. The solvents were concentrated under reduced pressure. Purification by flash chromatography (silica gel, 0-2% dichloromethane/methanol) afforded the desired product as a white solid. 19.5 g, 99 % yield. ^{1}H NMR (300MHz, CHLOROFORM-d) δ = 8.66 (s, 1H), 8.05 - 7.91 (m, 6H), 7.63 - 7.25 (m, 14H), 6.45 (dd, *J*=5.8, 8.1 Hz, 1H), 5.48 (dd, *J*=5.3, 6.5 Hz, 1H), 4.63 (s, 2H), 4.59 - 4.44 (m, 1H), 4.31 (dd, *J*=2.6, 5.2 Hz, 1H), 3.00 (ddd, *J*=6.0, 7.9, 13.7 Hz, 1H), 2.69 (ddd, *J*=2.3, 5.8, 13.5 Hz, 1H), 2.14 (s, 1H), 2.04 (s, 1H), 1.91 (br s, 3H), 1.38 (d, *J*=6.5 Hz, 4H), 1.26 (s, 1H), ^{13}C NMR (75MHz, CHLOROFORM-d) δ = 165.59, 141.45, 137.17, 133.28, 132.78, 129.82, 128.57, 128.08, 128.67 (dd, *J*=46.9, 77.1 Hz, 1C), 127.82, 123.40, 86.92, 85.22, 78.33, 71.50, 70.32, 37.25, 16.72; LCMS (ESI) m/z: [M+H] calcd, 563.2; found, 564.2

C**ompound 30.** Borontrichloride (BCl_3) 1.0 M in DCM (90.90 mL 90.9 mmol, 2.5 eq) was added slowly to a solution of (1S)-1-((3S,5R)-5-(6-benzamido-9H-purin-9-yl)-3-

(benzyloxy)tetrahydrofuran-2-yl)ethyl benzoate (6) (20.50 g, 36.40 mmol) in anhydrous dichloromethane (350 mL) at -78°C. After the addition, the reaction was stirred at -78°C for 30 minutes. Quenched reaction by adding slowly a mixture solution of Et_3N/MeOH/DCM/ (180 mL/180 mL/180 mL) while keeping temperature at -70°C continue stir reaction for one hour. This solution was slowly added to a cold saturated $NaHCO_3$ solution and stir for 20 minutes. The aqueous layer was removed and the organic layer was washed for second time with saturated $NaHCO_3$ solution, dried over Na_2SO_4 and filtered. The solvent was concentrated under reduced pressure. Purification by flash chromatography (silica gel, 0-5% methanol/dichloromethane) afforded the desired product as a white solid. 14.80 g, 81 % yield. ^{1}H NMR (300MHz, CHLOROFORM-d) δ = 8.96 (s, 1H), 8.68 (s, 1H), 8.06 - 7.94 (m, 6H), 7.64 - 7.37 (m, 7H), 6.47 (t, *J*=6.7 Hz, 1H), 5.48 (dd, *J*=5.1, 6.5 Hz, 1H), 4.92 (td, *J*=3.2, 6.1 Hz, 1H), 4.19 - 4.08 (m, 2H), 3.16 - 2.83 (m, 4H), 2.60 (ddd, *J*=3.3, 6.1, 13.6 Hz, 1H), 2.05 (s, 1H), 1.47 - 1.37 (m, 7H), 1.26 (t, *J*=7.2 Hz, 2H), ^{13}C NMR (75MHz, CHLOROFORM-d) δ = 165.69, 152.65, 141.45, 133.30, 132.81, 129.81, 129.50, 129.25, 128.87, 128.49, 128.45, 127.87, 127.74, 88.87, 84.77, 71.71, 70.51, 45.84, 40.21, 16.63, 8.62; LCMS (ESI) m/z: [M+H] calcd, 473.2; found, 473.1

C**ompound 31.** (1S)-1-((3S,5R)-5-(6-benzamido-9H-purin-9-yl)-3-hydroxytetrahydrofuran-2-yl)ethyl benzoate (**7**) (14.0 g, 31.20 mmol) was dissolved in anhydrous DMF (300 mL) under nitrogen. Imidazole (8.28 g, 122 mmol, 2.20 eq) was added and the reaction was cooled in an ice bath and tert-butyl-chloro-dimethyl-silane (11.0 g, 73.0 mmol) was slowly added as a solution in anhydrous DMF (20 ml). The reaction was stirred for 16 hours at room temperature and the reaction was slowly poured onto 400 mL of ice. Then hexanes (800 mL) were added. The mixture was stirred and let stand for 90 minutes to precipitate. The solvents were then decanted. The white solid was washed with water and hexanes two more times. The white solid was then co-evaporated with toluene under reduced pressure at 60°C twice to give the desired product as a white foam 16.80 g, 92 % yield. ^{1}H NMR (300MHz, CHLOROFORM-d) δ = 8.99 (br s, 1H), 8.58 (s, 1H), 7.95 - 7.81 (m, 6H), 7.51 - 7.26 (m, 7H), 7.14 (s, 1H), 6.31 (dd, *J*=5.9, 7.8 Hz, 1H), 5.34 (dd, *J*=5.6, 6.4 Hz, 1H), 4.67 - 4.63 (m, 1H), 4.00 (dd, *J*=2.5, 5.4 Hz, 1H), 2.92 (s, 1H), 2.03 - 1.67 (m, 3H), 1.26 (d, *J*=6.5 Hz, 4H), 0.83 - 0.73 (m, 14H), ^{13}C NMR (75MHz, CHLOROFORM-d) δ = 165.67, 164.57, 152.64, 151.42, 149.53, 141.62, 133.68, 133.26, 132.76,

129.81, 129.50, 128.85, 128.43, 127.88, 123.39, 89.62, 85.15, 72.24, 70.24, 40.12, 25.72, 25.65, 18.13, 17.99, 17.91, 16.60; LCMS (ESI) m/z: [M+H] calcd, 587.3; found, 588.3

C**ompound 32.** (1S)-1-((3S,5R)-5-(6-benzamido-9H-purin-9-yl)-3-((tert-butyldimethylsilyl)oxy)tetrahydrofuran-2-yl)ethyl benzoate (8) (16.0 g, 27.20 mmol) was dissolved in methanol (200 mL). K_2CO_3 (15 g, 109 mmol, 4 eq.) was added, to the reaction and stirred for 16 hours after which the solid was filtered and rinsed with DCM/MeOH (8/2) the filtrate was concentrated under reduced pressure to half of volume. The solution was let sit at room temperature and product crystalized out from solution within 30 minutes. The solid was collected and rinsed with water (2x10 mL), suspend the solid in diethyl ether (50 mL) stir mixture for 30 minutes, filtered the solid to obtained 9.11 g, 88 % yield. ^{1}H NMR (300MHz, CHLOROFORM-d) δ = 8.32 (s, 1H), 7.87 (s, 1H), 6.91 (s, 1H), 6.25 (dd, *J*=5.2, 9.9 Hz, 1H), 5.77 (br s, 2H), 4.68 (d, *J*=4.6 Hz, 1H), 4.10 (d, *J*=6.7 Hz, 1H), 4.03 - 3.96 (m, 1H), 3.04 (ddd, *J*=5.0, 10.0, 13.0 Hz, 1H), 2.15 (dd, *J*=5.4, 13.0 Hz, 1H), 1.75 (br s, 7H), 1.35 - 1.21 (m, 5H), 0.98 - 0.87 (m, 11H), ^{13}C NMR (75MHz, CHLOROFORM-d) δ = 155.89, 152.34, 148.65, 140.39, 121.36, 94.15, 87.60, 71.66, 67.64, 41.34, 25.74, 18.62, 17.89; LCMS (ESI) m/z: [M+H] calcd, 379.2; found, 380.2

C**ompound 33.** ((1S)-1-((3S,5R)-5-(6-amino-9H-purin-9-yl)-3-((tert-butyldimethylsilyl)oxy)tetrahydrofuran-2-yl)ethan-1-ol (9) (9.0 g, 24.0 mmol) was dissolved in pyridine (100 mL) under nitrogen. Cooled solution with ice bath and added trimethylsilyl chloride (13.50 mL, 108 mmol, 5 eq.) dropwise. Removed the ice bath and let reaction stir for 1 hr at room temperature. Cooled reaction again with ice bath, added dropwise benzoyl chloride (13.9 mL, 120 mmol, 5 eq.) after the addition, continue stir reaction for 16 hours by letting reaction warm up slowly to room temperature. The reaction was cooled in an ice bath and water (60 ml) was added dropwise while keeping temperature below 7°C. Continued to stir reaction at room temperature for 1 hour. Cooled reaction, NH_4OH (60) mL) was added dropwise and continue stir reaction for another 30 minutes. Evaporated most of the aqueous NH_4OH at room temperature. The remaining solution was diluted with EtOAc and the organics were washed with water 100 (ml). The aqueous layer was removed, and organic layer was washed with

saturated $NaHCO_3$, brine, dried over Na_2SO_4 filtered and filtrate was concentrated under reduced pressure. Purification by flash chromatography (silica gel, 0-5% methanol/dichloromethane) afforded the desired product as a white solid. 10.5 g, 90 % yield. ^{1}H NMR (300MHz, CHLOROFORM-d) δ = 9.11 (br s, 1H), 8.66 - 8.63 (m, 1H), 7.97 - 7.88 (m, 3H), 7.69 (s, 1H), 7.68 - 7.66 (m, 1H), 7.51 - 7.28 (m, 6H), 7.13 (s, 1H), 6.19 (dd, *J*=5.2, 9.9 Hz, 2H), 4.56 (d, *J*=4.7 Hz, 1H), 3.99 (dd, *J*=1.5, 6.8 Hz, 1H), 3.92 - 3.87 (m, 1H), 2.91 (ddd, *J*=5.0, 9.9, 13.0 Hz, 1H), 2.07 (dd, *J*=5.3, 13.0 Hz, 1H), 1.94 - 1.58 (m, 3H), 1.16 (d, *J*=6.8 Hz, 3H), 0.83 - 0.76 (m, 10H), ^{13}C NMR (75MHz, CHLOROFORM-d) δ = 152.02, 150.68, 150.36, 142.70, 133.48, 133.28, 132.93, 132.05, 128.90, 128.64, 127.96, 127.35, 124.66, 94.12, 87.65, 71.53, 67.67, 41.43, 25.75, 18.54, 17.90; LCMS (ESI) m/z: [M+H] calcd, 483.2; found, 484.2

C**ompound 34.** N-(9-((2R,4S)-4-((tert-butyldimethylsilyl)oxy)-5-((S)-1-hydroxyethyl)tetrahydrofuran-2-yl)-9H-purin-6-yl)benzamid (10) (6 g, 12.40 mmol) was co-evaporated with toluene (2x40 mL) under reduced pressure at 60°C. Dissolved material into dry pyridine (100 mL) added 2,6-lutidine (3.60 mL, 30.9 mmol 4 eq.). To this was added DMTr-Cl (16.8 g, 30.9 mmol 4 eq.) and silver nitrate (3.50 g, 20.6 mmol 3.8 eq.) and the reaction was heated to 45°C for 16 hours. The next day, after cooling to 0°C and ethyl acetate was added, followed by water. The aqueous layer was removed and the organic layer was washed with saturated sodium bicarbonate solution, brine, dried over Na_2SO_3 filter and concentrated to a crude yellow oil under reduced pressure. Purification by flash chromatography (silica gel, 0-60% ethyl acetate/hexanes) gave the desired product as yellow foam 7.76 g, 80 % yield. ^{1}H NMR (300MHz, CHLOROFORM-d) δ = 8.95 (s, 1H), 8.73 (s, 1H), 8.01 (d, *J*=7.2 Hz, 2H), 7.89 (s, 1H), 7.81 (d, *J*=7.5 Hz, 1H), 7.64 - 7.34 (m, 11H), 7.32 - 7.27 (m, 1H), 7.24 - 7.06 (m, 3H), 6.82 (s, 2H), 6.80 - 6.78 (m, 2H), 6.37 (dd, *J*=6.0, 7.9 Hz, 1H), 4.61 - 4.56 (m, 1H), 4.12 (q, *J*=7.1 Hz, 1H), 3.97 (dd, *J*=2.6, 5.7 Hz, 1H), 3.82 - 3.74 (m, 7H), 3.57 (t, *J*=6.0 Hz, 1H), 2.04 (s, 2H), 1.72 (s, 1H), 1.26 (t, *J*=7.1 Hz, 3H), 0.95 - 0.85 (m, 11H), ^{13}C NMR (75MHz, CHLOROFORM-d) δ = 158.64, 158.53, 139.49, 136.82, 132.76, 132.05, 130.40, 128.94, 127.91, 129.14 (t, *J*=96.9 Hz, 1C), 128.27 (dd, *J*=16.5, 74.6 Hz, 1C), 128.11 (dd, *J*=32.9, 81.2 Hz, 1C), 127.08, 126.84, 113.18, 113.00, 112.98, 91.30, 86.49, 72.35, 71.54, 55.26, 55.25, 39.79, 25.74, 17.89, 17.71; LCMS (ESI) m/z: [M-H] calcd, 785.4; found, 784.3

C**ompound 35.** Triethylamine (3.46 mL, mmol, 2.5 eq) was added to a solution of 9-((2R,4S)-5-((S)-1-(bis(4-methoxyphenyl)(phenyl)methoxy)ethyl)-4-((tert-butyldimethylsilyl)oxy)tetrahydrofuran-2-yl)-2-(isobutylamino)-1,9-dihydro-6H-purin-6-one (11) (7.80 g, 9.92 mmol) in dry THF (100 mL). The reaction was cooled in an ice bath and triethylamine trihydrofluoride (8.09 mL, 49.6 mmol, 5 eq.) was added. The reaction was warmed up slowly to room temperature and stirred for another 16 hours.

The reaction was quenched by adding triethylamine (3.5 mL) and concentrated under reduced pressure to obtain a crude yellow oil. Purification by flash chromatography (silica gel, 0-3% methanol/dichloromethane + 1% Et_3N) gave the product as a white solid 4.38 g, 67 % yield. ^1H NMR (300MHz, CHLOROFORM-d) δ = 9.09 (br s, 1H), 8.70 (s, 1H), 8.02 (d, *J*=7.2 Hz, 2H), 7.81 (s, 1H), 7.82 (d, *J*=8.1 Hz, 2H), 7.63 - 7.35 (m, 11H), 7.30 - 7.17 (m, 4H), 6.85 - 6.78 (m, 4H), 6.38 (t, *J*=6.5 Hz, 1H), 4.54 - 4.47 (m, 1H), 4.12 (q, *J*=7.2 Hz, 1H), 3.88 (dd, *J*=3.9, 6.7 Hz, 1H), 3.78 (d, *J*=1.4 Hz, 6H), 3.59 (d, *J*=7.3 Hz, 1H), 3.48 (t, *J*=6.4 Hz, 1H), 3.09 (q, *J*=7.3 Hz, 1H), 2.58 (td, *J*=6.6, 13.4 Hz, 2H), 2.43 (ddd, *J*=4.4, 6.4, 13.6 Hz, 1H), 2.07 - 2.03 (m, 2H), 1.48 - 1.36 (m, 2H), 1.26 (t, *J*=7.1 Hz, 2H), 0.98 (d, *J*=6.1 Hz, 3H), ^{13}C NMR (75MHz, CHLOROFORM-d) δ = 158.62, 142.57, 139.49, 132.94, 132.06, 130.30, 130.24, 129.15, 128.90, 128.85, 127.97, 127.82, 127.79, 127.36, 127.85 (t, *J*=59.0 Hz, 1C), 113.16, 93.14, 87.31, 81.45, 70.88, 67.65, 55.26, 45.85, 40.93, 18.38, 8.66; LCMS (ESI) m/z: [M-H] calcd, 671.3; found, 670.3

C**ompound 36.** Benzamide **35** (2.38 g, 3.54 mmol) was dissolved in dry DMF (30 mL) under an atmosphere of nitrogen. To this was added 1H-tetrazole (0.196 g, 2.83 mmol, 0.8 eq) and 1-methylimidazole (72 µL, 0.88 mmol, 0.25 eq), followed by dropwise addition of 3-bis(diisopropylamino)phosphanyloxypropanenitrile (1.69 mL, 5.31 mmol, 1.5 eq). This was stirred at room temperature for 90 minutes. Water (1 mL) was added to quench the reaction. The reaction was diluted with a mixture of toluene/hexanes (3:1) The organics were washed four times with a mixture of DMF/H_2O (3:2). The organics were then washed with saturated sodium bicarbonate solution, brine, dried over sodium sulfate and concentrated to a white foam. Purification by flash chromatography (silica gel, 50 g col, 0-5% methanol/dichloromethane + 1 % triethyl amine) gave the desired product as a white solid 2.70

g, 87 % yield. ^{1}H NMR (300MHz, CHLOROFORM-d) δ = 9.06 - 8.90 (m, 1H), 8.72 (s, 1H), 8.01 (d, *J*=7.3 Hz, 2H), 7.90 - 7.85 (m, 1H), 7.63 - 7.35 (m, 9H), 7.30 - 7.17 (m, 4H), 6.84 - 6.77 (m, 4H), 6.40 (t, *J*=6.9 Hz, 1H), 4.84 (ddd, *J*=2.6, 5.5, 8.0 Hz, 1H), 4.17 - 4.07 (m, 2H), 3.78 (d, *J*=1.4 Hz, 7H), 2.68 - 2.45 (m, 4H), 2.04 (s, 2H), 1.93 - 1.78 (m, 1H), 1.33 - 1.14 (m, 15H), 0.92 - 0.84 (m, 3H), ^{31}P NMR (121MHz, CHLOROFORM-d) δ = 149.05 (s, 1P), 148.54 (s, 1P), LCMS (ESI) m/z: [M-H] calcd, 871.4; found, 870.3

Scheme S5. Synthesis of 5'-(S) Methyl Adenosine Nucleosides.

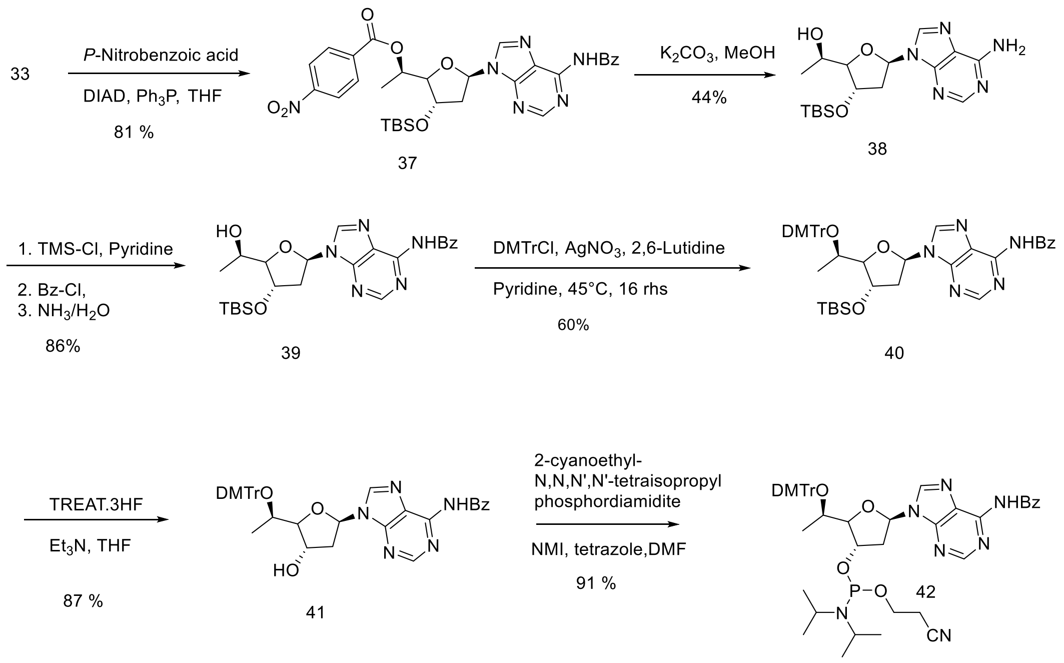

C**ompound 37.** Benzamide **33** (7.60 g, 15.7 mmol), triphenylphosphine (16.5 g, 4eq, 69.9 mmol) and p-nitrobenzoic acid (10.5 g, 4 eq, 62.9 mmol) were dissolved in anhydrous THF (50 mL). Diisopropyl azodicarboxylate (DIAD) (12.4 g, 3.9 eq, 12.2 mL, 61.4 mmol) was added dropwise and the reaction was stirred for 4 hours at room temperature. The solvent was removed under reduced pressure and the residue was partitioned between ethyl acetate and water. The aqueous layer was washed twice with ethyl acetate and the organic layers were combined and washed with saturate $NaHCO_3$, brine, dried over Na_2SO_4, filtered and the solvent was removed under reduced pressure to give a crude oil. Purification by flash chromatography (silica gel, 0-

50% ethyl acetate/hexanes) afforded the desired product as a white solid. 8.0 g, 81 % yield. ^{1}H NMR (300MHz, CHLOROFORM-d) δ = 8.98 (s, 1H), 8.59 - 8.56 (m, 1H), 8.15 - 8.10 (m, 3H), 7.97 - 7.90 (m, 4H), 7.74 - 7.66 (m, 1H), 7.62 - 7.28 (m, 84H), 7.15 (s, 2H), 6.31 (t, *J*=6.3 Hz, 1H), 5.34 (dd, *J*=4.6, 6.5 Hz, 1H), 4.58 (q, *J*=5.4 Hz, 1H), 3.97 (t, *J*=4.7 Hz, 1H), 2.83 (td, *J*=5.9, 13.0 Hz, 1H), 2.41 (ddd, *J*=5.5, 6.6, 13.2 Hz, 1H), 1.93 (br d, *J*=0.9 Hz, 1H), 1.88 - 1.68 (m, 8H). ^{13}C NMR (75MHz, CHLOROFORM-d) δ = 164.07, 152.50, 150.62, 141.98, 133.20, 131.98, 131.82, 130.59, 132.17, 128.44, 132.04, 131.94, 128.60, 88.69, 84.64, 71.84, 70.80, 40.56, 25.70, 17.89, 17.19. LCMS (ESI) m/z: [M+H] calcd, 632.2; found, 633.2

C**ompound 38.** 4-Nitrobenzoate **37** (8.0 g, 12.10 mmol) was dissolved in methanol (150 mL). K_2CO_3 (6.67 g, 4 eq, 48.4 mmol) was added to the reaction and it was stirred for 16 hours. The solids was removed by filtration and rinsed with DCM/MeOH (8/2). The filtrate was reduced under reduced pressure to half volume. The solution was let sit at room temperature and the product crystalized out from solution within 30 minutes. The product was collected, suspended in diethyl ether (50 mL) and stirred for 30 minutes, filtered to obtain the product as a solid 2.0 g, 44 % yield. ^{1}H NMR (300MHz, CHLOROFORM-d) δ = 8.21 (s, 1H), 7.75 (s, 1H), 7.16 (s, 1H), 6.21 - 6.07 (m, 2H), 5.81 (br s, 2H), 4.53 (d, *J*=4.7 Hz, 1H), 3.87 - 3.75 (m, 2H), 2.93 (ddd, *J*=5.0, 9.7, 13.0 Hz, 1H), 2.09 - 1.91 (m, 1H), 1.13 (d, *J*=6.5 Hz, 3H), 0.86 - 0.77 (m, 9H). ^{13}C NMR (75MHz, CHLOROFORM-d) δ = 155.90, 152.32, 148.73, 140.34, 121.35, 93.08, 87.83, 75.16, 68.23, 40.57, 25.81, 20.38, 18.02. LCMS (ESI) m/z: [M+H] calcd, 379.2; found, 380.2

C**ompound 39.** Alcohol **38** (9.0 g, 24.0 mmol) was dissolved in pyridine (80 mL) and the reaction was cooled in an ice bath. Trimethylsilyl chloride (3.34 mL, 26.3 mmol, 5 eq.) was added dropwise after which the ice bath was removed, and the reaction was stirred at room temperature for 1 hour. The reaction was cooled again with an ice bath and benzoyl chloride (3.0 mL, 26.3 mmol, 5 eq.) was added dropwise. After the addition, the reaction was warmed to room temperature and continued to stir for 16 hours. The reaction was cooled reaction with an ice bath, and water (63 ml) was added dropwise while keeping temperature below 7°C. The reaction was stirred at room temperature for 1 hour before cooling with an ice bath and quenched by dropwise

addition of aqueous NH_4OH (15 mL) and further stirring for another 30 minutes. The volume of water was reduced under pressure and diluted with EtOAc. The organic layer was washed with water 100 (ml), saturated aqueous $NaHCO_3$, brine, dried over Na_2SO_4, filtered and concentrated under reduced pressure. Purification by flash chromatography (silica gel, 1-5% methanol/dichloromethane) afforded the desired product as a white solid. 2.2 g, 86 % yield. ^{1}H NMR (300MHz, CHLOROFORM-d) δ = 9.03 (s, 1H), 8.67 - 8.63 (m, 1H), 8.00 - 7.88 (m, 3H), 7.71 (s, 1H), 7.68 (s, 1H), 7.58 - 7.29 (m, 10H), 7.15 (s, 1H), 6.25 (dd, *J*=5.3, 9.5 Hz, 1H), 5.36 (d, *J*=10.9 Hz, 1H), 4.53 (d, *J*=4.9 Hz, 1H), 3.88 - 3.77 (m, 2H), 2.92 (ddd, *J*=5.1, 9.5, 13.0 Hz, 1H), 2.10 (ddd, *J*=0.8, 5.4, 13.0 Hz, 1H), 1.79 (s, 1H), 1.16 - 1.10 (m, 3H), 0.84 - 0.78 (m, 10H). ^{13}C NMR (75MHz, CHLOROFORM-d) δ = 164.61, 152.04, 150.79, 150.26, 142.65, 133.49, 133.31, 133.16, 132.93, 132.17, 132.03, 131.99, 131.96, 128.91, 128.63, 128.61, 128.45, 127.94, 127.36, 124.64, 93.02, 87.77, 77.73, 74.88, 68.17, 40.67, 25.81, 20.4; LCMS (ESI) m/z: [M+H] calcd, 483.2; found, 484.2

C**ompound 40.** Alcohol **39** (2.2 g, 4.5 mmol) was co-evaporated with toluene (40 mL) under reduced pressure at 60°C three times before being dissolved into dry pyridine (100 mL) and 2,6-lutidine (2.88 mL, 24.8 mmol, 4eq.). To this was added DMT-Cl (8.41 g, 24.80 mmol, 4 eq.) and silver nitrate (1.2 g, 20.6 mmol, 2.5 eq.) and the reaction was heated to 45°C for 16 hours. After cooling to 0°C, ethyl acetate was added, followed by water. The aqueous layer was removed, and the organic layer was washed with saturated sodium bicarbonate solution, brine, dried over Na_2SO_3 filter and concentrated under reduced pressure to a crude yellow oil. Purification product by flash chromatography (silica gel, 100g col, 0-60% ethyl acetate/hexanes) gave the desired product as yellow foam 2.90 g, 83 % yield. ^{1}H NMR (300MHz, CHLOROFORM-d) δ = 9.00 (br s, 1H), 8.80 (s, 1H), 8.29 (s, 1H), 8.07 - 8.00 (m, 2H), 7.64 - 7.15 (m, 15H), 6.81 - 6.73 (m, 4H), 6.41 (dd, *J*=5.2, 8.7 Hz, 1H), 4.51 - 4.46 (m, 1H), 4.12 (q, *J*=7.2 Hz, 1H), 3.88 (dd, *J*=2.0, 4.0 Hz, 1H), 3.78 (d, *J*=2.6 Hz, 6H), 2.77 (ddd, *J*=5.7, 8.6, 13.1 Hz, 1H), 2.41 (ddd, *J*=1.7, 5.3, 13.0 Hz, 1H), 2.04 (s, 1H), 1.81 - 1.68 (m, 1H), 1.26 (t, *J*=7.2 Hz, 2H), 0.94 - 0.84 (m, 13H). ^{13}C NMR (75MHz, CHLOROFORM-d) δ = 164.55, 158.53, 158.47, 145.90, 141.48, 136.71, 136.50, 133.73, 132.78, 130.56, 130.43, 129.15, 128.89, 128.44, 127.86, 127.61, 126.80, 113.18, 112.94, 91.54, 86.58, 84.58, 72.50, 69.64, 55.21, 41.57, 25.81, 25.75, 17.86, 17.84; LCMS (ESI) m/z: [M-H] calcd, 785.4; found, 784.3

Compound 41. Triethylamine (1.29 mL, 9.22 mmol, 2.5 eq.) was added to a solution of benzamide **40** (2.90 g, 2.91 mmol) in THF (30 mL). The reaction was cooled with an ice bath under an atmosphere of nitrogen. Triethylamine trihydrofluoride (3.1 mL, 18.4 mmol, 5eq.) was added slowly and then the reaction warmed to room temperature and stirred for 16 hours. Triethylamine (1.3 mL) was added, and the solvents were removed to give a pale yellow solid. Purification by flash chromatography (silica gel, 100g col, 0-3% methanol/dichloromethane + 1 % Et_3N) gave the product as a white solid 2.0 g, 87 % yield. ^{1}H NMR (300MHz, CHLOROFORM-d) δ = 9.09 (br s, 1H), 8.77 (s, 1H), 8.12 (s, 1H), 8.02 (d, *J*=7.3 Hz, 2H), 7.63 - 7.43 (m, 5H), 7.39 - 7.20 (m, 8H), 6.81 (dd, *J*=2.8, 8.8 Hz, 4H), 6.30 (t, *J*=6.5 Hz, 1H), 4.80 (br d, *J*=6.4 Hz, 1H), 4.12 (q, *J*=7.2 Hz, 1H), 3.98 - 3.86 (m, 1H), 3.79 (s, 6H), 3.55 (t, *J*=4.7 Hz, 1H), 3.09 (q, *J*=7.3 Hz, 1H), 2.78 (br d, *J*=6.7 Hz, 1H), 2.48 (br dd, *J*=6.0, 13.3 Hz, 2H), 2.27 (s, 1H), 2.04 (s, 2H), 1.47 - 1.36 (m, 3H), 1.26 (t, *J*=7.2 Hz, 3H), 0.93 (d, *J*=6.3 Hz, 3H). ^{13}C NMR (75MHz, CHLOROFORM-d) δ = 158.65, 147.34, 139.48, 129.26, 129.15, 127.86, 127.78, 127.74, 127.09, 113.18, 81.47, 55.27, 8.63; LCMS (ESI) m/z: [M-H] calcd, 671.3; found, 670.3

Compound 42. Alcohol **41** (2.0 g, 3.20 mmol) was dissolved in dry DMF (30 mL) under an atmosphere of nitrogen. To this was added 1H-tetrazole (0.18 g, 2.5 mmol, 0.8 eq.) and 1-methylimidazole (0.063 mL, 0.80 mmol, 0.25 eq.), followed by dropwise addition of 3-bis(diisopropylamino)phosphanyloxypropanenitrile (1.52 mL, 4.80 mmol, 1.5 eq). This was stirred at room temperature for 90 minutes. Water (1 mL) was added to quench the reaction. The reaction was diluted with a mixture of toluene/hexanes (3:1) The organics were washed four times with a mixture of DMF/H_2O (3:2). The organics were then washed with saturated sodium bicarbonate solution, brine, dried over sodium sulfate and concentrated to a white foam. Purification by flash chromatography (silica gel, 50 g col, 0-5% methanol/dichloromethane + 1 % triethyl amine) gave the desired product as a white solid 2.55 g, 91 % yield. ^{1}H NMR (300MHz, CHLOROFORM-d) δ = 8.99 - 8.74 (m, 2H), 8.26 - 8.19 (m, 1H), 8.06 - 7.99 (m, 2H), 7.63 - 7.33 (m, 10H), 7.29 - 7.17 (m, 4H), 6.83 - 6.72 (m, 4H), 6.44 - 6.36 (m, 1H), 4.78 (br d, *J*=2.3 Hz, 1H), 4.20 - 4.00 (m, 2H), 3.89 - 3.59 (m, 12H), 2.81 (s, 2H), 2.72 - 2.58 (m, 2H), 2.40 (t, *J*=6.4 Hz, 1H), 2.04 (s, 2H), 1.43 (s, 1H), 1.31 - 1.13 (m, 19H). ^{31}P

NMR (121MHz, CHLOROFORM-d) δ = 149.52 (s, 1P), 149.02 (s, 1P), LCMS (ESI) m/z: [M-H] calcd, 871.4; found, 870.3

Scheme S6. Synthesis of (R)-5'-Methyl Guanosine Nucleosides.

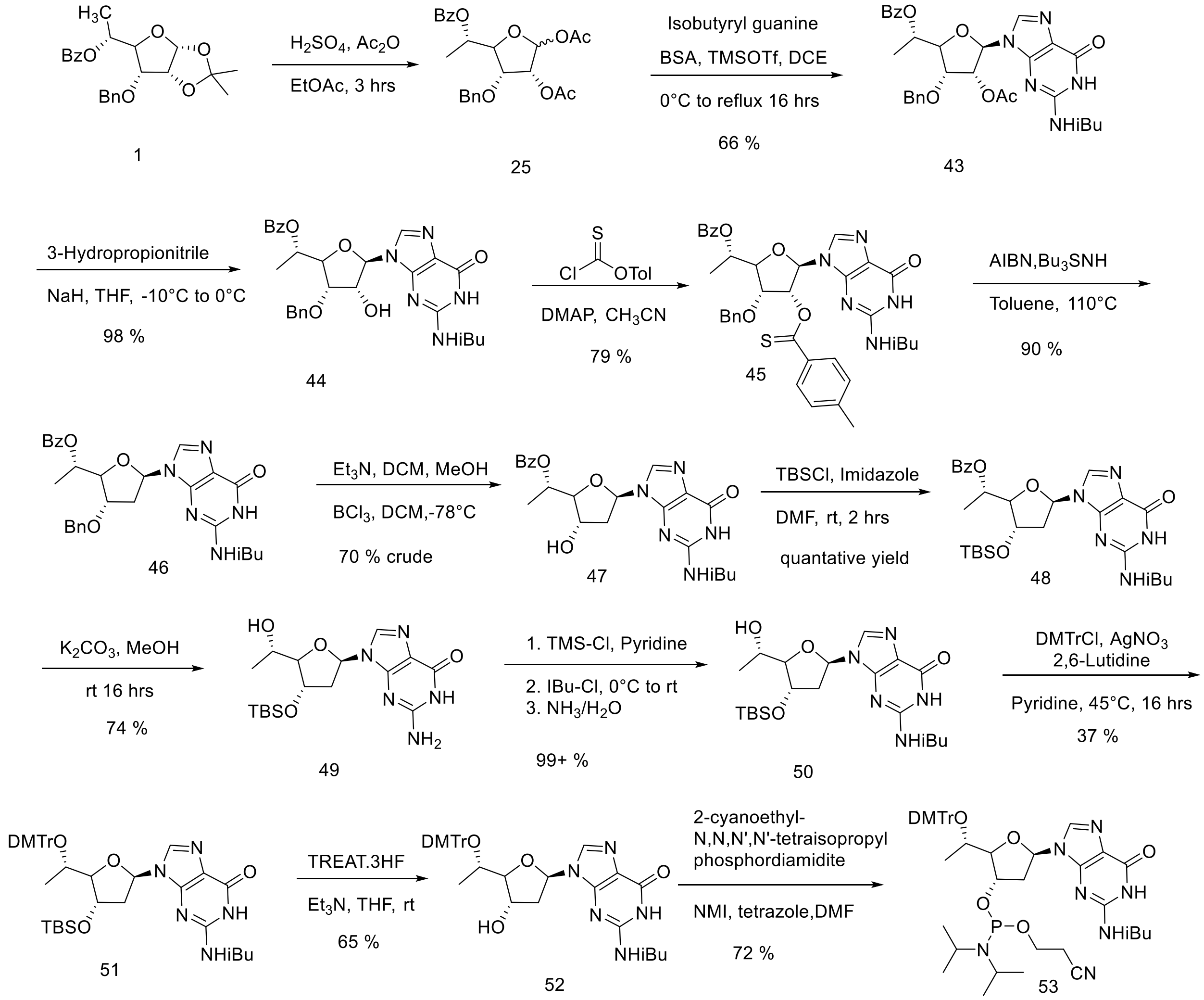

C**ompound 43.** 2-(iso-Butylamino)-1,9-dihydro-6H-purin-6-one (33.8 g, 153 mmol, 1.3 eq.) and N,O-Bis(trimethylsilyl)acetamide (115 mL, 410 mmol, 4.0 eq.) were added to a solution of diacetate **25** (52 g, 118 mmol) in dichloroethane (600 mL). After heating at 80°C for 1 hour a clear solution was obtained, this was then cooled with ice bath. Trimethylsilyl trifluoromethanesulfonate (41.8g, 180 mmol, 1.6 eq.) was added and continued to stir overnight at 80°C. The reaction was concentrated under reduced pressure to obtain a crude oil. The crude

material was dissolved in ethyl acetate (500 mL) and washed with water, saturated sodium bicarbonate solution, brine. The organics were dried over Na_2SO_4 for 15 minutes, filtered and concentrated under reduced pressure to obtain a crude oil. Purification by flash chromatography (silica gel, 350g col, 0-5% dichloromethane/methanol) afforded the desired product as a white solid. 47 g, 66 % yield. ^{1}H NMR (300MHz, DMSO-d_6) δ = 12.07 (s, 1H), 11.57 (s, 1H), 8.09 - 8.05 (m, 1H), 7.99 - 7.93 (m, 2H), 7.73 - 7.64 (m, 1H), 7.57 - 7.48 (m, 2H), 7.39 - 7.28 (m, 5H), 6.10 (d, *J*=5.0 Hz, 1H), 5.74 (t, *J*=5.3 Hz, 1H), 5.36 (dd, *J*=4.7, 6.5 Hz, 1H), 4.74 - 4.66 (m, 2H), 4.63 - 4.54 (m, 1H), 4.30 (t, *J*=4.8 Hz, 1H), 3.42 - 3.32 (m, 1H), 2.82 - 2.70 (m, 1H), 2.08 (s, 3H), 1.33 (d, *J*=6.5 Hz, 3H), 1.18 - 1.07 (m, 7H). ^{13}C NMR (75MHz, CHLOROFORM-d) δ = 179.03, 169.97, 167.20, 155.43, 147.77, 147.11, 138.43, 136.55, 133.89, 129.87, 129.51, 128.78, 128.36, 128.09, 128.06, 122.81, 89.05, 85.37, 75.28, 73.28, 70.54, 36.51, 20.70, 19.08, 18.88, 18.47; LCMS (ESI) m/z: [M+H] calcd, 589.3; found, 590.2

C**ompound 44.** To a three neck round flask, with a mechanical stirrer, was added anhydrous THF (250 mL) and 3-hydroxyproprionitrile (4.09 mL, 59.9 mmol, 1.4 eq) under an atmosphere of nitrogen. NaH (60 % mineral oil) (2.46 g, 64.2 mmol, 1.5 eq.) was added portion-wise at room temperature and stirred for 1hour. Acetate **43** (18.3, 31.0 mmol) in anhydrous THF (100 mL) was added to the reaction using a cannula keeping reaction between -10 to -1 °C. After addition the reaction was stirred for 2 hours. The reaction was quenched by adding slow addition of water (50 mL), keeping temperature below 0°C. Stirring was continued for 30 minutes and the reaction was transferred to a separatory funnel. The aqueous layer was extracted with EtOAc and the combined organics were washed with saturated $NaHCO_3$, brine, dried organic over Na_2SO_3, filtered and concentrated under reduced pressure to obtain a crude oil. Purification by flash chromatography (silica gel, 200g col, 0-3% dichloromethane/methanol) afforded the desired product as a white solid. 23 g, 98 % yield. ^{1}H NMR (300MHz, CHLOROFORM-d) δ = 12.09 (s, 1H), 9.96 (s, 1H), 8.02 - 7.87 (m, 2H), 7.66 - 7.38 (m, 4H), 7.33 - 7.14 (m, 6H), 6.15 - 5.94 (m, 1H), 5.86 (d, *J*=4.4 Hz, 1H), 4.92 (q, *J*=4.7 Hz, 1H), 4.76 - 4.47 (m, 4H), 4.40 - 4.27 (m, 1H), 4.27 - 4.13 (m, 1H), 3.90 (br d, *J*=1.0 Hz, 1H), 2.87 (quin, *J*=6.9 Hz, 1H), 2.61 (t, *J*=6.3 Hz, 1H), 1.89 (s, 3H), 1.47 - 1.23 (m, 10H). ^{13}C NMR (75MHz, CHLOROFORM-d) δ = 179.08, 166.98, 155.47, 147.44, 147.29, 139.00, 136.81, 133.74, 129.78, 129.58, 128.70, 128.65, 128.53, 128.30,

128.16, 128.09, 121.97, 91.60, 85.49, 78.66, 73.57, 72.99, 70.45, 57.97, 36.40, 30.32, 21.52, 18.95, 18.90, 18.09; LCMS (ESI) m/z: [M+H] calcd, 547.2; found, 548.2

C**ompound 45.** Alcohol **44** (20.3 g, 36.1 mmol) and 4-dimethylaminopyridine (8.81 g, 72.2 mmol, 2.0 eq) were dissolved in anhydrous acetonitrile (360 mL), followed by slow addition of O-4-methylphenyl chlorothioformate (6.62 mL, 43.3 mmol, 1.2 eq). The reaction was stirred at room temperature for 2 hours. The solvent was removed under reduced pressure and the residue was partitioned between ethyl acetate and water. The aqueous layer was extracted with ethyl acetate and the combined organics were washed with 10% HCl (aq.), water, saturated sodium bicarbonate solution, water and brine. The organics were dried over magnesium sulfate for 15 minutes, filtered and concentrated under reduced pressure to obtain a crude oil. Purification by flash chromatography (200 g col, 0-3% dichloromethane/methanol) afforded the desired product as a white solid. 20.3 g, 79 % yield. ^{1}H NMR (300MHz, CHLOROFORM-d) δ = 12.10 (s, 1H), 9.80 (s, 1H), 8.08 - 8.03 (m, 2H), 7.68 - 7.60 (m, 2H), 7.46 (t, *J*=7.5 Hz, 2H), 7.27 - 7.15 (m, 7H), 7.03 (d, *J*=6.5 Hz, 2H), 6.90 (d, *J*=7.7 Hz, 2H), 6.21 - 6.11 (m, 2H), 5.90 (dd, *J*=2.8, 5.4 Hz, 1H), 5.29 (t, *J*=5.6 Hz, 1H), 4.47 - 4.30 (m, 3H), 2.85 (quin, *J*=6.9 Hz, 1H), 2.36 (s, 3H), 1.69 (s, 5H), 1.49 - 1.25 (m, 10H). ^{13}C NMR (75MHz, CHLOROFORM-d) δ = 194.50, 179.00, 167.29, 155.40, 151.21, 147.82, 147.18, 138.42, 136.84, 136.43, 133.87, 130.21, 129.87, 129.48, 128.75, 128.41, 128.18, 122.66, 121.15, 88.45, 85.35, 83.17, 73.51, 70.61, 36.50, 20.96, 19.06, 18.88, 18.54; LCMS (ESI) m/z: [M+H] calcd, 681.3; found, 682.3

C**ompound 46.** Azobisisobutyronitrile (AIBN) (0.58 g, 3.5 mmol, 0.12 eq) and tributyltin hydride (19.3 mL, 71.7 mmol, 2.5 eq) in toluene (30 mL) was added dropwise to a degassed (with nitrogen) solution of 4-methylbenzothioate **45** (20 g, 28.7 mmol) in toluene (250 mL) at reflux. After addition the reaction was stirred for 1 hour and the solvent was removed under reduced pressure. Purification by flash chromatography (silica gel, 220g col, 0-5% dichloromethane/methanol) afforded the desired product as a white solid. 14 g, 90 % yield. ^{1}H NMR (300MHz, CHLOROFORM-d) δ = 12.04 (br s, 1H), 9.65 (s, 1H), 8.02 - 7.99 (m, 1H), 7.98 (s, 1H), 7.69 - 7.60 (m, 2H), 7.55 - 7.42 (m, 2H), 7.30 - 7.14 (m, 6H), 7.02 (d, *J*=8.1 Hz, 1H), 6.81 - 6.75 (m, 1H), 6.33 - 6.15 (m, 2H), 4.57 (td, *J*=2.9, 6.0 Hz, 1H), 4.48 (s, 2H), 4.25 (dd,

J=2.6, 9.2 Hz, 1H), 3.02 - 2.80 (m, 2H), 2.53 (ddd, *J*=3.1, 7.2, 14.0 Hz, 1H), 2.26 (s, 1H), 1.54 (s, 1H), 1.45 - 1.25 (m, 10H), 0.86 (s, 1H). ^{13}C NMR (75MHz, CHLOROFORM-d) δ = 179.07, 166.99, 155.62, 153.86, 147.41, 147.20, 138.66, 137.14, 133.75, 129.94, 129.70, 129.65, 129.35, 128.71, 128.47, 127.95, 127.79, 122.69, 115.23, 87.50, 86.57, 79.30, 71.55, 70.33, 37.01, 36.45, 20.47, 19.04, 18.83, 17.97; LCMS (ESI) m/z: [M+H] calcd, 531,3; found, 532.2

C**ompound 47.** Boron trichloride (BCl_3) 1.0 M in DCM (65.5 mL, 65.5 mmol, 2.5 eq) added slowly to a solution of benzoate **46** (18.6 g, 34.20 mmol) in anhydrous dichloromethane (350 mL) at -78°C under nitrogen. After the addition the reaction was stirred at -78°C for 30 minutes and then quenched by slowly adding a mixture of Et_3N/MeOH/DCM (180 mL/180 mL/180 mL), keeping temperature at -70°C. This was then stirred for one hour. The reaction was slowly added to a cold solution of saturated aqueous $NaHCO_3$ and stirred for 20 minutes. The aqueous layer was discarded, and the organics were washed a second time with saturated aqueous $NaHCO_3$ solution. The organic layer was dried over Na_2SO_4, filtered and concentrated under reduced pressure. Purification by flash chromatography (silica gel, 350g col, 0-5% methanol/dichloromethane) afforded the desired product as a white solid. 11 g crude, 70 % yield. Note: product mixt with boron salt hard to remove, product was taking to the next step as crude. ^{1}H NMR (300MHz, CHLOROFORM-d) δ = 12.08 (br s, 1H), 9.78 (s, 1H), 8.07 - 7.98 (m, 3H), 7.70 (s, 1H), 7.61 (br d, *J*=7.3 Hz, 1H), 7.55 - 7.42 (m, 3H), 7.27 (d, *J*=0.6 Hz, 3H), 6.27 - 6.13 (m, 2H), 5.03 - 4.97 (m, 1H), 4.11 (dd, *J*=3.1, 8.6 Hz, 1H), 3.01 - 2.82 (m, 4H), 1.88 - 1.50 (m, 8H), 1.50 - 1.19 (m, 12H). ^{13}C NMR (75MHz, CHLOROFORM-d) δ = 179.20, 166.96, 155.65, 147.45, 147.26, 138.74, 129.71, 129.66, 128.69, 122.57, 89.60, 86.10, 77.81, 72.49, 70.75, 39.66, 36.45, 19.05, 18.85, 17.91; LCMS (ESI) m/z: [M+H] calcd, 441.2; found, 442.2

C**ompound 48.** Alcohol **47** (1.0 g,24.2 mmol) was dissolved in anhydrous DMF (250 mL) under nitrogen. Imidazole (3.62 g, 53.0 mmol, 2.20 eq) was added and the solution was cooled with an ice bath. tert-Butyl-chloro-dimethyl-silane (6.12 g, 40.6 mmol) in anhydrous DMF (20 mL) was slowly added and the reaction was stirred for 16 hours. The reaction was slowly poured onto 400 mL of ice and stirred. The aqueous layer was extracted with EtOAc (2x100 mL). The combined

organics were washed with saturated NaHCO3 solution, brine, dried over, filtered and concentrated under reduce pressure. Purification by flash chromatography (silica gel, 220g col, 0-5% methanol/dichloromethane) afforded the desired product as a white solid. 13 g, 100 % yield crude. Without any further purification the crude material was taking to the next step, LCMS (ESI) m/z: [M+H] calcd, 555.3; found, 556.2

C**ompound 49.** Benzoate **48** (16.0 g, 27.20 mmol) was dissolved in methanol (200 mL). K_2CO_3 (13.4 g, 96.90 mmol, 4 eq.) was added and the reaction was stirred for 16 hours. The solvent was concentrated under reduced pressure to obtain a white solid. The solid was suspended in water (50 ml), filtered, and washed with diethyl ether (50 mL) to obtain the desired product. 7.13 g, 74 % yield. ^{1}H NMR (300MHz, DMSO-d_6) δ = 10.79 (br s, 1H), 7.92 (s, 1H), 6.60 (s, 2H), 6.13 - 6.06 (m, 1H), 5.04 (d, *J*=5.0 Hz, 1H), 4.58 (d, *J*=4.7 Hz, 1H), 3.73 - 3.53 (m, 2H), 3.34 (s, 1H), 1.06 (d, *J*=6.3 Hz, 3H), 0.92 - 0.83 (m, 11H), 0.13 - 0.08 (m, 7H). ^{13}C NMR (75MHz, METHANOL-d_4) δ = 155.21, 152.27, 138.54, 101.42, 93.94, 86.44, 73.54, 73.42, 72.06, 68.88, 62.27, 50.98, 41.86, 26.26, 19.47, 18.78; LCMS (ESI) m/z: [M+H] calcd, 395.2; found, 396.2

C**ompound 50.** Alcohol **49** (3.50 g, 8.85 mmol) was dissolved in pyridine (100 mL) under an atmosphere of nitrogen. The reaction was cooled with an ice bath, trimethylsilyl chloride (7.86 mL, 62 mmol, 7 eq.) was added dropwise. The ice bath was removed, and the reaction was stirred for 1 hour at room temperature. The reaction was cooled with an ice bath, isobutyl chloride (5.71 mL, 58.40 mmol, 6 eq.) was added dropwise stirred for 16 hours while warming up to room temperature. The reaction was cooled reaction with ice bath and quenched by dropwise addition of water (60 mL), keeping temperature below 5°C. The reaction was stirred at room temperature for 1 hour, cooled with an ice bath, followed by dropwise addition of aqueous NH_4OH (20 mL) and stirred reaction for a further 30 minutes. The reaction was concentrated under reduced, diluted with EtOAc and the organics were washed with water (100 ml), saturated $NaHCO_3$, brine, dried over Na_2SO_4 and concentrated under reduced pressure. Purification by flash chromatography (silica gel, 100g col, 0-5% methanol/dichloromethane) afforded the desired product as a white solid. 4.0 g, 98+% yield. ^{1}H NMR (300MHz, CHLOROFORM-d) δ = 12.08 (s, 1H), 9.69 (s, 1H), 8.07 - 7.90 (m, 3H), 7.72 - 7.57 (m, 2H), 7.56 - 7.38 (m, 3H), 7.34 -

7.14 (m, 8H), 6.34 - 6.13 (m, 2H), 4.80 - 4.52 (m, 2H), 4.48 (s, 2H), 4.25 (dd, *J*=2.4, 9.2 Hz, 2H), 3.14 - 2.92 (m, 1H), 2.91 - 2.78 (m, 2H), 2.10 (s, 1H), 1.89 (s, 3H). ^{13}C NMR (75MHz, CHLOROFORM-d) δ = 178.89, 166.96, 155.66, 147.34, 147.18, 138.68, 137.16, 133.72, 129.70, 129.67, 129.53, 128.75, 128.69, 128.57, 128.52, 128.46, 127.98, 127.94, 127.79, 122.75, 87.50, 86.56, 79.32, 71.55, 70.35, 37.04, 36.45, 19.03, 18.82, 17.97; LCMS (ESI) m/z: [M+H] calcd, 451.3; found, 452.3

C**ompound 51.** Alcohol **50** (2.5 g, 5.37 mmol) was co-evaporated with toluene (2x40 mL) under reduced pressure at 60°C and dissolved into dry pyridine (50 mL) added 2,6-lutidine (2.49 mL, 21.5 mmol, 4 eq.). To this was added DMT-Cl (7.28 g, 21.5 mmol 4 eq.) and silver nitrate (3.50 g, 20.6 mmol 3.8 eq.), and the reaction was heated to 45°C for 16 hours. The reaction was cooled down with an ice bath and ethyl acetate was added, followed by water. The aqueous layer was discarded and the organics were washed with saturated sodium bicarbonate solution, brine, dried organic over Na_2SO_3, filtered and concentrated under reduced pressure to a crude yellow oil. Purification product by flash chromatography (silica gel, 100g col, 0-60% ethyl acetate/hexanes (1L), 0-3% methanol/dichloromethane) gave the desired product as yellow foam 1.5 g, 37% yield. ^{1}H NMR (300MHz, CHLOROFORM-d) δ = 12.07 (s, 1H), 8.26 (br s, 1H), 7.52 - 7.33 (m, 8H), 7.32 - 7.16 (m, 6H), 6.86 - 6.74 (m, 5H), 6.14 (t, *J*=7.1 Hz, 1H), 4.56 (br d, *J*=2.0 Hz, 1H), 4.12 (q, *J*=7.1 Hz, 1H), 3.90 (dd, *J*=2.2, 5.5 Hz, 1H), 3.82 - 3.74 (m, 8H), 3.45 - 3.36 (m, 1H), 2.53 - 2.41 (m, 1H), 2.16 - 2.03 (m, 3H), 1.72 (s, 1H), 1.31 - 1.12 (m, 10H), 0.95 - 0.72 (m, 14H), 0.14 - -0.01 (m, 8H). ^{13}C NMR (75MHz, CHLOROFORM-d) δ = 177.90, 158.65, 158.62, 158.59, 155.67, 148.25, 147.19, 146.05, 136.75, 136.65, 136.52, 130.47, 130.43, 130.31, 129.15, 128.41, 128.36, 127.86, 127.78, 127.70, 126.92, 121.59, 113.18, 113.02, 113.01, 91.56, 86.51, 86.49, 83.19, 72.15, 69.79, 55.30, 55.28, 55.25, 39.84, 36.52, 25.53, 25.73, 19.06, 19.04, 18.96, 18.91, 18.22, 18.12, 17.86; LCMS (ESI) m/z: [M-H] calcd, 753.4; found, 752.3

C**ompound 52.** Triethylamine (0.65 mL, 4.69 mmol, 2.5 eq) was added to a solution of compound **51**(1.4 g, 1.88 mmol) in dry THF (20 mL). The reaction was cooled in an ice bath. Triethylamine trihydrofluoride (1.53 mL, 9.38 mmol, 5 eq.) was added dropwise and continued to stir reaction for 16 hours, letting the reaction warm up slowly to room temperature.

The reaction was quenched by addition of 0.65 mL of triethylamine, and then concentrated under reduced pressure to obtained a crude yellow oil. Purification by flash chromatography (silica gel, 5g col, 0-3% methanol/dichloromethane + 1% Et_3N) gave the product as a white solid 0.89 g, 65 % yield. Used as in in the next step, LCMS (ESI) m/z: [M-H] calcd, 639.3; found, 638.3

C**ompound 53.** Alcohol **52** (0.80 g, 1.22 mmol) was dissolved in dry DMF (5 mL) under an atmosphere of nitrogen. To this was added 1H-tetrazole (67 mg, 0.97 mmol, 0.8 eq) and 1-methylimidazole (4.3 µL, 0.30 mmol, 0.25 eq), followed by dropwise addition of 3-bis(diisopropylamino)phosphanyloxypropanenitrile (0.56 mL, 1.84 mmol, 1.5 eq). This was stirred at room temperature for 90 minutes. Water (1 mL) was added to quench the reaction. The reaction was diluted with a mixture of toluene/hexanes (3:1). The organics were washed four times with a mixture of DMF/H_2O (3:2). The organics were then washed with saturated sodium bicarbonate solution, brine, dried over sodium sulfate and concentrated to a white foam. Purification by flash chromatography (silica gel, 5 g col, ethyl acetate + 1 % triethyl amine) gave the desired product as a white solid 0.75 g, 72 % yield. ^{1}H NMR (300MHz, CHLOROFORM-d) δ = 12.06 - 11.88 (m, 1H), 8.87 (br s, 1H), 7.53 - 7.15 (m, 12H), 6.85 - 6.74 (m, 4H), 6.17 - 6.04 (m, 1H), 4.86 - 4.75 (m, 1H), 4.18 - 4.00 (m, 2H), 3.91 - 3.53 (m, 12H), 3.47 - 3.38 (m, 1H), 2.79 - 2.67 (m, 2H), 2.63 - 2.35 (m, 3H), 2.14 - 2.03 (m, 2H), 1.93 - 1.60 (m, 2H), 1.37 - 1.09 (m, 24H), 0.97 - 0.75 (m, 3H). ^{31}P NMR (121MHz, CHLOROFORM-d) δ = 149.08 (s, 1P), 147.39 (s, 1P), LCMS (ESI) m/z: [M-H] calcd, 839.4; found, 838.3

Scheme S7. Synthesis of (S)-5’-Methyl Guanosine Nucleosides.

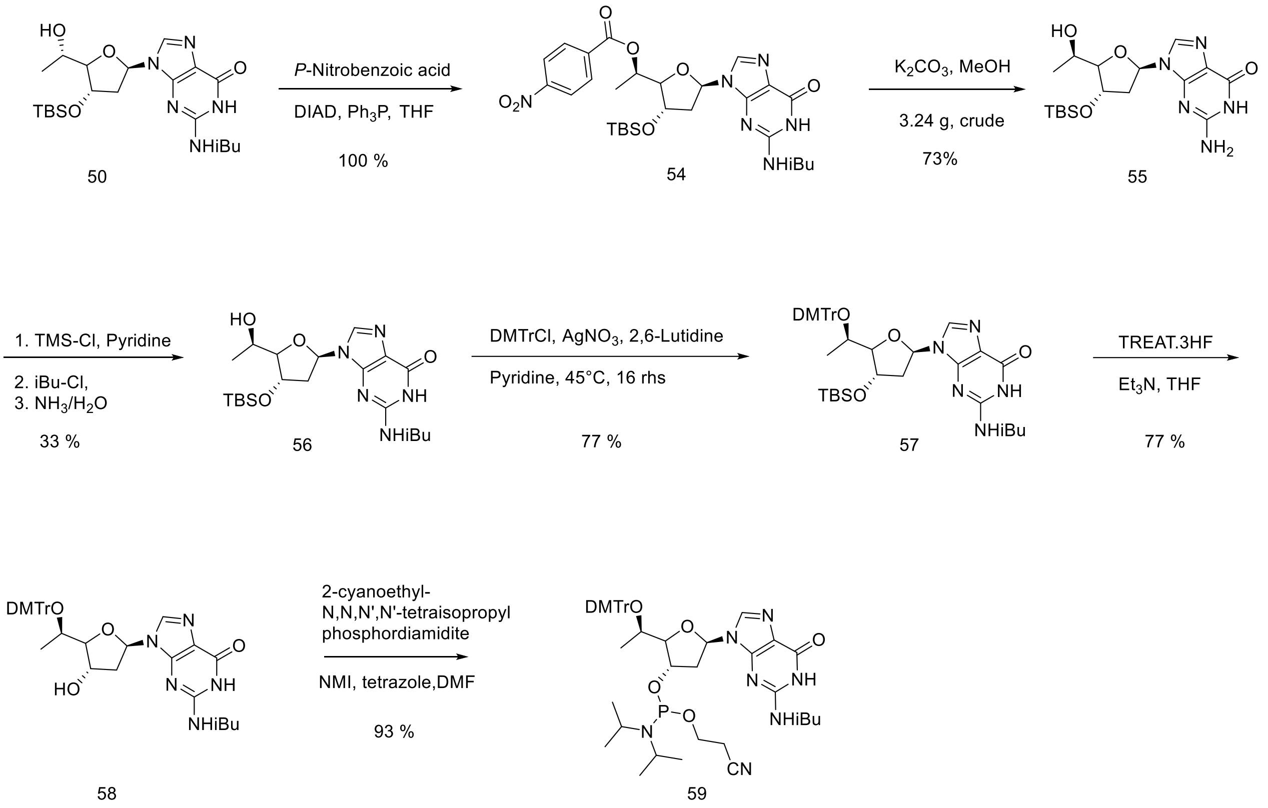

C**ompound 54.** Alcohol **50** (5.27 g, 11.30 mmol,) was dissolved in anhydrous THF (50 mL) under nitrogen. Triphenylphosphine (11.9 g, 45.3 mmol, 4 eq.) and p-nitrobenzoic acid (7.57 g, 45.3 mmol, 4 eq.) were added, followed by dropwise addition of diisopropyl azodicarboxylate (8.77 mL, 44.2 mmol, 3.9 eq.). The reaction was stirred reaction for 4 hours. The solvent was removed under reduced pressure and the residue was partitioned between ethyl acetate and water. The aqueous layer was extracted with ethyl acetate (2x) and the combined organics were washed with saturate $NaHCO_3$ solution, brine, dried over Na_2SO_4, filtered and concentrated under reduced pressure to a crude oil. Purification by flash chromatography (silica gel, 220g col, 0-2% dichloromethane/methanol) afforded the desired product as a white solid. 7.8 g, 100 % yield. Note: Product contaminated with a small amount of Ph_3P. Product used in the next step without further purification. ^{1}H NMR (300MHz, CHLOROFORM-d) δ = 12.01 (br s, 1H), 9.19 (br s, 1H), 8.30 - 8.16 (m, 1H), 8.12 - 8.06 (m, 1H), 7.78 - 7.43 (m, 16H), 7.27 (s, 1H), 6.23 - 6.09 (m, 1H), 5.55 (t, *J*=6.5 Hz, 1H), 5.29 - 5.21 (m, 1H), 4.49 - 4.44 (m, 1H), 4.31 - 4.26 (m, 1H), 4.14 - 4.06 (m, 1H), 2.89 - 2.69 (m, 1H), 2.66 - 2.56 (m, 1H), 2.28 (ddd, *J*=3.6, 6.1, 13.3 Hz, 1H), 2.09

- 2.02 (m, 1H), 1.48 (d, *J*=6.4 Hz, 1H), 1.38 - 1.12 (m, 12H), 0.94 - 0.84 (m, 8H); LCMS (ESI) m/z: [M+H] calcd, 600.3; found, 601.3

C**ompound 55.** 4-Nitrobenzoate **54** (6.90 g, 11.20 mmol) was dissolved in methanol (100 mL), followed by addition of K_2CO_3 (3.88 g, 28.1mmol, 2.5). The reaction was stirred for 16 hours at room temperature. The solvent was removed under reduced pressure to obtained a white solid. The solid was suspended in water (50 ml), filtered and rinsed with diethyl ether (50 mL) to obtained 3.24 g, 73 % yield. Used as is in the next step, LCMS (ESI) m/z: [M+H] calcd, 395.2; found, 396.2

C**ompound 56.** Alcohol **55** (3.34 g, 8.40 mmol) was dissolved in pyridine (60 mL) under an atmosphere of nitrogen and cooled with an ice bath. Trimethylsilyl chloride (7.50 mL, 59 mmol, 7 eq.) was added dropwise. The ice bath was removed and the reaction was stirred for 1 hr at room temperature. The reaction again cooled with an ice bath, followed by dropwise addition of isobutyryl chloride (5.44 mL, 55.7 mmol, 7 eq.) and stirred for a further16 hours letting reaction warm up slowly to room temperature. The reaction was cooled with an ice bath, and water (30 mL) was added dropwise while keeping temperature below 5°C. Stirring was continued at room temperature for 1 hour. The reaction was cooled in an ice bath, followed by dropwise addition of aqueous NH_4OH (20 mL) and stirred for another 30 minutes. Evaporation of most of the aqueous NH_4OH at room temperature was achieved under reduce pressure. The remaining solution was diluted with EtOAc and washed with water 100 (ml). The aqueous layer was discarded, and the organics were washed with saturated $NaHCO_3$, brine, dried over Na_2SO_4, filtered and concentrated under reduced pressure. Purification by flash chromatography (silica gel, 100g col, 0-5% Methanol/Dichloromethane) afforded the desired product as a white solid. 1.30 g, 33 % yield. ^{1}H NMR (300MHz, CHLOROFORM-d) δ = 12.14 (br dd, *J*=2.2, 3.5 Hz, 1H), 8.78 (s, 1H), 7.75 - 7.72 (m, 1H), 6.19 (dd, *J*=5.4, 9.4 Hz, 1H), 4.55 (d, *J*=5.4 Hz, 1H), 3.97 - 3.89 (m, 2H), 2.85 - 2.62 (m, 2H), 2.20 (ddd, *J*=1.4, 5.6, 13.1 Hz, 1H), 1.99 - 1.65 (m, 10H), 1.60 (s, 1H), 1.38 - 1.13 (m, 12H), 0.96 - 0.85 (m, 9H), 0.15 - 0.01 (m, 7H). ^{13}C NMR (75MHz, CHLOROFORM-d) δ = 147.50, 138.65, 92.03, 86.96, 74.52, 68.36, 40.79, 36.36, 25.75, 25.69, 25.65, 20.89, 18.91, 17.99; LCMS (ESI) m/z: [M+H] calcd, 451.3; found, 452.3

Compound 57. Alcohol **56** (1.20 g, 2.58 mmol) was co-evaporated with toluene (2x40 mL) under reduced pressure at 60°C. This was then dissolved into dry pyridine (50 mL) and 2,6-lutidine (1.50 mL, 10.30 mmol, 4 eq.) was added. DMT-Cl (3.49 g, 10.3 mmol 4 eq.) and silver nitrate (1.7 g, 10 mmol 3.8 eq.) were then added and the reaction was heated to 45°C for 16 hours. The reaction was cooled in an ice bath and ethyl acetate (50 mL) was added, followed by water (50 mL) and stirred for 10 minutes. The aqueous layer was removed, and the organics were washed with saturated sodium bicarbonate solution, brine, dried organic over Na_2SO_3, filtered and concentrated to a crude yellow oil under reduced pressure. Purification product by flash chromatography (silica gel, 100g col, 0-60% ethyl acetate/hexanes (1L), 0-3% methanol/dichloromethane) afforded the desired product as yellow foam 1.5 g, 37% yield. ^{1}H NMR (300MHz, CHLOROFORM-d) δ = 11.94 (s, 1H), 7.84 (s, 1H), 7.73 (br s, 1H), 7.59 - 7.51 (m, 2H), 7.50 - 7.35 (m, 4H), 7.32 - 7.27 (m, 1H), 7.24 - 7.15 (m, 2H), 6.85 - 6.76 (m, 4H), 6.12 (dd, *J*=4.9, 9.7 Hz, 1H), 4.51 (d, *J*=5.8 Hz, 1H), 4.12 (q, *J*=7.2 Hz, 1H), 3.82 - 3.76 (m, 7H), 3.51 (dd, *J*=3.3, 6.4 Hz, 1H), 2.23 (dd, *J*=4.9, 12.7 Hz, 1H), 2.05 (s, 1H), 1.90 - 1.76 (m, 1H), 1.72 - 1.60 (m, 2H), 1.32 - 1.13 (m, 4H), 0.99 - 0.77 (m, 18H). ^{13}C NMR (75MHz, CHLOROFORM-d) δ = 158.63, 146.47, 136.79, 130.28, 129.14, 128.21, 127.78, 126.92, 113.18, 113.08, 113.05, 91.74, 86.12, 84.51, 72.78, 69.88, 55.27, 55.26, 40.82, 36.33, 25.76, 18.69, 18.41, 17.88, 17.85; LCMS (ESI) m/z: [M-H] calcd, 753.4; found, 752.3

Compound 58. Triethylamine (0.69 mL, 4.98 mmol, 2.5 eq) was added to a solution of compound **57** (1.53 g, 1.99 mmol) in dry THF (20 mL). The reaction was cooled in an ice bath. Triethylamine trihydrofluoride (1.62 mL, 9.9 mmol, 5 eq.) was added dropwise and the reaction was stirred for 16 hours and slowly warmed to room temperature. The reaction was quenched by adding triethylamine (0.65 mL) and concentrated under reduced pressure to obtained a crude yellow oil. Purification by flash chromatography (silica gel, 5g col, 0-10% methanol/dichloromethane + 1% Et_3N) afforded the product as a white solid 1.0 g, 77 % yield. ^{1}H NMR (300MHz, CHLOROFORM-d) δ = 12.11 (s, 1H), 8.82 (br s, 1H), 7.80 (s, 1H), 7.52 - 7.43 (m, 2H), 7.41 - 7.32 (m, 5H), 7.28 - 7.18 (m, 5H), 6.83 - 6.75 (m, 5H), 6.07 (t, *J*=6.6 Hz, 1H), 4.77 - 4.70 (m, 1H), 4.12 (q, *J*=7.2 Hz, 1H), 3.80 - 3.73 (m, 8H), 3.63 (t, *J*=4.2 Hz, 1H),

2.99 (br s, 1H), 2.77 - 2.60 (m, 1H), 2.48 - 2.30 (m, 2H), 2.05 (s, 1H), 1.80 (s, 1H), 1.29 - 1.07 (m, 9H), 0.86 (d, *J*=6.3 Hz, 4H). ^{13}C NMR (75MHz, METHANOL-d_4) δ = 181.78, 160.05, 149.30, 141.25, 139.72, 130.47, 129.17, 128.56, 127.83, 113.88, 92.38, 85.33, 82.43, 73.55, 73.15, 68.34, 62.28, 55.70, 42.11, 36.97, 20.20, 19.33; LCMS (ESI) m/z: [M-H] calcd, 639.3; found, 638.3

C**ompound 59.** Alcohol **58** (1.0 g, 1.50 mmol) was dissolved in dry DMF (5 mL) under an atmosphere of nitrogen. To this was added 1H-tetrazole (82 mg, 1.20 mmol, 0.8 eq) and 1-methylimidazole (29 µL, 37 mmol, 0.25 eq), followed by dropwise addition of 3-bis(diisopropylamino)phosphanyloxypropanenitrile (0.713 mL, 2.25 mmol, 1.5 eq.) This was stirred at room temperature for 90 minutes. Water (1 mL) was added to quench the reaction. The reaction was diluted with a mixture of toluene/hexanes (3:1) The organics were washed four times with a mixture of DMF/H_2O (3:2). The organics were then washed with saturated sodium bicarbonate solution, brine, dried over sodium sulfate and concentrated to a white foam. Purification by flash chromatography (silica gel, 5 g col, ethyl acetate + 1 % triethyl amine) gave the desired product as a white solid 1.20 g, 94 % yield. ^{1}H NMR (300MHz, CHLOROFORM-d) δ = 7.82 - 7.77 (m, 1H), 7.56 - 7.33 (m, 2H), 7.29 - 7.16 (m, 1H), 6.83 - 6.75 (m, 1H), 6.13 (dd, *J*=5.7, 8.6 Hz, 1H), 5.63 (s, 1H), 4.84 - 4.75 (m, 1H), 4.09 - 3.97 (m, 1H), 3.89 - 3.56 (m, 3H), 3.06 - 2.89 (m, 1H), 2.79 - 2.65 (m, 1H), 2.64 - 2.34 (m, 1H), 2.24 - 2.08 (m, 1H), 2.05 (s, 1H), 1.58 - 1.36 (m, 1H), 1.30 - 0.95 (m, 6H), 0.91 - 0.77 (m, 1H). ^{31}P NMR (121MHz, CHLOROFORM-d) δ = 149.44 (s, 1P), 148.41 (s, 1P), 147.36 (s, 1P). LCMS (ESI) m/z: [M-H] calcd, 839.4; found, 838.3

Scheme S8. Synthesis of (R)- and (S)-5’-Ethyl Thymidine Nucleosides.

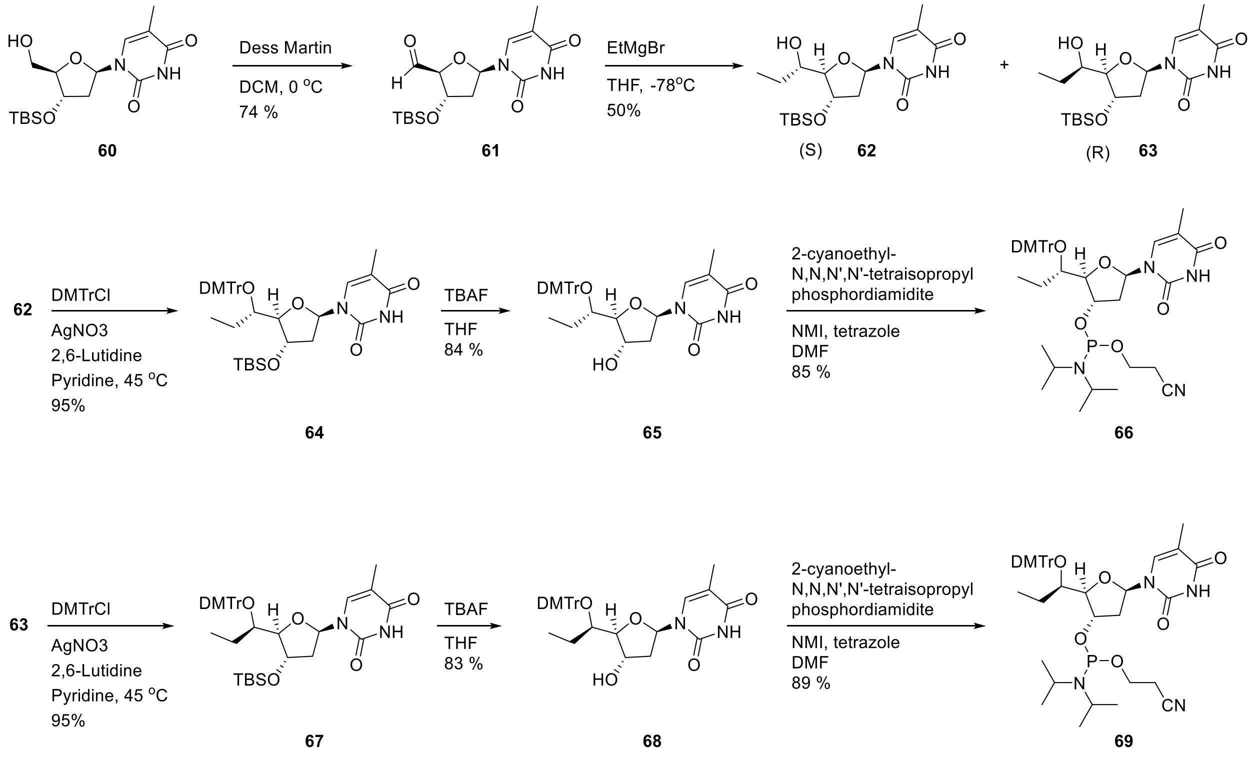

Synthesis of (R)- and (S)-5'-Ethyl Thymidine nucleosides were prepared using EtMgBr in a manner identical to the procedure reported in Kel'in, et al *J Org Chem* **2016,** 81, 2261-79.

C**ompound 61.** Starting material was dried over P_2O_5 under high vacuum for 16 hours prior to use. 3'-OTBDMS thymidine **60** (2 g, 5.61 mmol) was dissolved in dry DCM (50 ml) and the reaction was cooled in an ice bath with stirring under nitrogen. Dess-Martin periodane (3.57 g, 8.42 mmol, 1.5 eq.) was added and the reaction was allowed to stir for 3 hour at 0 °C. The ice bath was removed, and the reaction was allowed to warm to room temperature. Excess Dess-Martin reagent was quenched by pouring the cold reaction mixture into a mixture of 5% aq. sodium thiosulfate (20 mL) and saturated aq. $NaHCO_3$ (20 mL). The reaction was concentrated under reduced pressure and then diluted with EtOAc (75 ml). The organic layer was washed with brine (100 ml) and dried over $MgSO_4$. The crude product was filtered through a small plug of silica gel, and the solvent was removed under reduced pressure. The crude material was used

without purification in the next step. Yield: 1.4 grams (74 %). ^{1}H NMR (300MHz, CHLOROFORM-d) δ = 9.77 (s, 1H), 8.58 (br s, 1H), 7.58 - 7.28 (m, 1H), 6.32 (dd, *J*=5.8, 8.1 Hz, 1H), 4.68 (td, *J*=1.9, 5.4 Hz, 1H), 4.49 (d, *J*=1.9 Hz, 1H), 4.13 (d, *J*=7.2 Hz, 1H), 2.38 - 2.20 (m, 1H), 2.13 - 1.84 (m, 5H), 1.27 (t, *J*=7.1 Hz, 1H), 1.01 - 0.83 (m, 11H), 0.19 - 0.00 (m, 8H). ^{13}C NMR (75MHz, DMSO-d_6) δ = 200.92, 163.72, 150.52, 150.43, 137.20, 109.68, 109.53, 90.92, 88.87, 86.83, 72.97, 25.80, 25.70, 25.62, 17.67, 12.11, -3.21, -4.89, -4.94, -4.96. LCMS (ESI) m/z: [M+H] calcd, 354.5; found, 355.2

C**ompound 62 and 63.** Starting material was dried over P_2O_5 overnight prior to use. Aldehyde **61** (1.48 g, 4.02 mmol) was dissolved in dry THF 20 ml) and was cooled to - 20 °C with stirring under nitrogen. Ethylmagnesium bromide (3M in Et_2O, 5.6 ml, 17 mmol, 4 eq.) was added dropwise via syringe and the reaction was stirred for 6 hours at 0 °C under nitrogen. The reaction was quenched by slow addition of saturated aqueous NH_4Cl (100 ml). The reaction was diluted with EtOAc (70 ml) and was transferred to a separatory funnel. The reaction mixture was washed with a mixture of water and saturated aqueous NH_4Cl (1:1, 3 x 200 ml), followed by brine (1 x 200 ml). The organics were collected, dried over $MgSO_4$, filtered, and concentrated under reduced pressure. Yield: 850 mg (55%). LCMS suggests ~ 1:1 ratio of R- and S-isomers. The isomers were separated by silica gel chromatography. Solvent A = 1% triethylamine in hexanes and solvent B = Et_2O. Gradient from 50% B to 100% B over 15 column volumes and monitor by TLC (75% Et_2O in hexanes with 1% triethylamine). Consistent with the literature protocol, the S-isomer eluted first. Yield: 300 mg S-isomer (14 %) , and 450 mg R-isomer (21%)

(S)-5'-ethyl T TBDMS - ^{1}H NMR (300MHz, CHLOROFORM-d) δ = 7.42 (d, *J*=1.2 Hz, 1H), 6.13 (t, *J*=6.9 Hz, 1H), 4.56 - 4.46 (m, 1H), 3.83 - 3.78 (m, 1H), 3.61 (br d, *J*=2.3 Hz, 1H), 3.26 (q, *J*=7.3 Hz, 1H), 2.57 (d, *J*=7.2 Hz, 1H), 2.44 - 2.29 (m, 1H), 2.28 - 2.08 (m, 1H), 1.92 (d, *J*=1.2 Hz, 3H), 1.71 - 1.49 (m, 3H), 1.32 (t, *J*=7.2 Hz, 3H), 1.07 - 0.83 (m, 9H), 0.11 - -0.03 (m, 6H). LCMS (ESI) m/z: [M-H] calcd, 384.5; found, 385.2.

(R)-5'-ethyl T TBDMS - ^{1}H NMR (300MHz, CHLOROFORM-d) δ = 8.35 (br s, 1H), 7.41 - 7.32 (m, 1H), 6.15 - 6.05 (m, 1H), 4.56 - 4.46 (m, 1H), 3.95 - 3.73 (m, 2H), 2.74 (br s, 1H), 2.44 - 2.32 (m, 1H), 2.28 - 2.09 (m, 1H), 1.95 - 1.90 (m, 3H), 1.78 - 1.43 (m, 4H), 1.11 - 0.88 (m,

13H), 0.14 - 0.07 (m, 6H). ^{13}C NMR (75MHz, CHLOROFORM-d) δ = 163.38, 150.21, 137.38, 111.00, 90.23, 87.30, 73.39, 70.78, 40.52, 26.39, 25.72, 25.67, 17.79, 12.51, 10.44, -4.43, -4.88. LCMS (ESI) m/z: [M-H] calcd, 384.5; found, 385.2.

C**ompound 64.** Starting material was dried over P_2O_5 for 16 hours under high vacuum prior to use. Alcohol **62** (300 mg, 0.78 mmol) was dissolved in pyridine (2.5 ml) and lutidine (0.275 ml, 2.34 mmol, 3 eq.). DMT-Cl (800 mg, 2.34 mmol, 3 eq.) was added to the reaction followed by $AgNO_3$ (100 mg, 0.59 mmol, 0.75 eq.). The reaction was purged with argon, capped tightly, and allowed to stir at 40 °C for 4 days. Upon completion, the reaction was cooled to room temperature, diluted with EtOAc (50 ml), and filtered through a plug of celite to remove silver salts. The filtrate was transferred to a separatory funnel and washed with saturated aqueous $NaHCO_3$ (2 x 25 ml) and brine (1 x 25 ml). The organics were collected, dried over $MgSO_4$, filtered, and concentrated under reduced pressure. The crudes material was purified by silica gel chromatography, neutralize with 5% triethyalamine in hexanes, and purified using an EtOAc/hexanes gradient. Yield: 507 mg (94.6 %). ^{1}H NMR (300MHz, CHLOROFORM-d) δ = 8.40 - 8.23 (m, 1H), 7.50 - 7.28 (m, 8H), 7.26 - 7.04 (m, 3H), 6.92 - 6.74 (m, 5H), 6.41 - 6.21 (m, 1H), 4.88 - 4.82 (m, 1H), 3.98 - 3.76 (m, 9H), 3.17 (td, *J*=3.0, 9.2 Hz, 1H), 2.32 - 2.00 (m, 2H), 1.65 - 1.21 (m, 6H), 0.94 - 0.71 (m, 11H), 0.64 - 0.51 (m, 3H). ^{13}C NMR (75MHz, CHLOROFORM-d) δ = 163.42, 158.60, 158.56, 150.08, 146.60, 136.98, 136.63, 135.57, 130.18, 130.13, 129.11, 127.97, 127.83, 127.76, 126.85, 113.11, 113.07, 113.02, 110.89, 87.67, 86.51, 83.61, 75.88, 71.22, 55.21, 41.13, 25.69, 24.20, 17.83, 11.78, 10.09, -4.24, -4.86. LCMS (ESI) m/z: [M-H] calcd, 686.6; found, 686.3.

C**ompound 65.** Compound **64** (500 mg, 0.73 mmol) was dissolved in dry THF (20 ml). A solution of TBAF was added (1 M in THF, 3.50 ml, 3.50 mmol, 1.5 eq.) and the reaction was stirred at room temperature overnight under nitrogen. Upon completion, the solvent was removed under reduced pressure. The crude material was dissolved in EtOAc (100 ml) and was transferred to a separatory funnel. The reaction was washed with saturated aqueous $NaHCO_3$ (50 ml), water (50 ml), and then brine (50 ml). The organics were collected, dried over $MgSO_4$, filtered, and concentrated under reduced pressure. The crude material was purified by silica gel

chromatography using a MeOH/DCM gradient. Yield: 417 mg (84 %). ^{1}H NMR (300MHz, CHLOROFORM-d) δ = 8.79 (s, 1H), 7.51 - 7.28 (m, 7H), 7.26 - 7.12 (m, 2H), 6.94 (d, *J*=1.1 Hz, 1H), 6.84 (dd, *J*=1.9, 8.9 Hz, 5H), 6.37 - 6.15 (m, 1H), 4.68 - 4.57 (m, 1H), 3.92 - 3.72 (m, 8H), 3.26 - 3.14 (m, 1H), 2.92 - 2.76 (m, 1H), 2.57 - 2.25 (m, 2H), 2.19 - 2.01 (m, 1H), 1.94 - 1.83 (m, 1H), 1.75 - 1.39 (m, 4H), 1.33 - 1.20 (m, 1H), 1.10 - 0.81 (m, 1H), 0.75 - 0.57 (m, 3H). ^{13}C NMR (75MHz, CHLOROFORM-d) δ = 163.52, 158.63, 158.60, 150.25, 146.44, 136.86, 136.54, 135.26, 130.33, 130.20, 130.15, 129.11, 128.10, 127.94, 127.82, 127.74, 126.91, 113.16, 113.13, 113.11, 111.11, 86.87, 86.75, 83.51, 76.16, 71.46, 55.21, 40.61, 24.39, 11.92, 9.77. LCMS (ESI) m/z: [M-H] calcd, 572.6; found, 571.3.

C**ompound 66.** Alcohol **65** (350 mg, 0.611 mmol) and tetrazole (3.43 mg, 0.49 mmol, 0.8 eq.) were dried over P_2O_5 for 16 hours under high vacuum. The reagents were dissolved in dry DMF (9 ml) with stirring under nitrogen. N-Methylimidazole (12.1 µl, 0.497 mmol, 0.25 eq.) and 3-bis(diisopropylamino)phosphanyloxypropanenitrile (291 µl, 0.917 mmol, 1.5 eq.) were added via syringe and the reaction was allowed to stir at room temperature under nitrogen for 4 hours. The reaction was diluted with EtOAc (50 ml) and triethylamine (1 ml) and was transferred to a separatory funnel. The reaction was washed with saturated aqueous $NaHCO_3$ (50 ml), water (50 ml) and brine (50 ml). The organics were collected, dried over $MgSO_4$, filtered, and concentrated under reduced pressure. The crude material was purified by dissolving in a minimum amount of DCM and precipitating by slow addition into hexanes. The product was dried under high vacuum to give the desired product as a brittle foam. Yield: 399 mg (85%). ^{1}H NMR (300MHz, CHLOROFORM-d) δ = 9.00 - 8.62 (m, 1H), , 7.96 - 7.86 (m, 1H), 7.47 - 7.13 (m, 10H), 6.91 - 6.75 (m, 4H), 6.51 - 6.25 (m, 1H), 4.55 - 4.32 (m, 1H), 4.28 - 3.98 (m, 2H), 3.98 - 3.86 (m, 1H), 3.83 - 3.48 (m, 9H), 3.35 - 3.07 (m, 1H), 2.78 - 2.71 (m, 1H), 2.66 - 2.29 (m, 6H), 1.89 - 1.58 (m, 4H), 1.47 - 1.34 (m, 1H), 1.31 - 1.05 (m, 12H), 0.54 (q, *J*=7.2 Hz, 3H). ^{13}C NMR (75MHz, CHLOROFORM-d) δ = 163.78, 158.63, 158.61, 150.29, 146.18, 137.82, 136.69, 136.63, 136.39, 136.28, 135.58, 135.53, 130.34, 130.29, 130.21, 130.15, 128.21, 128.18, 128.01, 127.94, 130.41, 127.66, 128.99, 126.93, 125.25, 117.49, 117.21, 113.19, 113.15, 84.20, 58.08, 55.24, 55.19, 43.26, 24.60, 24.54 (2C), 24.39, 21.41, 20.34, 20.24, 20.02, 19.92, 12.23, 9.90. ^{31}P

NMR (121MHz, CHLOROFORM-d) δ = 149.33 (s, 1P), 148.88 (s, 1P). LCMS (ESI) m/z: [M-H] calcd, 772.9; found, 771.4.

C**ompound 67.** Starting material was dried over P_2O_5 for 16 hours under high vacuum prior to use. Alcohol **63** (300 mg, 0.78 mmol) was dissolved in pyridine (2.5 ml) and lutidine (0.3 ml, 2.58 mmol, 3.31 eq.). DMT-Cl (1 g, 2.95 mmol, 3.78 eq.) was added to the reaction followed by $AgNO_3$ (100 mg, 0.59 mmol, 0.75 eq.). The reaction was purged with argon, capped tightly, and was allowed to stir at 40 °C for 4 days. Upon completion, the reaction was cooled to room temperature, diluted with EtOAc (50 ml), and filtered through a plug of celite to remove silver salts. The filtrate was transferred to a separatory funnel and washed with saturated aqueous $NaHCO_3$ (2 x 25 ml) and brine (1 x 25 ml). The organics were collected, dried over $MgSO_4$, filtered and concentrated under reduced pressure. The crudes material was purified by silica gel chromatography, neutralize with 5% triethyalamine in hexanes, and purified using an EtOAc/hexanes gradient. Yield: 368 g (69 %). ^{1}H NMR (300MHz, CHLOROFORM-d) δ = 7.93 (s, 1H), 7.53 - 7.43 (m, 2H), 7.42 - 7.28 (m, 6H), 7.26 - 7.17 (m, 2H), 7.08 - 7.04 (m, 1H), 6.92 - 6.79 (m, 5H), 6.24 (dd, *J*=5.4, 8.9 Hz, 1H), 4.88 - 4.83 (m, 1H), 3.90 (t, *J*=2.8 Hz, 1H), 3.82 - 3.77 (m, 7H), 3.17 (br d, *J*=9.1 Hz, 1H), 2.18 (br d, *J*=1.7 Hz, 1H), 1.78 (d, *J*=1.2 Hz, 1H), 1.57 (s, 4H), 1.42 (d, *J*=1.2 Hz, 6H), 0.97 - 0.87 (m, 9H), 0.80 (s, 2H), 0.59 (t, *J*=7.4 Hz, 4H), 0.17 (d, *J*=5.9 Hz, 6H). ^{13}C NMR (75MHz, CHLOROFORM-d) δ = 163.24, 158.62, 158.58, 149.97, 146.60, 137.00, 136.65, 135.58, 130.20, 130.14, 127.99, 127.77, 126.86, 113.13, 113.08, 113.05, 110.88, 87.70, 86.53, 83.63, 77.20, 75.89, 71.24, 55.22, 41.13, 25.70, 24.21, 17.84, 11.78, 10.09, -4.24, -4.84. LCMS (ESI) m/z: [M-H] calcd, 686.9; found, 686.3.

C**ompound 68.** Compound **67** (450 mg, 0.655 mmol) was dissolved in dry THF (4 ml). A solution of TBAF was added (1 M in THF, 0.6 ml, 6.0 mmol, 9 eq.) and the reaction was stirred at room temperature overnight under nitrogen. Upon completion, the solvent was removed under reduced pressure. The crude material was dissolved in EtOAc (100 ml) and was transferred to a separatory funnel. The reaction was washed with saturated aqueous $NaHCO_3$ (50 ml), water (50 ml), and brine (50 ml). The organics were collected, dried over $MgSO_4$, filtered, and concentrated under reduced pressure. The crude material was purified by silica gel

chromatography using a MeOH/DCM gradient. Yield: 311 mg (83 %). ^{1}H NMR (300MHz, CHLOROFORM-d) δ = 8.28 (s, 1H), 7.51 - 7.28 (m, 8H), 7.26 - 7.16 (m, 2H), 6.92 - 6.80 (m, 5H), 6.27 (dd, *J*=5.8, 8.0 Hz, 1H), 4.63 - 4.55 (m, 1H), 4.17 - 4.09 (m, 1H), 3.90 (t, *J*=3.8 Hz, 1H), 3.82 - 3.78 (m, 6H), 3.27 - 3.17 (m, 1H), 2.39 - 2.22 (m, 1H), 2.18 - 2.04 (m, 3H), 1.86 (d, *J*=1.2 Hz, 1H), 1.61 (s, 2H), 1.51 (d, *J*=1.2 Hz, 4H), 1.27 (t, *J*=7.1 Hz, 1H), 0.70 (t, *J*=7.4 Hz, 3H). ^{13}C NMR (75MHz, CHLOROFORM-d) δ = 163.28, 158.69, 158.65, 150.03, 146.42, 136.85, 136.54, 135.22, 130.35, 130.22, 130.16, 128.12, 128.04, 127.96, 127.85, 126.95, 113.20, 113.14, 111.04, 86.81, 86.72, 83.51, 77.20, 76.13, 71.57, 60.39, 55.22, 40.56, 24.43, 21.04, 14.19, 11.96, 9.70. LCMS (ESI) m/z: [M-H] calcd, 572.7; found, 571.3.

C**ompound 69.** Alcohol **68** (310 mg, 0.54 mmol) and tetrazole (3.03 mg, 0.43 mmol, 0.8 eq.) were dried over P_2O_5 for 16 hours under high vacuum. The reagents were dissolved in dry DMF (9 ml) with stirring under nitrogen. N-Methylimidazole (11 µl, 0.135 mmol, 0.25 eq.) and 3-bis(Diisopropylamino)phosphanyloxypropanenitrile (258 µl, 0.812 mmol, 1.5 eq.) were added via syringe and the reaction was allowed to stir at room temperature under nitrogen for 4 hours. The reaction was diluted with EtOAc (50 ml) and triethylamine (1 ml) and was transferred to a separatory funnel. The reaction was washed with saturated aqueous $NaHCO_3$ (50 ml), water (50 ml), and brine (50 ml). The organics were collected, dried over $MgSO_4$, filtered, and concentrated under reduced pressure. The crude material was purified by dissolving in a minimum amount of DCM and precipitating by slow addition into hexanes. The product was dried under high vacuum to give the desired product as a brittle foam. Yield: 370 mg (89%). ^{1}H NMR (300MHz, CHLOROFORM-d) δ = 7.50 - 7.13 (m, 10H), 7.08 - 6.90 (m, 1H), 6.82 (dd, *J*=2.6, 8.9 Hz, 4H), 6.42 - 6.16 (m, 1H), 5.10 - 5.00 (m, 1H), 4.23 - 4.01 (m, 1H), 3.91 - 3.57 (m, 9H), 3.52 (dt, *J*=4.0, 7.1 Hz, 1H), 3.19 - 3.08 (m, 1H), 2.85 - 2.61 (m, 2H), 2.59 - 2.29 (m, 2H), 2.25 - 1.96 (m, 1H), 1.82 - 1.55 (m, 1H), 1.52 - 1.45 (m, 1H), 1.41 - 1.11 (m, 17H), 0.69 - 0.48 (m, 3H). ^{13}C NMR (75MHz, CHLOROFORM-d) δ = 163.44, 158.61, 150.19, 150.12, 146.57, 136.97, 136.94, 136.61, 136.49, 135.53, 135.43, 130.42, 130.20, 130.16, 130.13, 129.01, 128.19, 128.00, 127.94, 127.79, 127.67, 126.85, 125.27, 11113.10, 112.97, 111.01, 83.36, 58.43, 58.20, 55.20 (2C), 43.36, 24.71, 24.46 (2C), 24.34, 22.97, 22.89, 22.85, 21.43, 20.39, 11.66, 10.17. ^{31}P

NMR (121MHz, CHLOROFORM-d) δ = 148.62 (s, 1P), 147.96 (s, 1P). LCMS (ESI) m/z: [M-H] calcd, 772.9; found, 771.4.

Scheme S9. Synthesis of 5'-(R) Ethyl Cytidine Nucleosides.

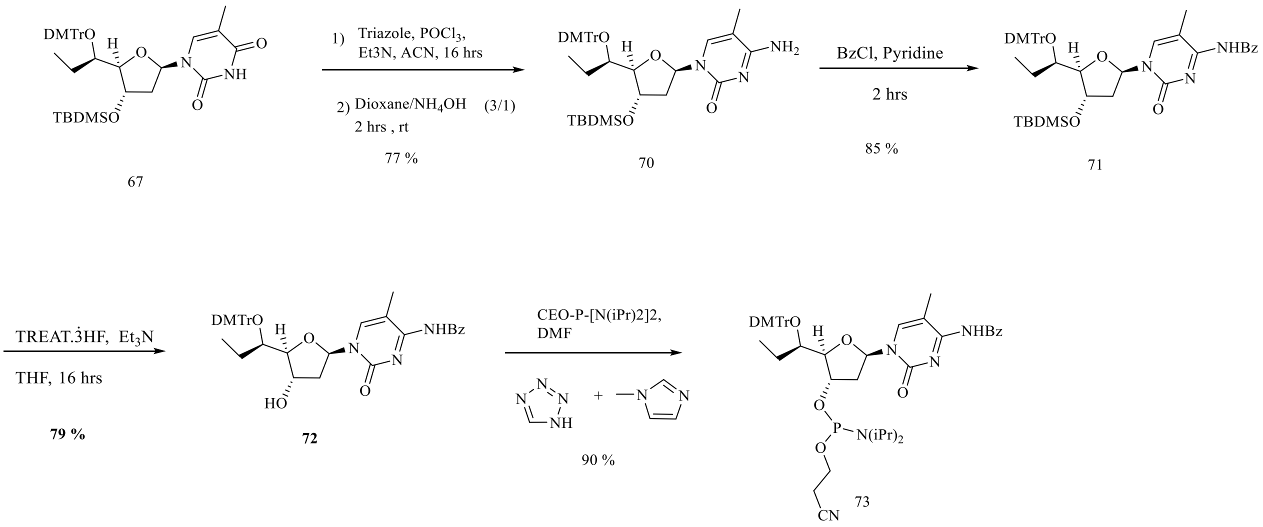

Compound 70. $POCl_3$ (5.5 mL, 60.2 mmol, 8 eq) was added dropwise to a suspension of 1,2,4-1H-triazole (17.70 g, 256 mmol, 34 eq.) in acetonitrile (200 mL) under an atmosphere of nitrogen at room temperature and stirred reaction for 20 minutes. The reaction was cooled down with ice bath at 0°C , triethylamine (42 mL, 301 mmol, 40 eq.) was added dropwise, the ice bath was removed and continued to stir reaction for 30 minutes at room temperature. The reaction was cooled down with an ice bath and a solution of Compound **(1)** 1-((2R,4S,5R)-5-((R)-1-(bis(4-methoxyphenyl)(phenyl)methoxy)propyl)-4-((tert-butyldimethylsilyl)oxy)tetrahydrofuran-2-yl)-5-methylpyrimidine-2,4(1H,3H)-dione (5.17 g, 7.53 mmol) in acetonitrile (30 mL) was added dropwise to the reaction. This was stirred at room temperature for 16 hours. The reaction was concentrated to small volume under reduced pressure, diluted with ethyl acetate and the organic layer was washed with aqueous saturated sodium bicarbonate (2x), water, brine and concentrated to afford the desired crude material as a yellow oil.

Without any further purification, the crude material was suspended in dioxane/NH_4OH (40 mL/10 mL) and stirred at room temperature for 2 hours. TLC in EtOAc/hexane (6/4) indicated reaction was completed. The solvents were concentrated under reduce pressure and the remaining oil was diluted with Ethyl acetate and washed with water (1x200 ml), sat. NaHCO3 (1x200 ml), brine (1x200 ml), dried over Na_2SO_4, filtered and concentrated under reduce pressure to obtain a crude oil. The crude material was dissolved in DCM and purified by silica gel chromatography (silica gel, 50g col, 40-100% EtOAc/Hexane) afforded the desired product as a white solid. 4.10 g, 77 % yield. ^{1}H NMR (300MHz, CHLOROFORM-d) δ = 7.33 (br d, *J*=8.1 Hz, 3H), 7.28 - 7.00 (m, 14H), 6.71 - 6.59 (m, 6H), 6.09 (br t, *J*=6.8 Hz, 2H), 4.71 - 4.61 (m, 1H), 3.74 (br s, 1H), 3.67 - 3.60 (m, 9H), 3.11 - 2.83 (m, 2H), 2.31 - 2.02 (m, 2H), 1.99 - 1.68 (m, 3H), 1.55 (s, 1H), 1.38 - 1.15 (m, 9H), 1.10 (s, 1H), 0.77 - 0.72 (m, 12H), 0.70 - 0.56 (m, 2H), 0.43 (br t, *J*=7.2 Hz, 4H), ^{13}C NMR (75MHz, CHLOROFORM-d) δ = 165.14, 158.54, 155.74, 146.74, 138.52, 137.18, 136.83, 130.28, 130.19, 128.07, 127.74, 126.78, 113.09, 113.05, 101.20, 87.30, 86.38, 84.72, 75.81, 71.10, 55.24, 42.37, 25.74, 24.21, 17.85, 12.62, 10.26; LCMS (ESI) m/z: [M-H] calcd, 685.4; found, 684.3

Compound 71. Compound **70** (3.27 g, 4.81 mmol) was dissolved in anhydrous pyridine (20 mL) under nitrogen at 0 °C. Benzoyl chloride (0.61 mL, 5.29 mmol, 1.1 3q.) was added dropwise and continued to stir the reaction for 16 hours at room temperature. TLC analysis in EtOAc/Hexane (6/4) indicated reaction was completed. The reaction was cooled ice bath and H_2O (20 ml) was added slowly followed by EtOAc (50 mL). The solution was transferred to a separatory funnel and was washed with water (50 mL). The aqueous layer was discarded and the organics were washed with sat. $NaHCO_3$ and brine. The organics were dried over Na_2SO_4, filtered and concentrated under reduce pressure to obtained crude oil. The crude material was purified by silica gel chromatography (silica gel, 50g col, 40-70% EtOAc/hexanes) to afford the desired product as a white solid. Yield: 3.20 g (85 %.)· ^{1}H NMR (300MHz, CHLOROFORM-d) δ = 8.31 (d, *J*=7.3 Hz, 1H), 8.12 (d, *J*=7.7 Hz, 4H), 7.64 - 7.54 (m, 3H), 7.53 - 7.37 (m, 7H), 7.31 - 7.24 (m, 9H), 7.17 (d, *J*=8.8 Hz, 5H), 6.83 (d, *J*=8.8 Hz, 4H), 3.80 (s, 8H), 2.11 (s, 3H), 1.93 (s, 1H), 1.25 (s, 3H), 1.12 - 0.97 (m, 4H), 0.90 (s, 10H), ^{13}C NMR (75MHz, CHLOROFORM-d) δ = 170.62, 158.64, 147.31, 139.45, 138.38, 133.63, 132.18, 130.18, 129.92, 129.34, 129.13, 128.68, 128.47, 128.13, 127.86, 127.77, 127.36, 127.09, 113.17, 111.95, 90.39, 87.81, 81.45, 73.47,

70.80, 55.26, 40.85, 26.51, 25.68, 17.81, 13.67, 10.45; LCMS (ESI) m/z: [M-H] calcd, 789.4; found, 788.3

Compound 72. TEA (0.90 mL, 6.32 mmol, 2.5 eq.) was added to a solution of compound **71** 2.0 g, 2.5 mmol) in tetrahydrofuran (20.0 mL). The reaction was cooled down with an ice bath under an atmosphere of nitrogen. Triethylamine trihydrofluoride (2.0 mL, 12.65 mmol, 5 eq.) was slowly added and continued to stir reaction for 16 hours at room temperature. TEA (0.90 ml) was added to the reaction and the solvent was removed under reduced pressure to obtained crude solid. This material was dissolved in EtOAc (30 ml) and washed with water (50 ml). The aqueous layer was discarded and the organics were washed with saturated $NaHCO_3$ and brine. The organics were dried over Na_2SO_4, filtered and concentrated under reduce pressure to obtained a crude oil. The crude product was purified by flash chromatography (silica gel, 20g col, 20-100 % EtOAc/hexanes) to afford the desired product as a white solid. Yield. 1.71 g (79 %). ^{1}H NMR (300MHz, CHLOROFORM-d) δ = 8.35 - 8.26 (m, 3H), 7.57 - 7.35 (m, 9H), 7.33 - 7.22 (m, 10H), 7.22 - 7.11 (m, 6H), 6.87 - 6.79 (m, 7H), 6.18 (s, 2H), 3.86 - 3.75 (m, 13H), 2.39 (s, 5H), 2.11 (s, 6H), 1.69 (s, 5H), 1.25 (s, 2H), 1.05 (t, *J*=7.4 Hz, 4H), ^{13}C NMR (75MHz, CHLOROFORM-d) δ = 159.62, 158.65, 147.33, 139.47, 137.86, 132.52, 130.26, 130.17, 129.93, 129.15, 128.15, 127.99, 127.86, 127.78, 127.10, 113.23, 113.18, 112.10, 89.04, 86.83, 81.45, 73.72, 70.88, 55.27, 40.46, 26.73, 13.70, 10.11; LCMS (ESI) m/z: [M-H] calcd, 675.3; found, 674.2

Compound 73. 1H-Tetrazole (0.110 g, 1.60 mol, 0.8 eq) and 1-Methylimidazole (40 µL, 0.50 mmol, 0.25 eq) were added to a solution of compound **72** (1.35 g, 2.0 mmol) in DMF (15 mL) at room temperature under an atmosphere of nitrogen. 2-Cyanoethyl-N,N,N',N'-tetraisopropyl-phosphorodiamidite (0.90 mL, 3.0 mmol, 1.5 eq) was then added drop-wise and the reaction was stirred at room temperature for 3 hrs. Water (0.5.0 mL) was added to quench the reaction. A 3:1 mixture of toluene/hexanes (30 mL) was added and the organic layer was washed (4x 20 mL) with a 3:2 mixture of DMF/H_2O. The organics were washed with saturated $NaHCO_3$ and brine. The organics were dried over Na_2SO_4, filtered and concentrated under reduce pressure to give a white foam. Purification by flash chromatography (silica gel, 25g col, 40% ethyl acetate/hexanes

+ 1 % triethylamine) afforded the desired white solid. Yield: 1.6 g (90 %). ^{1}H NMR (300MHz, CHLOROFORM-d) δ = 8.34 - 8.21 (m, 2H), 7.55 - 7.34 (m, 9H), 7.32 - 7.17 (m, 6H), 6.83 (dd, *J*=2.3, 8.9 Hz, 4H), 3.78 (d, *J*=3.2 Hz, 10H), 2.64 (s, 3H), 1.55 (d, *J*=5.3 Hz, 5H), 1.36 - 1.11 (m, 15H), 0.56 (s, 3H). ^{31}P NMR (121MHz, CHLOROFORM-d) δ = 148.68 (s, 1P), 148.02 (s, 1P), LCMS (ESI) m/z: [M-H] calcd, 875.4; found, 874.0

Scheme S10. Synthesis of 5'-(R/S) Ethyl Adenosine Nucleosides.

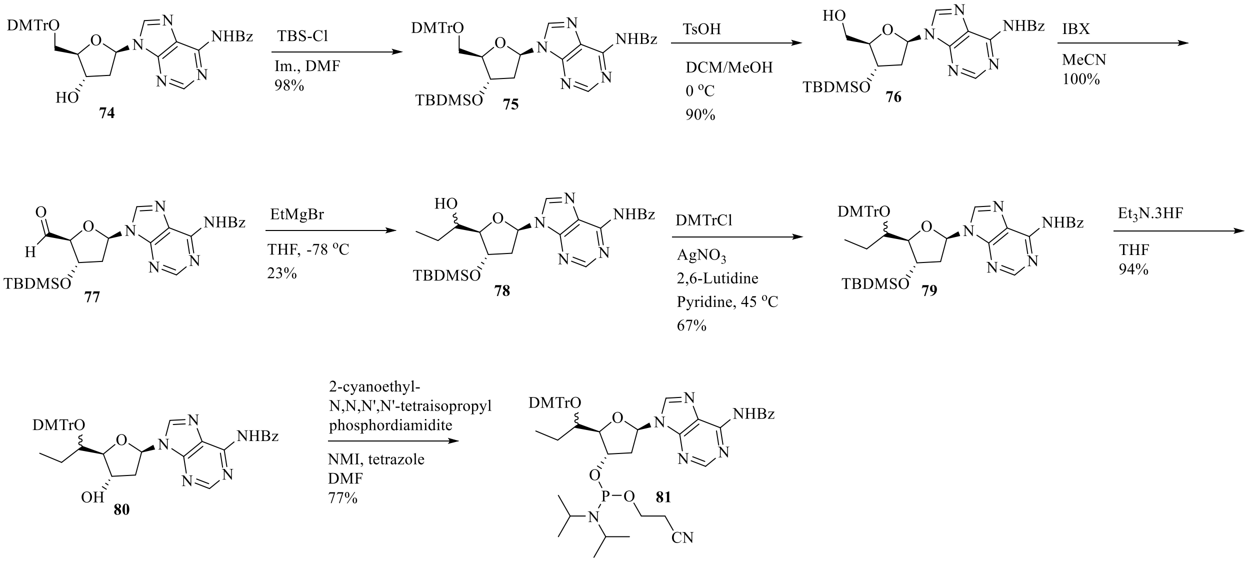

Compound 75. 5'-O-DMT-N6-Benzoyl-2'- deoxyadenosine (40.0 g, 60.8 mmol) was dissolved in DMF (304 mL). Imidazole (8.28 g, 122 mmol) was added followed by tert-butyl-chloro-dimethyl-silane (11.0 g, 73.0 mmol). The reaction was stirred for 16 hours. The reaction was slowly poured onto 400 mL of ice. Hexanes was added (800 mL), and the mixture was stirred 90 minutes to precipitate. The solvent was removed (decant). The white solid was washed with hexanes (2 x 200 ml). The white solid was then co-evaporated with toluene under reduced pressure at 60 °C to give the desired product as a white foam. Yield: 45.9 grams (97.7 %). ^{1}H NMR (300 MHz, CHLOROFORM-d) δ 9.03 (s, 1H), 8.76 (s, 1H), 8.23 (s, 1H), 7.92-8.11 (m, 2H), 7.48-7.67 (m, 3H), 7.37-7.44 (m, 2H), 7.27-7.33 (m, 4H), 7.15-7.25 (m, 3H), 6.73-6.90 (m, 4H), 6.49 (t, *J*=6.53 Hz, 1H), 4.56-4.73 (m, 1H), 4.06-4.21 (m, 1H), 3.78 (s, 6H), 3.24-3.48 (m, 2H), 2.80 (td, *J*=6.42, 13.15 Hz, 1H), 2.47 (ddd, *J*=3.71, 6.05, 13.03 Hz, 1H), 0.88 (s, 9H), 0.07

(s, 3H), 0.03 (s, 3H). ^{13}C NMR (75 MHz, CHLOROFORM-d) δ 171.1, 164.6, 158.5, 152.6, 151.5, 149.5, 144.5, 141.6, 135.7, 133.7, 132.7, 130.0, 128.8, 128.1, 127.9, 126.9, 123.5, 113.2, 87.0, 86.5, 84.9, 72.5, 63.2, 60.4, 55.2, 40.7, 25.8, 18.0, 14.2, -4.7, -4.8. LCMS (ESI) m/z: [M+H] calcd, 772.4; found, 772.4.

Compound 76. Intermediate **75** (1.00 g, 1.30 mmol) was dissolved in 3:1 DCM/methanol (129 mL) and was cooled in an ice bath. Toluene-4-sulfonic acid (0.296 g, 1.55 mmol) was added and the reaction was stirred at 0 °C for 30 minutes. Sodium carbonate (206 mg, 1.5 eq) was added at 0 °C and the reaction was stirred until the orange color has disappeared. The solvents were removed under reduced pressure. Purification by flash chromatography (silica gel, 50g col, 0-5% methanol/dichloromethane) gave the desired product as a white solid. Yield: 0.545 g (89.6 %). ^{1}H NMR (300 MHz, CHLOROFORM-d) δ 9.01 (s, 1H), 8.79 (s, 1H), 8.10 (s, 1H), 7.98-8.07 (m, 2H), 7.47-7.71 (m, 3H), 6.37 (dd, *J*=5.38, 9.47 Hz, 1H), 5.75 (dd, *J*=1.98, 11.46 Hz, 1H), 4.73 (d, *J*=4.86 Hz, 1H), 4.18 (s, 1H), 3.99 (br d, *J*=12.93 Hz, 1H), 3.70-3.86 (m, 1H), 3.06 (ddd, *J*=5.12, 9.50, 13.02 Hz, 1H), 2.19-2.38 (m, 1H), 0.92-1.02 (m, 9H), 0.14 (s, 6H). ^{13}C NMR (75 MHz, CHLOROFORM-d) δ 164.7, 152.1, 150.8, 150.3, 142.6, 133.5, 132.9, 128.8, 128.0, 124.5, 90.3, 87.7, 73.8, 63.1, 41.4, 25.8, 18.0, -4.7, -4.8. LCMS (ESI) m/z: [M+H] calcd, 470.2; found, 470.2.

Compound 77. 2-Iodoxybenzoic acid (IBX) (11.9 g, 42.6 mmol) was added to a solution of intermediate **76** (10.0 g, 21.3 mmol) in acetonitrile (213 mL). The reaction was heated at 70 °C for 4 hours. After cooling to room temperature, the reaction mixture was filtered through a plug of celite. The solvent was removed under reduced pressure to give the crude reaction mixture as a yellow foam. The crude material was dissolved in ethyl acetate and washed with water. The organics were dried over magnesium sulfate and concentrated under reduced pressure to give the desired product as a pale yellow foam. Compound **77** was used in the next step without further purification. Yield: 10.3 g (quantitative)

Compound 78. A solution of **77** (10.3 g, 22.0 mmol) in tetrahydrofuran (220 mL) was cooled to -20 °C under an atmosphere of nitrogen. EtMgBr in diethyl ether (3.00 M, 14.7 mL, 44.1 mmol) was added and the reaction was maintained at -20 °C for 2.5 hours. The reaction was quenched

with saturated ammonium chloride solution. The aqueous layer was extracted with ethyl acetate and the combined organics were concentrated under reduced pressure to give a thick oil. The crude material was purified by flash chromatography (silica gel, 220g col, 0-60% ethyl acetate/hexanes) to give **77** as a white foam. Yield: 2.46 g (22%.). ^{1}H NMR (300 MHz, CHLOROFORM-d) δ 9.08 (s, 1H), 8.79 (d, *J*=6.53 Hz, 1H), 8.10 (d, *J*=2.56 Hz, 1H), 8.03 (d, *J*=7.04 Hz, 2H), 7.59-7.71 (m, 1H), 7.49-7.58 (m, 2H), 6.34 (ddd, *J*=5.18, 9.76, 12.58 Hz, 1H), 5.27-6.17 (m, 1H), 4.68 (t, *J*=4.48 Hz, 1H), 4.09 (s, 1H), 3.53-3.90 (m, 1H), 2.94-3.17 (m, 1H), 2.14-2.31 (m, 1H), 1.49-1.60 (m, 2H), 0.97-1.15 (m, 3H), 0.93 (d, *J*=1.41 Hz, 9H), 0.13 (s, 6H). ^{13}C NMR (75 MHz, CHLOROFORM-d) δ 164.7, 152.1, 151.9, 150.7, 150.3, 150.3, 142.7, 133.5, 132.9, 128.0, 128.8, 93.1, 91.2, 87.7, 87.4, 75.0, 73.6, 73.4, 71.8, 41.5, 40.8, 27.4, 26.1, 25.8, 25.7, 25.7, 18.0, 17.9, 10.9, 10.4, -4.7. LCMS (ESI) m/z: [M+H] calcd, 498.3; found, 498.3.

Compound 79. Intermediate **78** (5.12 g, 10.3 mmol) was co-evaporated with toluene (40 mL) under reduced pressure at 60 °C three times before being dissolved in dry pyridine (100 mL) and 2,6-lutidine (3.60 mL, 30.9 mmol). To this was added DMT-Cl (10.5 g, 30.9 mmol) and silver nitrate (3.50 g, 20.6 mmol) and the reaction was heated to 40 °C for 20 hours. After cooling in an ice bath, ethyl acetate and water was added, and the reaction mixture was transferred to a separatory funnel. The aqueous layer was extracted with ethyl acetate, and the organics were washed with saturated sodium bicarbonate solution and brine. The organics were concentrated to a crude yellow oil under reduced pressure. The curde product was purified by flash chromatography (silica gel, 220g col, 0-40% ethyl acetate/hexanes) to give **79** as a yellow foam. Yield: 5.53 g, (67.1 %). ^{1}H NMR (300 MHz, CHLOROFORM-d) δ 9.02 (d, *J*=17.03 Hz, 1H), 8.81 (d, *J*=19.07 Hz, 1H), 7.83-8.61 (m, 2H), 7.27-7.66 (m, 9H), 7.11-7.25 (m, 3H), 6.69-6.87 (m, 4H), 6.31-6.57 (m, 1H), 4.31-4.75 (m, 1H), 4.00-4.08 (m, 1H), 3.73-3.81 (m, 6H), 3.19-3.55 (m, 1H), 2.17-2.92 (m, 2H), 1.33-1.59 (m, 2H), 0.81-0.97 (m, 9H), 0.51-0.78 (m, 3H), 0.13 (d, *J*=12.93 Hz, 3H), -0.13--0.02 (m, 3H). ^{13}C NMR (75 MHz, CHLOROFORM-d) δ 171.2, 164.6, 158.6, 158.5, 158.5, 158.4, 152.6, 152.6, 151.6, 149.5, 149.4, 146.3, 146.2, 141.6, 141.5, 136.9, 136.9, 136.8, 136.7, 133.7, 132.7, 130.5, 130.5, 130.4, 130.3, 128.9, 128.8, 128.4, 128.3, 127.9, 127.6, 126.8, 126.7, 123.3, 113.0, 112.9, 88.6, 88.2, 86.6, 86.6, 84.6, 83.9, 75.7, 74.3,

73.5, 72.0, 60.4, 55.2, 55.2, 42.3, 40.4, 25.7, 25.7, 25.8, 23.6, 21.1, 17.9, 17.8, 14.2, 9.9, 9.4, -4.3, -4.5, -4.6, -4.7. LCMS (ESI) m/z: [M-H] calcd, 798.4; found, 798.5.

Compound 80. TEA (2.41 mL, 17.3 mmol) was added to a solution of **79** (5.53 g, 6.91 mmol) in THF (34.5 mL). The reaction was cooled in an ice bath under an atmosphere of nitrogen. Triethylamine trihydrofluoride (5.63 mL, 34.6 mmol) was added slowly and then the reaction warmed to room temperature and stirred for 14 hours. Further portions of triethylamine (1 mL) and triethylamine trihydrofluoride (2.25 mL) were added and after 4 more hours until no starting material remained (TLC). Triethylamine (5 ml) was added and the solvents were removed under reduced pressure to give a pale yellow solid. The crude material was purified by flash chromatography (silica gel, 220g col, 0-100% ethyl acetate/hexanes) gave the product as a white solid. Yield: 4.44 g (93.7 %). ^{1}H NMR (300 MHz, CHLOROFORM-d) δ 9.05 (d, *J*=17.03 Hz, 1H), 8.69-8.87 (m, 1H), 7.71 8.45 (m, 3H), 7.30-7.69 (m, 9H), 7.15-7.26 (m, 3H), 6.73-6.88 (m, 4H), 6.39 (td, *J*=6.70, 15.01 Hz, 1H), 4.39-4.75 (m, 1H), 3.84-4.06 (m, 1H), 3.74-3.83 (m, 6H), 3.41 (br s, 1H), 2.31-2.95 (m, 2H), 1.84-2.29 (m, 1H), 1.34-1.56 (m, 2H), 0.56-0.85 (m, 3H). ^{13}C NMR (75 MHz, CHLOROFORM-d) δ 171.2, 164.8, 158.6, 152.7, 152.6, 151.5, 151.4, 149.6, 149.5, 146.3, 146.2, 141.5, 141.3, 139.5, 136.8, 136.7, 136.5, 133.7, 132.8, 130.4, 129.2, 128.8, 128.3, 128.0, 127.9, 127.8, 127.7, 127.7, 130.4, 126.9, 126.9, 123.3, 123.1, 113.1, 113.1, 113.0, 87.5, 87.1, 86.8, 84.0, 83.9, 75.1, 74.7, 72.2, 71.2, 64.4, 60.4, 55.3, 55.2, 40.8, 39.9, 30.6, 24.3, 24.2, 21.1, 19.1, 14.2, 13.7, 10.3, 9.2. LCMS (ESI) m/z: [M+H] calcd, 686.3; found, 686.3.

Compound 81. Intermediate **80** (2.00 g, 2.92 mmol) was dissolved in dry DMF (29.2 mL) under an atmosphere of nitrogen. To this was added 1H-tetrazole (0.163 g, 2.33 mmol) and 1-methylimidazole (0.0578 mL, 0.729 mmol), followed by dropwise addition of 2-cyanoethyl-N,N,N',N'-tetraisopropyl-phosphorodiamidite (1.39 mL, 4.37 mmol). The reaction was stirred at room temperature for 90 minutes. Water (1 mL) was added to quench the reaction, and the reaction was diluted with a mixture of toluene/hexanes (3:1) The organics were washed four times with a mixture of DMF/H2O (3:2), followed by saturated sodium bicarbonate solution and brine. The organics were clooected, dried over sodium sulfate and concentrated to a white foam. The crude product was purified by flash chromatography (silica gel, 50g col, 80% ethyl acetate/hexanes) to give **81** as a white solid. Yield: 1.99 g (76.9 %). ^{1}H NMR (300 MHz,

CHLOROFORM-d) δ 9.12 (br s, 1H), 8.72-8.91 (m, 1H), 7.75-8.56 (m, 3H), 7.26-7.67 (m, 9H), 7.11-7.24 (m, 2H), 6.68-6.87 (m, 4H), 6.31-6.54 (m, 1H), 4.53-5.04 (m, 1H), 4.17-4.27 (m, 1H), 3.81-3.92 (m, 1H), 3.76-3.81 (m, 6H), 3.51-3.74 (m, 3H), 3.28-3.47 (m, 1H), 2.28-3.05 (m, 4H), 1.13-1.47 (m, 15H), 0.49-0.73 (m, 3H). ^{13}C NMR (75 MHz, CHLOROFORM-d) δ 164.6, 158.6, 158.6, 158.5, 158.4, 152.7, 152.6, 151.6, 149.5, 149.4, 146.4, 146.3, 146.3, 146.3, 141.6, 141.4, 141.3, 136.9, 136.8, 136.7, 136.7, 133.7, 132.8, 130.6, 130.5, 130.5, 130.4, 128.9, 128.9, 127.9, 128.0, 126.9, 126.8, 126.7, 123.5, 123.1, 117.5, 117.3, 113.0, 112.9, 112.9, 87.6, 87.5, 87.1, 87.1, 86.7, 86.7, 84.4, 84.2, 83.9, 83.7, 75.5, 75.3, 74.9, 74.8, 74.5, 73.6, 58.2, 58.3, 58.0, 58.0, 55.2, 55.2, 43.4, 43.4, 43.2, 43.2, 40.5, 39.2, 39.2, 24.7, 24.7, 24.6, 24.3, 24.0, 20.5, 20.4, 20.4, 20.3, 20.1, 20.0, 9.9, 9.8, 9.6, 9.6. ^{31}P NMR (121 MHz, CHLOROFORM-d) δ 149.38 (s, 1P), 149.30 (s, 1P), 148.88 (s, 1P), 148.24 (s, 1P). LCMS (ESI) m/z: [M-H] calcd, 884.5; found, 884.5.

Scheme S11. Synthesis of 5'-(S)-Allyl Thymidine Nucleosides.

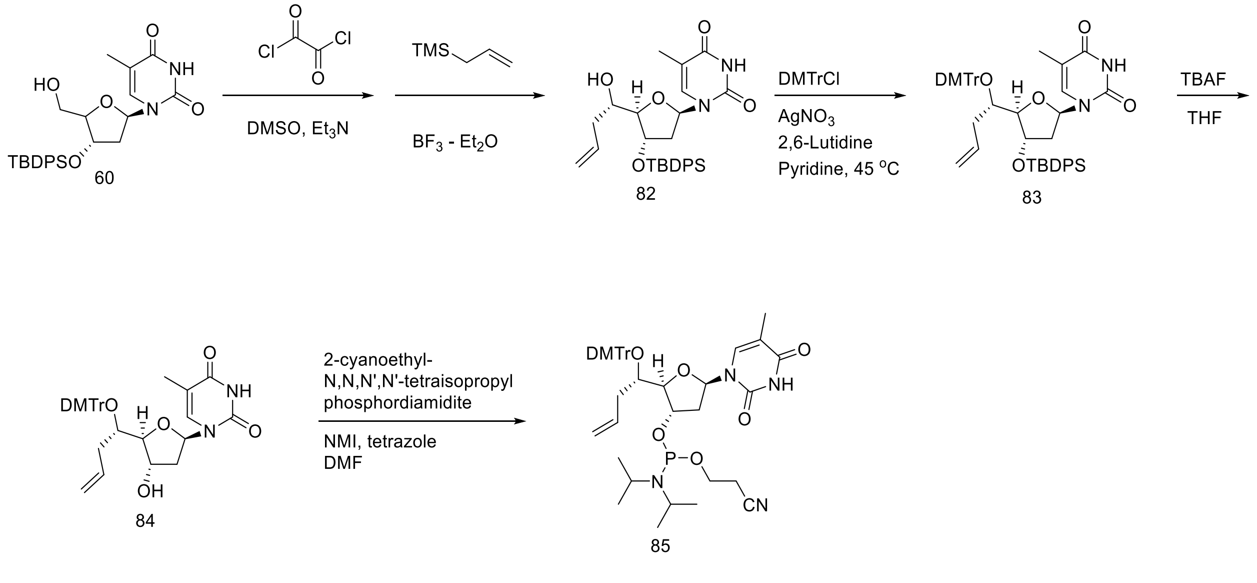

C**ompound 82.** Starting material **60** was dried over P_2O_5 for 16 hours prior to use. The starting material (12.8 g, 35.9 mmol) was dissolved in dry DCM (350 ml) and was cooled in an ice bath with stirring under nitrogen. TMS allyl(trimethyl)silane (28.5 ml, 180 mmol, 5 eq.) and BF_3-Et_2O (22.5 ml, 182 mmol, 5 eq.) was added dropwise via syringe and was allowed to stir for 1 hr at 0 °C under nitrogen. The reaction was monitored by TLC (2:1 EtOAc/hexanes. Aldehyde: Rf

= 0.30; product: Rf = 0.63). The reaction was quenched by slow addition of saturated aqueous $NaHCO_3$ (100 ml). The reaction was diluted with EtOAc (70 ml) and was transferred to a separatory funnel. The reaction mixture was washed with 1: 1 water and saturated aqueous $NaHCO_3$ (3 x 200 ml) followed by brine (1 x 200 ml). The organics were collected, dried over $MgSO_4$, filtered, and concentrated under reduced pressure. The crude product was purified by silica gel chromatography, using a gradient of 2% EtOAc in hexanes up to 70% EtOAc over 10 CV. Product fractions were pooled and concentrated to a white brittle foam. Yield: 9.1 g (64%). ^{1}H NMR (300MHz, CHLOROFORM-d) δ = 8.69 (s, 1H), 7.57 - 7.43 (m, 1H), 6.18 (dd, *J*=6.5, 7.2 Hz, 1H), 5.93 - 5.78 (m, 1H), 5.25 - 5.13 (m, 2H), 4.50 (td, *J*=3.2, 6.2 Hz, 1H), 3.90 - 3.70 (m, 2H), 2.49 (d, *J*=5.2 Hz, 1H), 2.42 - 2.28 (m, 3H), 2.26 - 2.11 (m, 1H), 2.05 (s, 1H), 1.97 - 1.85 (m, 3H), 1.26 (t, *J*=7.1 Hz, 1H), 0.92 - 0.83 (m, 10H), 0.16 - -0.01 (m, 6H). LCMS (ESI) m/z: [M+H] calcd, 396.6; found, 397.2.

Note: (S)-5'-allyl T 3'-O-TBDMS has been reported in the Escudier paper: Catana, D. A., Renard, B. L., Maturano, M., Payrastre, C., Tarrat, N., & Escudier, J. M. (2012). Dioxaphosphorinane-constrained nucleic Acid dinucleotides as tools for structural tuning of nucleic acids. *Journal of nucleic acids*, *2012*, 215876. https://doi.org/10.1155/2012/215876

C**ompound 83.** Intermediate **82** was dried over P_2O_5 for 16 hours under high vacuum prior to use. 5'-allyl dT 3'-O-TBDPS (1.35 g, 2.59 mmol) was dissolved in pyridine (20 ml) and lutidine (0.9 ml ,7.78 mmol, 3 eq.). DMT-Cl (2.70 g, 8 mmol, 3 eq.) was added to the reaction, followed by $AgNO_3$ (132 mg, 0.78 mmol, 0.3 eq.). The reaction was purged with argon, capped tightly, and was allowed to stir at 40 °C for 4 days. The reaction was monitored by TLC (5% MeOH in DCM). Upon completion, the reaction was cooled to room temperature, diluted with EtOAc (50 ml), and was filtered through a plug of celite to remove silver salts. The filtrate was transferred to a separatory funnel and washed with saturated aqueous $NaHCO_3$ (2 x 25 ml) and brine (1 x 25 ml). The organics were collected, dried over $MgSO_4$, filtered, and concentrated under reduced pressure. The crudes material was purified by silica gel chromatography, neutralize with 5% triethylamine in hexanes, and purified using an EtOAc/hexanes gradient. Yield: 7.0 g (87 %). ^{1}H NMR (300MHz, CHLOROFORM-d) δ = 8.46 (s, 1H), 7.93 (d, *J*=1.2 Hz, 1H), 7.54 - 7.30 (m, 9H), 7.26 - 7.06 (m, 12H), 6.86 - 6.60 (m, 5H), 6.51 (dd, *J*=5.4, 8.9 Hz, 1H), 5.09 - 4.95 (m, 1H),

4.85 - 4.74 (m, 2H), 4.37 (d, *J*=5.9 Hz, 1H), 4.13 (q, *J*=7.1 Hz, 1H), 3.96 (t, *J*=1.5 Hz, 1H), 3.78 (d, *J*=1.5 Hz, 7H), 3.04 - 2.97 (m, 1H), 2.37 - 1.96 (m, 6H), 1.74 (d, *J*=1.0 Hz, 3H), 1.70 - 1.57 (m, 1H), 1.32 - 0.98 (m, 12H), 0.95 - 0.73 (m, 1H), 0.03 - -0.02 (m, 1H). ^{13}C NMR (75MHz, CHLOROFORM-d) δ = 171.10, 163.60, 158.64, 158.57, 150.15, 145.72, 136.25, 135.84, 135.82, 135.63, 135.47, 133.51, 133.21, 133.15, 130.32, 130.16, 129.82, 129.74, 128.12, 127.71, 127.61, 126.97, 117.77, 112.97, 111.15, 87.73, 87.19, 84.62, 77.20, 75.02, 74.77, 60.36, 55.20, 41.70, 36.24, 26.79. LCMS (ESI) m/z: [M+Na] calcd, 823.1; found, 846.0

C**ompound 84.** Intermediate **83** (1.88 g, 2.28 mmol) was dissolved in dry THF (20 ml). A solution of TBAF was added (1 M in THF, 3.43 ml, 3.43 mmol, 1.5 eq.) and the reaction was stirred at room temperature for 16 hours under nitrogen. Upon completion, the solvent was removed under reduced pressure. The crude material was dissolved in EtOAc (100 ml) and was transferred to a separatory funnel. The reaction was washed with saturated aqueous $NaHCO_3$ (50 ml), water (50 ml) and brine (50 ml). The organics were collected, dried over $MgSO_4$, filtered, and concentrated under reduced pressure. The crude material was purified by silica gel chromatography using a MeOH/DCM gradient. Yield: 1.27 grams (95 %). ^{1}H NMR (300MHz, CHLOROFORM-d) δ = 8.93 (br s, 1H), 7.87 (d, *J*=1.2 Hz, 1H), 7.48 - 7.19 (m, 10H), 6.91 - 6.75 (m, 4H), 6.33 (t, *J*=6.8 Hz, 1H), 5.48 - 5.33 (m, 1H), 4.95 - 4.84 (m, 2H), 4.42 - 4.34 (m, 1H), 4.12 (q, *J*=7.2 Hz, 1H), 3.80 (d, *J*=1.8 Hz, 7H), 3.48 - 3.34 (m, 1H), 2.45 - 2.11 (m, 5H), 2.09 - 2.00 (m, 1H), 1.83 (d, *J*=1.0 Hz, 4H), 1.26 (t, *J*=7.1 Hz, 1H), 0.02 - -0.08 (m, 1H). ^{13}C NMR (75MHz, DMSO-d_6) δ = 169.38, 140.62, 140.51, 139.27, 139.10, 139.08, 139.02, 135.92, 135.84, 135.80, 132.62, 132.58, 131.76, 131.32, 128.39, 126.62, 59.68, 45.94, 38.44, 38.32, 38.15, 30.70, 30.57, 32.37, 29.47, 28.56, 28.03, 27.77, 24.05, 23.73, 21.84, 20.65, 14.00, 13.76, 13.73. LCMS (ESI) m/z: [M-H] calcd, 584.7; found, 584.0.

C**ompound 85.** Intermediate **84** (4.14 g, 7.08 mmol) and tetrazole (400 mg, 5.66 mmol, 0.8 eq.) were dried over P_2O_5 for 16 hours under high vacuum. The reagents were dissolved in dry DMF (710 ml) with stirring under nitrogen. N-methylimidazole (140 µl, 1.77 mmol, 0.25 eq.) and 2-cyanoethyl-N,N,N',N'-tetraisopropyl-phosphorodiamidite (3.37 ml, 10.6 mmol, 1.5 eq.) were added via syringe and the reaction was allowed to stir at room temperature under nitrogen for 4

hours. The reaction was diluted with EtOAc (200 ml) and triethylamine (1 ml) and was transferred to a separatory funnel. The reaction was washed with saturated aqueous $NaHCO_3$(200 ml), water (200 ml) and brine (200 ml). The organics were collected, dried over $MgSO_4$, filtered, and concentrated under reduced pressure. The crude material was purified by dissolving in a minimum amount of DCM and precipitating by slow addition into hexanes. The product was dried under high vacuum to give the desired product as a brittle foam. Yield: 4.01 g (74%). ^{1}H NMR (300MHz, CHLOROFORM-d) δ = 8.65 (br s, 1H), 7.97 (dd, J=1.2, 12.9 Hz, 1H), 7.48 - 7.28 (m, 7H), 7.26 - 7.20 (m, 1H), 6.84 (td, J=3.3, 8.9 Hz, 4H), 6.43 - 6.29 (m, 1H), 5.43 - 5.23 (m, 1H), 4.96 - 4.81 (m, 2H), 4.49 (dquin, J=3.0, 7.0 Hz, 1H), 4.01 (br d, J=9.9 Hz, 1H), 3.83 - 3.65 (m, 6H), 3.63 - 3.44 (m, 3H), 3.40 - 3.31 (m, 1H), 2.62 - 2.26 (m, 5H), 2.24 - 2.13 (m, 1H), 1.80 - 1.74 (m, 3H), 1.68 (s, 1H), 1.37 - 1.09 (m, 10H), 0.97 (d, J=6.8 Hz, 3H), 0.10 - -0.06 (m, 1H). ^{13}C NMR (75MHz, CHLOROFORM-d) δ = 163.66, 163.64, 158.78, 158.77, 158.75, 150.25, 150.15, 145.93, 145.86, 136.46, 136.41, 136.19, 136.07, 135.60, 135.52, 133.69, 130.45, 130.41, 128.27, 127.77, 130.35, 117.98, 117.46, 117.20, 113.11, 113.08, 111.34, 111.21, 87.31, 87.25, 86.47, 86.45, 86.42, 86.37, 84.30, 84.23, 77.20, 74.30, 73.95, 73.99 (br dd, J=18.4, 83.7 Hz, 1C), 58.14, 55.28, 55.23, 43.25, 40.49, 40.43, 36.23, 24.61, 24.56, 24.44 (dd, J=2.2, 7.7 Hz, 1C), 20.35, 20.26, 20.02, 19.93, 12.21. ^{31}P NMR (121MHz, CHLOROFORM-d) δ = 149.54 (s, 1P), 149.10 (s, 1P). LCMS (ESI) m/z: [M-H] calcd, 784.9; found, 783.5.

Scheme S12. Synthesis of 5'-(R)-Allyl Thymidine Nucleosides.

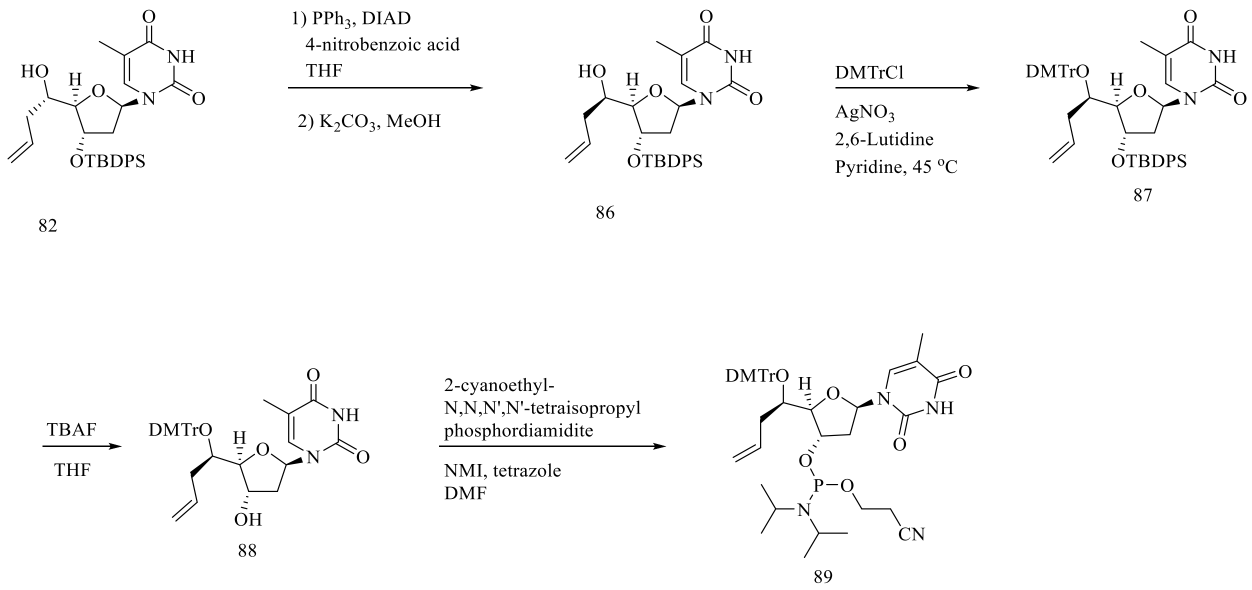

82 86 87 88 89

Compound 86. (S) 5'-allyl dT 3'-O-TBDPS (**82**, 1.83 g, 3.51 mmol) and PPh_3 (3.69 grams, 14.1 mmol, 4 eq.) was dissolved in dry THF (46 ml) with stirring under nitrogen. DIAD (2.72 ml, 14.1 mmol, 4 eq.) was added dropwise via syringe, and the reaction was allowed to stir at room temperature for 16 hours. Upon completion, the solvent was removed under reduced pressure. The residue was resuspended in DCM (50 ml) and was transferred to a separatory funnel. The reaction mixture was washed with saturated aqueous $NaHCO_3$, dried over $MgSO_4$, and concentrated under reduced pressure. The crude product was purified by silica gel chromatography (flash chromatography) using a DCM/EtOAc gradient. Yield: 2.3 grams (98 %). ^{1}H NMR (300MHz, CHLOROFORM-d) δ = 8.39 - 8.18 (m, 3H), 8.13 - 7.79 (m, 3H), 7.69 - 7.57 (m, 5H), 7.49 - 7.30 (m, 8H), 6.55 - 6.24 (m, 6H), 5.10 - 4.85 (m, 8H), 4.33 - 4.07 (m, 2H), 2.43 - 2.16 (m, 4H), 1.93 - 1.67 (m, 7H), 1.35 - 0.98 (m, 47H), 0.07 - -0.07 (m, 2H). LCMS (ESI) m/z: [M+H] calcd, 669.8; found, 670.2.

The purified intermediate (2.3 grams, 3.43 mmol) was dissolved in MeOH (35 ml). K_2CO_3 was added (1.42 grams, 10.3 mmol, 3 eq.), and the reaction was stirred vigorously overnight at room temperature. The solvent was removed under reduced pressure. The residue was resuspended in EtOAc (50 ml) and was transferred to a separatory funnel. The reaction was washed with water, followed by brine (50 ml each), dried over MgSO4, and concentrated under reduced pressure. The crude product was purified by silica gel chromatography using a DCM/MeOH gradient. Product fractions were concentrated and dried under high vacuum to give **86** as a white brittle foam. Yield: 1.42 grams (79 %). LCMS (ESI) m/z: [M+H] calcd, 396.6; found, 395.2.

Compound 87. Intermediate **86** (1.42 g, 2.73 mmol) was dissolved in pyridine (15 ml) and lutidine (0.95 ml , 8.18 mmol, 3 eq.). DMT-Cl (2.77 g, 8.18 mmol, 3 eq.) was added to the reaction followed by $AgNO_3$ (100 mg, 0.59 mmol, 0.072 eq.). The reaction was purged with argon, capped tightly, and was allowed to stir at 45 °C for 4 days. The reaction was monitored by TLC (5% MeOH in DCM). Upon completion, the reaction was cooled to room temperature, diluted with EtOAc (50 ml), and was filtered through a plug of celite to remove silver salts. The filtrate was transferred to a separatory funnel and washed with saturated aqueous $NaHCO_3$ (2 x 25 ml) and brine (1 x 25 ml). The organics were collected, dried over $MgSO_4$, filtered, and concentrated under reduced pressure. The crude material was purified by silica gel

chromatography (flash chromatography) and purified using an EtOAc/hexanes gradient. Product fractions were pooled and dried under high vacuum to give the desired product as a yellow brittle foam. Yield: 1.33 g (60 %). ^{1}H NMR (300MHz, CHLOROFORM-d) δ = 8.27 (s, 1H), 7.78 - 7.64 (m, 4H), 7.55 - 7.33 (m, 8H), 7.33 - 7.28 (m, 1H), 7.26 - 7.06 (m, 10H), 6.82 (d, J=1.2 Hz, 2H), 6.70 (dd, J=6.1, 9.0 Hz, 5H), 4.13 (d, J=7.0 Hz, 2H), 3.81 - 3.72 (m, 9H), 2.05 (s, 4H), 1.73 (d, J=1.0 Hz, 2H), 1.65 (s, 3H), 1.42 (d, J=1.0 Hz, 3H), 1.31 - 1.23 (m, 6H), 1.10 (s, 9H), 0.99 (s, 3H), 0.01 (s, 2H). ^{13}C NMR (75MHz, CHLOROFORM-d) δ = 163.32, 158.58, 158.55, 150.04, 146.06, 136.62, 136.21, 136.01, 135.97, 135.63, 135.47, 135.39, 133.97, 133.28, 133.13, 130.31, 130.20, 130.15, 130.11, 130.02, 128.12, 128.01, 127.85, 127.73, 127.61, 126.82, 117.45, 113.10, 113.07, 112.97, 110.80, 87.95, 86.83, 83.80, 77.20, 74.27, 72.91, 70.19, 60.36, 55.20, 40.08, 35.40, 26.90, 26.79, 21.93, 21.01, 19.03, 18.87, 14.18, 11.76. LCMS (ESI) m/z: [M-H] calcd, 823.1; found, 821.9.

C**ompound 88.** Starting material **87** (1.33 g, 1.62 mmol) was dissolved in dry THF (16 ml). A solution of TBAF was added (1 M in THF, 2.42 ml, 2.42 mmol, 1.5 eq.) and the reaction was stirred at room temperature overnight under nitrogen. Upon completion, the solvent was removed under reduced pressure. The crude material was dissolved in EtOAc (100 ml) and was transferred to a separatory funnel. The reaction was washed with saturated aqueous $NaHCO_3$ (150 ml), water (150 ml), and brine (150 ml). The organics were collected, dried over $MgSO_4$, filtered, and concentrated under reduced pressure. The crude material was purified by silica gel chromatography using an EtOAc/hexanes gradient. Yield: 760 mg (80 %). ^{1}H NMR (300MHz, CHLOROFORM-d) δ = 8.90 (s, 1H), 7.52 - 7.35 (m, 6H), 7.33 - 7.28 (m, 1H), 7.26 - 7.17 (m, 2H), 6.97 (d, J=1.2 Hz, 1H), 6.89 - 6.79 (m, 4H), 6.28 (dd, J=5.6, 8.4 Hz, 1H), 5.30 (s, 1H), 4.99 - 4.70 (m, 3H), 3.91 (t, J=3.4 Hz, 1H), 3.83 - 3.75 (m, 6H), 3.36 (s, 1H), 2.37 - 2.12 (m, 4H), 2.05 (s, 1H), 1.79 (s, 1H), 1.47 (d, J=1.0 Hz, 3H), 1.38 - 1.15 (m, 1H), 0.01 (s, 1H). ^{13}C NMR (75MHz, CHLOROFORM-d) δ = 163.54, 158.76, 158.72, 150.26, 146.22, 136.63, 136.35, 135.31, 133.95, 130.25, 130.21, 128.00, 127.87, 127.02, 117.88, 113.24, 113.21, 113.16, 111.07, 87.20, 86.96, 83.55, 74.13, 71.08, 55.22, 40.61, 36.35, 11.86. LCMS (ESI) m/z: [M-H] calcd, 584.7; found, 584.0.

C**ompound 89.** Starting material (**88**, 260 mg, 0.54 mmol) and tetrazole (24.9 mg, 0.356 mmol, 0.8 eq.) were dried over P_2O_5 for 16 hours under high vacuum. The reagents were dissolved in dry DMF (9 ml) with stirring under nitrogen. N-methylimidazole (8.8 µl, 0.111 mmol, 0.25 eq.) and 2-cyanoethyl-N,N,N',N'-tetraisopropyl-phosphorodiamidite (212 µl, 0.667 mmol, 1.5 eq.) were added via syringe and the reaction was allowed to stir at room temperature under nitrogen for 12 hours. The reaction was diluted with EtOAc (50 ml) and triethylamine (1 ml) and was transferred to a separatory funnel. The reaction was washed with saturated aqueous $NaHCO_3$ (50 ml), water (50 ml), and brine (50 ml). The organics were collected, dried over $MgSO_4$, filtered, and concentrated under reduced pressure. The crude material was purified by silica gel chromatography (flash chromatography) using an EtOAc/hexanes gradient. The product was dried under high vacuum to give the desired product as a brittle foam. Yield: 276 mg (79%). ^{1}H NMR (300MHz, CHLOROFORM-d) δ = 7.60 - 7.13 (m, 7H), 7.03 (dd, J=1.2, 6.9 Hz, 1H), 6.89 - 6.74 (m, 1H), 8.54 - 6.59 (m, 7H), 6.35 - 6.19 (m, 1H), 6.40 - 4.59 (m, 1H), 4.45 - 3.14 (m, 1H), 2.96 - 0.88 (m, 2H). ^{13}C NMR (75MHz, CHLOROFORM-d) δ = 163.41, 158.74, 158.71, 158.68, 150.19, 150.12, 146.30, 136.76, 136.72, 136.41, 136.25, 135.55, 135.43, 133.90, 133.81, 130.27, 130.22, 130.15, 128.06, 127.97, 127.87, 127.85, 126.98, 126.93, 117.71, 117.60, 117.39, 113.22 (t, J=2.7 Hz, 1C), 113.10, 113.07, 111.04, 111.01, 86.96, 86.91, 86.44, 86.33, 83.57, 83.41, 76.26, 74.37, 74.00, 72.65 (br dd, J=16.5, 73.0 Hz, 1C), 58.52, 58.28, 57.88, 57.62, 55.22, 43.48, 43.36, 43.31, 43.20, 39.51, 39.47, 36.02, 35.96, 35.93, 24.73, 24.66, 24.60, 24.57 (dd, J=3.3, 6.0 Hz, 1C), 24.47, 24.45, 20.53, 20.41, 20.31, 14.17, 11.67, 11.62. ^{31}P NMR (121MHz, CHLOROFORM-d) δ = 148.71 (s, 1P), 147.79 (s, 1P). LCMS (ESI) m/z: [M-H] calcd, 784.9; found, 783.5

Scheme S13. Synthesis of 5'-(R/S)-Allyl Adenosine Nucleosides.

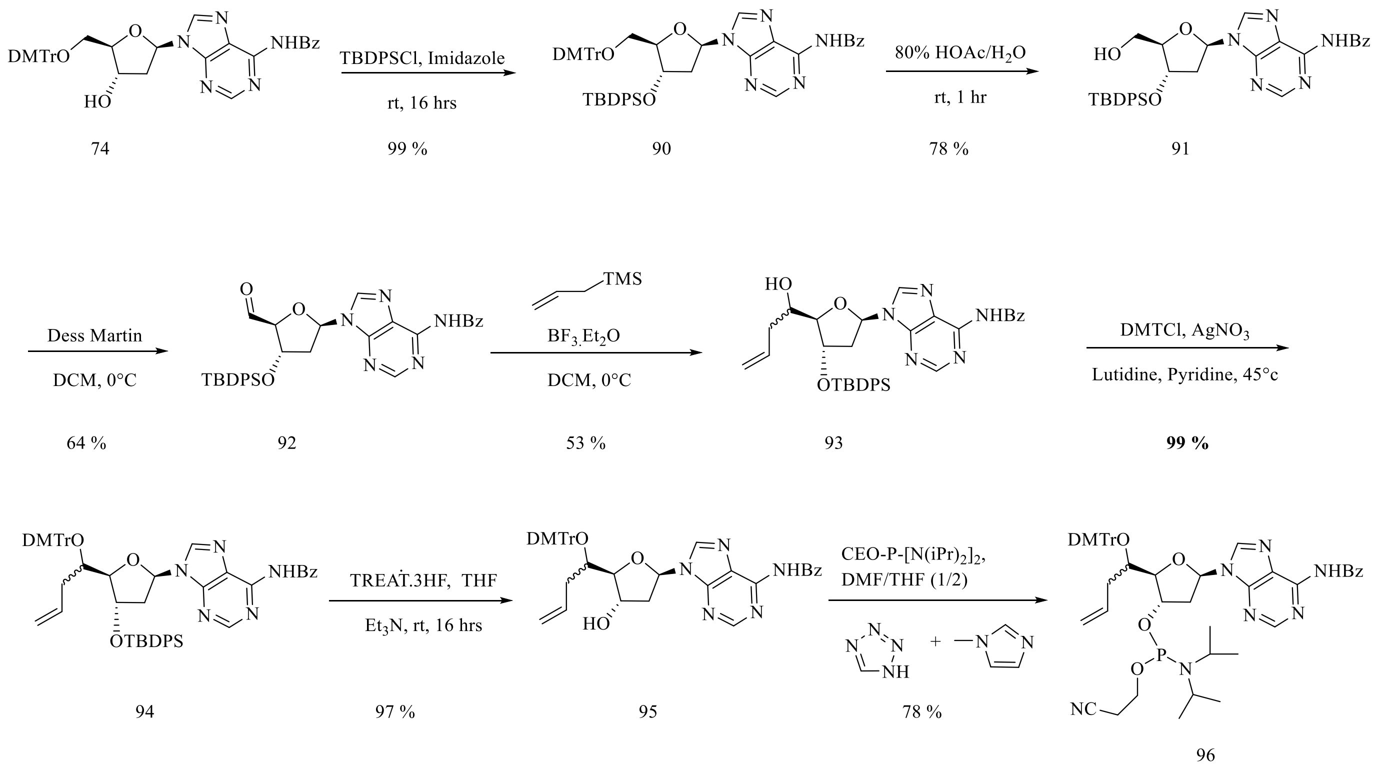

Compound 90. Protected nucleoside **74** (20 g, 29.10 mmol,) and imidazole (8.0 g, 118 mmol, 4 eq) were dissolved in anhydrous DMF (50 mL), and the solution was cooled with ice bath under an atmosphere of nitrogen. A solution of tert-butyl-chloro-dimethyl-silane (8.0 g, 29.10 mol 1.0 eq) in DMF (20 mL) was added dropwise. The reaction was stirred for 3 hours at room temperature. TLC in EtOAc/hexanes (7/3) indicated reaction was completed. The reaction was slowly poured in 100 mL of ice with stirring to obtain a white precipitate, stirring was continued until all the ice melted. The solid was collected by filtration, and washed with water (3x50 mL) and hexanes (3x50 mL). The solid was collected and dried under high vacuum at 35°C to obtain the desired product (**90**) as a white solid. Yield: 29 g (99 %). ^{1}H NMR (300MHz, CHLOROFORM-d) δ = 9.03 (s, 1H), 8.71 (s, 1H), 8.07 - 7.97 (m, 3H), 7.72 - 7.56 (m, 6H), 7.53 - 7.46 (m, 2H), 7.44 - 7.11 (m, 18H), 6.70 (dd, *J*=1.6, 8.8 Hz, 4H), 6.55 (t, *J*=6.6 Hz, 1H), 4.68 - 4.61 (m, 1H), 4.28 (br d, *J*=2.7 Hz, 1H), 3.75 (s, 6H), 3.20 (dd, *J*=4.1, 10.3 Hz, 1H), 3.03 (dd, *J*=4.5, 10.4 Hz, 1H), 2.60 (br d, *J*=7.4 Hz, 1H), 2.51 (br s, 1H), 1.75 (br s, 2H), 1.19 - 0.97 (m,

12H). ^{13}C NMR (75MHz, CHLOROFORM-d) δ = 164.52, 158.43, 152.60, 149.39, 144.44, 141.42, 135.72, 135.58, 135.25, 134.81, 133.75, 133.14, 132.99, 132.73, 130.04, 129.98, 129.96, 129.62, 128.86, 128.08, 127.88, 127.84, 127.79, 127.71, 126.82, 123.25, 113.07, 87.11, 86.40, 84.83, 73.95, 63.44, 55.22, 40.56, 26.91, 26.58, 19.08, 19.03. LCMS (ESI) m/z: [M-H] calcd, 771.4; found, 771.3

Compound 91. Intermediate **90** (26 g, 29.20 mmol,) was dissolved in 80% acetic acid/water (156 mL) and stirred for 1 hour at room temperature. TLC in EtOAc/hexanes (9/1) indicated the reaction was completed. The solvents were evaporated under reduce pressure to obtain an oil. The crude material was dissolved in ethyl acetate (100 mL), washed with saturated $NaHCO_3$ an d brine. The organics were dried over Na_2SO_4,filtered, and concentrated under reduced pressure. The crude material was purified by flash chromatography (silica gel, 350g col, 60 % diethyl ether/hexanes, 50-100 % EtOAc/hexanes) to afford the desired product (**91**) as a white solid. Yield: 13.6 g (78 %). ^{1}H NMR (300MHz, CHLOROFORM-d) δ = 9.20 (s, 1H), 8.69 (s, 1H), 8.10 - 7.96 (m, 3H), 7.69 - 7.37 (m, 13H), 7.26 (s, 1H), 6.43 (dd, *J*=5.3, 9.5 Hz, 1H), 5.62 (br d, *J*=10.9 Hz, 1H), 4.74 (d, *J*=4.8 Hz, 1H), 4.16 (s, 1H), 3.72 (br d, *J*=12.7 Hz, 1H), 3.21 (br t, *J*=11.6 Hz, 1H), 3.00 - 2.82 (m, 1H), 2.31 (dd, *J*=5.3, 13.1 Hz, 1H), 2.04 (br s, 1H), 1.26 (s, 1H), 1.13 (s, 9H). ^{13}C NMR (75MHz, CHLOROFORM-d) δ = 164.57, 152.03, 150.70, 150.24, 142.59, 135.73, 135.68, 133.47, 133.24, 133.10, 132.90, 130.19, 130.15, 128.87, 127.96, 127.92, 124.56, 90.28, 88.00, 74.94, 62.95, 41.14, 26.98, 19.08. LCMS (ESI) m/z: [M-H] calcd, 469.3; found, 468.1

Compound 92. A suspension of **91** (13.2 g, 22.3 mmol,) in anhydrous dichloromethane (220 mL) was cooled with an ice bath under an atmosphere of nitrogen. To this was added Dess-Martin periodinane (14.20 g, 33.4 mmol, 1.5 eq) and the reaction was stirred for 3 hours. TLC in EtOAc/hexanes indicated reaction was complete. The reaction was quenched by slow addition of 1.0 M $Na_2S_2O_3$ (175.0 mL) and 1.0 M $NaHSO_4$ (175 mL) and then stirred for a further 10 minutes. The reaction mixture was filtered through celite and rinsed with dichloromethane. The solution was transferred to a separatory funnel and the aqueous layer was discarded. The organics were dried over Na_2SO_4 for 10 minutes, filtered and concentrated under reduce pressure

to obtain a colorless oil. The oil was dissolved in a minimum amount of EtOAc and slowly added to hexanes (500 mL) with stirring to obtained white precipitate. The solid was filtered and dried under high vacuum over P_2O_5 to give the product. Yield: 8.40 g (64 %). ^{1}H NMR (300MHz, CHLOROFORM-d) δ = 9.39 (s, 1H), 8.80 - 8.65 (m, 1H), 8.35 (s, 1H), 8.12 (s, 1H), 8.08 - 7.94 (m, 4H), 7.73 - 7.37 (m, 21H), 6.70 (s, 1H), 4.93 - 4.74 (m, 1H), 4.60 (s, 1H), 4.56 - 4.49 (m, 1H), 4.25 (s, 1H), 2.57 (td, *J*=3.3, 5.5 Hz, 1H), 1.26 (s, 1H), 1.21 - 1.06 (m, 15H). ^{13}C NMR (75MHz, CHLOROFORM-d) δ = 198.95, 141.97, 135.83, 135.78, 135.69, 132.84, 132.52, 130.43, 130.37, 130.30, 128.93, 128.88, 128.13, 128.09, 128.05, 128.02, 127.95, 91.50, 86.49, 73.90, 39.82, 26.96, 26.88, 19.10, 19.08. LCMS (ESI) m/z: [M-H] calcd, 467.2; found, 466.0

Compound 93. Intermediate **92** (8.0 g, 13.51 mmol) was dissolved in anhydrous dichloromethane (130 mL) under nitrogen and was cooled in an ice bath. Allyltrimethylsilane (10.6 mL, 67.60 mmol, 5 eq) was added, followed by boron trifluoride diethyl etherate (8.33 mL, 67.5 mmol, 5 eq). The reaction was stirred at 0°C for 2 hours and was monitored by TLC (EtOAc/hexanes 9:1). Upon completion the reaction was quenched by slowly adding saturated $NaHCO_3$ solution (50 mL). The reaction was transferred to a separatory funnel and the aqueous layer was discarded. The organics were washed with water (100 mL), and brine. The organics were collected, dried over Na_2SO_4, filtered and concentrated under reduced pressure. The crude product was purified by flash chromatography (50 g col, 30-60% hexanes/EtOAc) afforded the desired product. Yield: 4.5 g (53 %). ^{1}H NMR (300MHz, CHLOROFORM-d) δ = 8.95 (s, 1H), 8.72 (s, 1H), 8.11 (s, 1H), 8.01 (d, *J*=7.4 Hz, 2H), 7.70 - 7.37 (m, 17H), 4.04 (s, 1H), 1.55 (s, 29H), 1.25 (s, 5H), 1.21 - 1.00 (m, 13H). LCMS (ESI) m/z: [M-H] calcd, 509.3; found, 508.2

Compound 94. Intermediate **93** (1 g, 1.6 mmol) was coevaporated with toluene (3x20 mL) under reduced pressure at 60°C before being dissolved in dry pyridine (10 mL) and 2,6-lutidine (0.52 mL, 4.40 mmol, 3 eq). To this was added DMT-Cl (2.0 g, 3 mmol, 4 eq) and silver nitrate (0.54 g, 20.62, 2.8 mmol, 2 eq) and the reaction was heated to 45°C for 16 hours. The reaction was monitored by TLC (EtOAc/hexanes 6:4). Upon completion, the reaction was cooled to 0 °C and was quenched by the addition of ethyl acetate and water. The aqueous layer was removed

and the organics were washed with saturated sodium bicarbonate solution and brine. The organics were dried over Na_2SO_3, filtered, and concentrated to a crude yellow oil under reduced pressure. The crude product was purified by flash chromatography (silica gel, 220g col, 0-50% ethyl acetate/hexanes) to give **94** as yellow foam. Yield: 1.50 g (99 %). ^{1}H NMR (300MHz, CHLOROFORM-d) δ = 9.01 (s, 1H), 8.85 (d, *J*=3.1 Hz, 1H), 8.54 (d, *J*=11.8 Hz, 1H), 8.05 (br d, *J*=8.3 Hz, 2H), 7.55 (br d, *J*=7.5 Hz, 4H), 7.47 - 7.28 (m, 7H), 7.25 - 7.04 (m, 6H), 6.91 - 6.71 (m, 3H), 6.61 (s, 3H), 3.81 - 3.71 (m, 7H), 1.60 (s, 6H), 1.26 (s, 2H), 1.03 (s, 4H), 0.93 (s, 2H), 0.83 (s, 4H), 0.19 - 0.05 (m, 2H). ^{13}C NMR (75MHz, CHLOROFORM-d) δ = 158.57, 151.68, 135.68, 135.57, 134.05, 132.79, 130.40, 130.32, 130.24, 129.86, 128.91, 128.26, 128.14, 127.84, 127.80, 127.70, 127.57, 113.17, 113.04, 113.00, 112.92, 112.88, 73.77, 55.23, 55.19, 26.87, 25.74, 17.84. LCMS (ESI) m/z: [M-H] calcd, 811.4; found, 810.3

Compound 95. TEA (0.372 mL, 2.5 eq, 2.67 mmol, 2.5 eq) was added to a solution of intermediate **94** (1.0 g, 1.07 mmol) in THF (10 mL). The reaction was cooled in an ice bath under an atmosphere of nitrogen. Triethylamine trihydrofluoride (0.871 mL, 5.34 mmol, 5.0 eq) was added slowly and then stirred at room temperature for 16 hours. Triethylamine (0.40 mL) was added and the solvent was removed under reduced pressure to give a pale yellow solid. The crude product was purified by flash chromatography (silica gel, 220g col, 50-100% ethyl acetate/hexanes) to give **95** as a white solid. Yield: 0.72 g (97 %). ^{1}H NMR (300MHz, CHLOROFORM-d) δ = 8.97 (s, 1H), 8.81 (s, 1H), 8.35 (s, 1H), 8.04 (br d, *J*=7.8 Hz, 2H), 7.55 (br d, *J*=7.7 Hz, 3H), 7.48 - 7.30(m, 6H), 7.24 - 7.15 (m, 2H), 6.86 - 6.76 (m, 4H), 4.94 (br s, 2H), 3.84 - 3.60 (m, 8H), 1.58 (s, 9H), 1.26 (s, 2H). LCMS (ESI) m/z: [M-H] calcd, 697.3; found, 696.2

Compound 96. Precursor **95** (0.70 g, 1.0 mmol) was dissolved in dry DMF (20 mL) under an atmosphere of nitrogen. To this was added 1H-tetrazole (55 mg, 0.80 mmol, 0.8 eq) and 1-methylimidazole (19 µL, 0.251 mmol, 0.25 eq), followed by dropwise addition of 2-cyanoethyl-N,N,N',N'-tetraisopropyl-phosphorodiamidite (0.478 mL, 1.5 mmol, 1.5 eq). The reaction was stirred at room temperature for 2 hours. Water (1 mL) was added to quench the reaction. The reaction was diluted with a mixture of toluene/hexanes (3:1). The organics were washed four

times with a mixture of DMF/H_2O (3:2). The organics were then washed with saturated sodium bicarbonate solution and brine. The organics were collected and dried over sodium sulfate, filtered, and concentrated to a white foam. The crude product was purified by flash chromatography (silica gel, 25 g col, 50-80% EtOAc/hexanes + 1 % triethyl amine) to give the desired product (**96**) as a white solid. Yield: 0.70 g (78 %). ^{1}H NMR (300MHz, CHLOROFORM-d) δ = 8.82 (s, 1H), 8.51 - 8.46 (m, 1H), 8.05 (d, *J*=7.6 Hz, 2H), 7.62 - 7.17 (m, 15H), 6.84 - 6.74 (m, 4H), 4.90 (s, 2H), 4.16 (br s, 1H), 3.88 - 3.68 (m, 8H), 3.68 - 3.45 (m, 5H), 2.77 - 2.52 (m, 3H), 2.36 - 2.18 (m, 2H), 2.05 (s, 2H), 1.73 - 1.64 (m, 3H), 1.29 - 1.12 (m, 19H), 1.05 (d, *J*=6.8 Hz, 3H). ^{31}P NMR (121MHz, CHLOROFORM-d) δ = 149.43 (s, 1P), 149.34 (s, 1P), 148.83 (s, 1P), 148.02 (s, 1P), 123.65 (s, 1P). LCMS (ESI) m/z: [M-H] calcd, 897.4; found, 896.4.

Scheme S14. Synthesis of 5'-(R/S)-Allyl Guanosine Nucleosides.

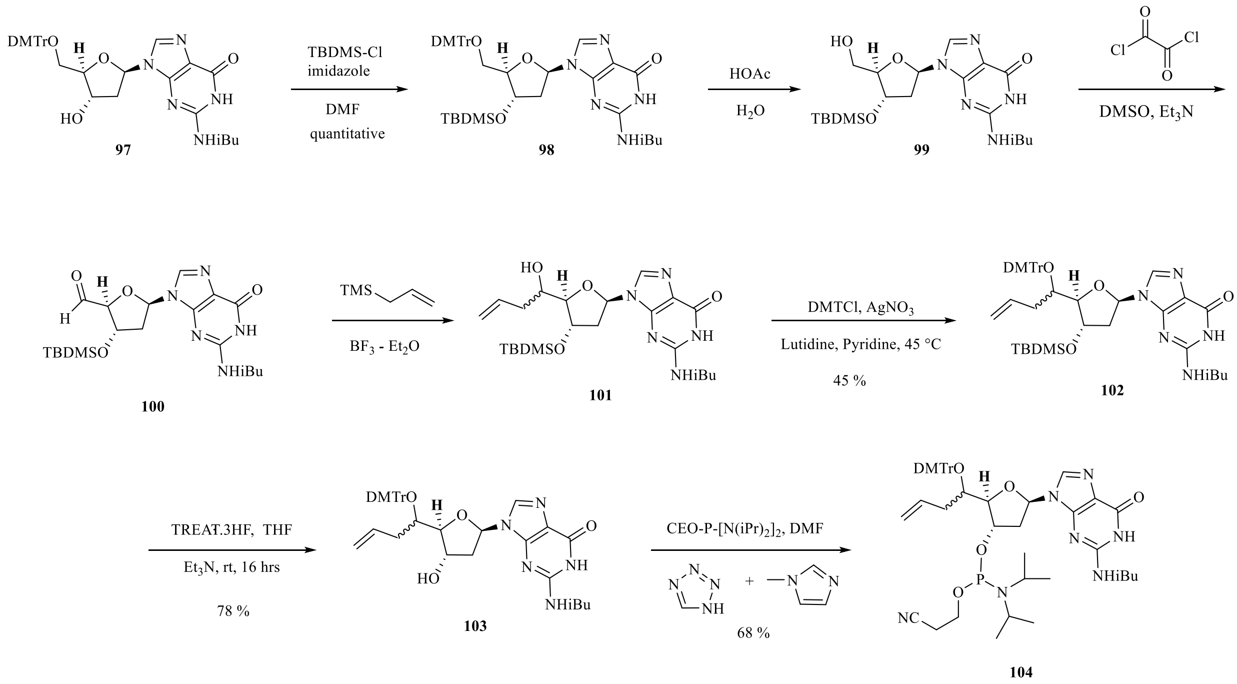

Compound 98.

Starting material **97** (5 grams, 7.82 mmol) and imidazole (1.33 grams, 19.5 mmol, 2.5 equiv.) was suspended in dry DMF (80 ml) in a 250 ml RBF. TBDMS-Cl was added (1.77 grams, 11.7 mmol, 1.5 equiv.), and the reaction was stirred at room temperature under argon

overnight. Upon completion (TLC, 1:1 EtOAC/hexanes), the rection mixture was diluted with EtOAc (200 ml) and was transferred to a separatory funnel. The reaction was washed with saturated aqueous $NaHCO_3$ (2 x 100 ml) and brine (1 x 200 ml). The organics were collected, dried over $MgSO_4$, filtered, and concentrated to a syrup. The crude material was purified by silica gel chromatography (EtOAC/hexanes). The product was dried under high vacuum to give **98** as brittle foam. Yield: 5.31 grams (90 %). ^{1}H NMR (300MHz, CHLOROFORM-d) δ = 11.92 (s, 1H), 7.80 (s, 1H), 7.69 (s, 1H), 7.52 - 7.44 (m, 2H), 7.39 - 7.28 (m, 5H), 7.26 - 7.18 (m, 2H), 6.85 - 6.77 (m, 4H), 6.20 (dd, *J*=5.4, 8.3 Hz, 1H), 4.61 - 4.55 (m, 1H), 4.09 - 4.02 (m, 1H), 3.78 (d, *J*=2.2 Hz, 6H), 3.38 (dd, *J*=3.5, 10.5 Hz, 1H), 3.15 (dd, *J*=3.9, 10.5 Hz, 1H), 2.90 - 2.76 (m, 1H), 2.30 (ddd, *J*=2.5, 5.4, 12.9 Hz, 1H), 2.01 (spt, *J*=6.9 Hz, 1H), 1.63 (s, 1H), 1.08 (d, *J*=6.9 Hz, 3H), 0.98 - 0.81 (m, 12H), 0.12 - -0.02 (m, 6H). ^{13}C NMR (75MHz, CHLOROFORM-d) δ = 177.93, 158.67, 155.43, 147.99, 146.98, 144.78, 137.57, 135.96, 135.75, 129.96, 128.06, 127.99, 127.05, 122.34, 113.25, 87.15, 86.28, 84.51, 72.53, 63.31, 55.27, 40.63, 36.40, 25.76, 25.66, 18.73, 18.67, 17.98, -4.72, -4.80. LCMS (ESI) m/z: [M-H] calcd, 753.9; found, 752.4.

Compound 99.

98 (5.31 grams, 7.04 mmol) was dissolved in 80% acetic acid (20 ml). The reaction was heated to 40 C ° for 60 min, and was monitored by TLC (100% EtOAc, Rf = 0.5). Upon completion, the reaction was cooled and diluted with EtOAc (150 ml) and was transferred to a separatory funnel. Saturated aqueous $NaHCO_3$ (50 ml) was carefully added. The organics were collected and were washed with $NaHCO_3$ (50 ml) and brine (50 ml). The organics were dried over $MgSO_4$, filtered, and the solvent was removed under reduced pressure. The crude material was purified by silica gel chromatography (10% MeOH in DCM). The desired product was dried under high vacuum to give **99** as pale yellow brittle foam. yield: 1.92 grams (60%). ^{1}H NMR (300MHz, CHLOROFORM-d) δ = 12.22 - 11.85 (m, 1H), 8.51 (br s, 1H), 7.84 - 7.74 (m, 1H), 7.37 - 7.28 (m, 1H), 6.19 (dd, *J*=5.7, 8.8 Hz, 1H), 5.32 - 4.95 (m, 1H), 4.61 (br d, *J*=5.4 Hz, 1H), 4.18 - 4.04 (m, 1H), 3.95 (dd, *J*=1.9, 12.4 Hz, 1H), 3.87 - 3.57 (m, 2H), 2.93 - 2.47 (m, 2H), 2.43 - 1.73 (m, 3H), 1.31 - 1.07 (m, 6H), 0.98 - 0.82 (m, 9H), 0.17 - -0.01 (m, 6H). ^{13}C NMR (75MHz, CHLOROFORM-d) δ = 178.46, 155.15, 147.40, 146.90, 138.46, 122.77, 89.36, 86.71,

73.45, 62.98, 41.44, 36.48, 25.73, 18.90, 17.98, -4.79, -4.87. LCMS (ESI) m/z: [M+H] calcd, 451.6; found, 452.2.

Compound 101.

Oxalyl chloride (480 µl, 5.60 mmol, 1.33 equiv.) was dissolved in dry DCM (40 ml) and the reaction was cooled to -78 °C with stirring under nitrogen. DMSO was added via syringe (780 µl, 11.0 mmol, 2.61 equiv.) and the reaction was allowed to stir for 10 min. In a separate vessel, intermediate **99** (1.9 grams, 4.21 mmol) was dissolved in dry DCM (5 ml) and was added to the reaction via syringe. The reaction was stirred at – 78 °C for 15 minutes. Et_3N (2.31 ml, 16.6 mmol, 3.94 equiv.) was added and the reaction was allowed to warm to room temperature. The reaction was diluted with EtOAc (70 ml) and was transferred to a separatory funnel. The reaction mixture was washed with 1: 1 water and saturated aqueous $NaHCO_3$ (3 x 100 ml) followed by brine (1 x 100 ml). The organics were collected, dried over $MgSO_4$, filtered, and concentrated under reduced pressure (rotovap). The aldehyde intermediate **100** was used immediately without further purification

The crude aldehyde **100** was dissolved in dry DCM (100 ml), and the reaction was cooled in an ince bath with stirring under nitrogen. TMS allylsilane was added via syringe (3.34 ml, 21.0 mmol, 5 equiv.), followed by BF_3-Et_2O (2.64 ml, 2.14 mmol, 5 equiv.). The reaction was stirred at 0 °C for 30 min. The reaction was diluted with EtOAc (50 ml) and was transferred to a separatory funnel. The reaction mixture was washed with 1: 1 water and saturated aqueous $NaHCO_3$ (3 x 100 ml) followed by brine (1 x 100 ml). The organics were collected, dried over $MgSO_4$, filtered, and concentrated under reduced pressure (rotovap). The crude product was purified by silica gel chromatography (DCM/MeOH) to give the desired product **101** as an inseparable mixture of isomers. 1.59 grams (77% as a brittle yellow foam)· ^{1}H NMR (300MHz, CHLOROFORM-d) δ = 12.32 - 12.05 (m, 1H), 9.26 - 9.15 (m, 1H), 7.99 - 7.74 (m, 1H), 6.37 - 6.10 (m, 1H), 5.98 - 5.72 (m, 1H), 5.26 - 5.02 (m, 2H), 4.75 - 4.39 (m, 1H), 4.13 - 3.86 (m, 2H), 3.82 - 3.66 (m, 1H), 2.99 - 2.61 (m, 3H), 2.50 - 2.16 (m, 3H), 1.47 - 1.19 (m, 7H), 1.04 - 0.69 (m, 13H), 0.17 - -0.20 (m, 9H)· ^{13}C NMR (75MHz, CHLOROFORM-d) δ = 179.00, 155.31, 147.55, 147.39, 147.22, 138.94, 134.72, 134.34, 122.45, 117.57, 91.55, 86.19, 74.49, 71.55, 71.48, 71.28,

41.28, 39.11, 37.26, 36.26, 36.16, 25.71, 25.69, 25.64, 18.88, 18.84, 18.77, 17.93, 17.79, -4.53, -4.78 (q, *J*=3.5 Hz, 1C), -4.89. LCMS (ESI) m/z: [M+H] calcd, 491.7; found, 492.2

Compound 102. Precursor **101** (3.26 g, 5.29 mmol) was coevaporated with toluene (3x 20 mL) under reduced pressure at 60°C before being dissolved into dry pyridine (10 mL) and 2,6-lutidine (1.84 mL, 15.9 mmol, 3 eq). To this was added DMT-Cl (7.18 g, 21.20 mmol, 4 eq) and silver nitrate (1.80 g, 10.6 mmol, 2 eq) and the reaction was heated to 45 °C for 16 hours. TLC in EtOAc/hexanes (6/4) indicated reaction was complete. The reaction mixture was cooled in an ice bath and was quenched by addition of ethyl acetate followed by water. The aqueous layer was removed and the organics were washed with saturated sodium bicarbonate solution and brine. The organics were collected, dried over Na_2SO_3, filtered and concentrated to a crude yellow oil under reduced pressure. The crude product was purified by flash chromatography (silica gel, 220g col, 0-50% ethyl acetate/hexanes) to give **102** as yellow foam. Yield: 2.20 g (45 %). ^{1}H NMR (300MHz, CHLOROFORM-d) δ = 11.90 (s, 1H), 7.96 - 7.86 (m, 1H), 7.71 (d, *J*=6.6 Hz, 2H), 7.64 - 7.50 (m, 3H), 7.47 - 7.09 (m, 18H), 6.85 - 6.57 (m, 4H), 3.80 - 3.70 (m, 6H), 1.92 (br s, 2H), 1.66 (br s, 3H), 1.25 (t, *J*=6.8 Hz, 5H), 1.14 - 1.01 (m, 9H), 0.71 (d, *J*=6.9 Hz, 1H), 0.55(d, *J*=6.8 Hz, 1H). ^{13}C NMR (75MHz, CHLOROFORM-d) δ = 177.92, 177.84, 158.55, 158.52, 155.57, 155.35, 148.22, 147.17, 146.60, 145.86, 136.35, 136.19, 136.02, 135.90 (br t, *J*=18.8 Hz, 1C), 135.49, 133.94, 133.75, 133.21, 133.18, 130.50, 130.41, 130.07, 130.04, 129.99, 129.93, 129.83, 129.14, 128.23, 127.92, 127.86, 127.79, 127.66, 126.89, 126.84, 121.63, 117.64, 117.51, 113.18, 113.03, 112.95, 88.62, 87.83, 86.83, 85.84, 85.63, 82.88, 74.87, 74.68, 73.59, 73.15, 55.31, 55.28, 55.24, 39.17, 36.68, 36.00, 35.35, 26.95, 26.88, 19.05, 18.98, 18.95, 18.48, 17.93. LCMS (ESI) m/z: [M-H] calcd, 779.4; found, 778.2

Compound 103. TEA (0.911 mL, 6.53 mmol, 2.5 eq) was added to a solution of **102** (2.40 g, 2.61 mmol) in THF (25 mL). The reaction was cooled in an ice bath under an atmosphere of nitrogen. Triethylamine trihydrofluoride (2.13 mL, 13.1 mmol, 5.0 eq) was added, and the reaction was stirred at room temperature for 16 hours. Triethylamine (1.0 mL) was added and the solvent was removed to give a pale yellow solid. The crude product was purified by flash chromatography (silica gel, 220g col, 0-5% MeOH/DCM) to give compound **103** as a white

solid. Yield: 1.40 g (79 %). ^{1}H NMR (300MHz, CHLOROFORM-d) δ = 8.00 (s, 1H), 7.57 - 7.33 (m, 7H), 7.30 - 7.13 (m, 4H), 6.85 - 6.72 (m, 4H), 6.09 (t, *J*=6.9 Hz, 1H), 5.61 (br d, *J*=6.9 Hz, 1H), 4.93 - 4.76 (m, 2H), 4.76 - 4.61 (m, 1H), 4.05 - 3.96 (m, 1H), 3.90 (s, 1H), 3.80 - 3.70 (m, 6H), 3.58 - 3.47 (m, 1H), 2.90 (br d, *J*=7.3 Hz, 1H), 2.76 - 2.54 (m, 1H), 2.52 - 2.23 (m, 3H), 2.22 - 2.03 (m, 1H), 1.96 (br dd, *J*=6.6, 13.7 Hz, 1H), 1.29 - 1.07 (m, 6H), 0.94 (d, *J*=6.8 Hz, 1H), 0.85 (d, *J*=6.8 Hz, 1H)^{13}C NMR (75MHz, CHLOROFORM-d) δ = 179.19, 178.74, 158.69, 158.67, 155.72, 148.36, 148.32, 147.64, 147.20, 146.53, 146.03, 138.86, 137.18, 136.91, 136.64, 136.58, 136.46, 134.38, 134.31, 130.52, 130.47, 130.26, 129.14, 128.33, 128.10, 127.86, 127.74, 127.01, 122.10, 121.25, 117.57, 117.50, 113.14, 113.06, 87.98, 87.41, 86.83, 86.39, 84.61, 83.14, 73.94, 73.47, 72.00, 71.08, 55.26, 45.89, 39.96, 36.23, 36.12, 35.99, 35.62, 18.96, 18.92, 18.68, 18.38, 9.69. LCMS (ESI) m/z: [M-H] calcd, 665.3; found, 664.2

Compound 104. Intermediate **103** (1.40 g, 2.06 mmol) was dissolved in dry DMF (20 mL) under an atmosphere of nitrogen. To this was added 1H-tetrazole (114 mg, 165 mmol, 0.8 eq) and 1-methylimidazole (41 µL, 0.515 mmol, 0.25 eq), followed by dropwise addition of 2-cyanoethyl-N,N,N',N'-tetraisopropyl-phosphorodiamidite (0.981 mL, 3.10 mmol, 1.5 eq). The reaction was stirred at room temperature for 2 hours, and was monitored by TLC (EtOAc/hexanes, 7:3). Upon completion, the reaction was quenched reaction by addition of water (1 mL). The reaction was dilutied with a mixture of toluene/hexanes (3:1) and was transferred to a separatory funel. The organics were washed four times with a mixture of DMF/H_2O (3:2). The organics were washed with saturated sodium bicarbonate solution and brine. The organic layer was collected, dried over sodium sulfate, and concentrated to a white foam. The crude product was purified by flash chromatography (silica gel, 25 g col, 50-80% EtOAc/Hexane + 1 % triethyl amine) to give **104** as a white solid. Yield: 1.24 g (68 %).^{1}H NMR (300MHz, CHLOROFORM-d) δ = 11.96 (br s, 1H), 7.63 - 7.56 (m, 1H), 7.51 - 7.17 (m, 6H), 6.84 - 6.70 (m, 2H), 6.15 - 5.98 (m, 1H), 5.03 - 4.68 (m, 2H), 4.18 - 4.05 (m, 1H), 3.81 - 3.52 (m, 6H), 2.87 - 2.62 (m, 1H), 2.48 - 2.20 (m, 1H), 2.05 (s, 2H), 1.76 (br s, 1H), 1.29 - 1.11 (m, 10H), 1.01 (dd, *J*=6.8, 15.4 Hz, 1H), 0.93 - 0.80 (m, 1H), 0.69 (d, *J*=6.8 Hz, 1H). ^{31}P NMR (121MHz, CHLOROFORM-d) δ = 149.12 (s, 1P), 148.99 (s, 1P), 148.87 (s, 1P), 147.09 (s, 1P)LCMS (ESI) m/z: [M-H] calcd, 865.4; found, 864.3

Figure S1. Determination of the absolute configuration of 5’-Me group.

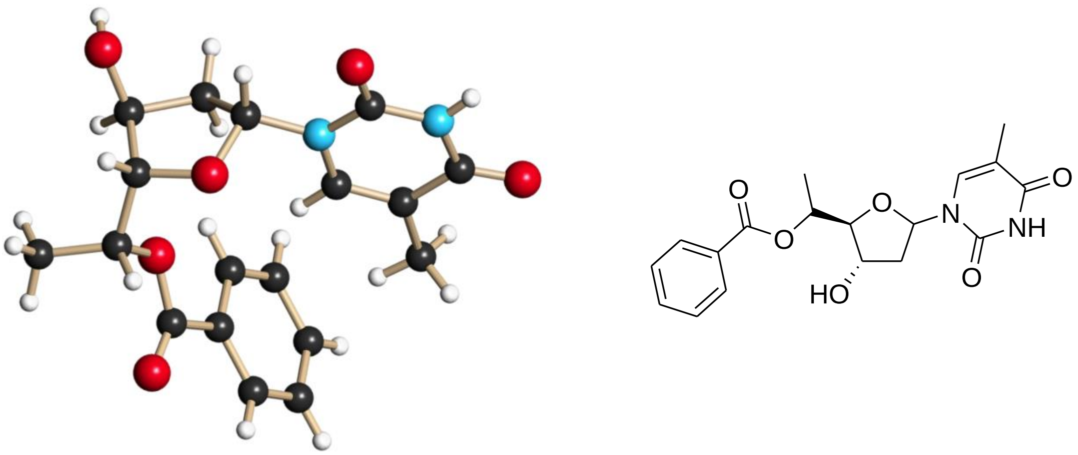

Table S1. Crystal data and structure refinement.

Identification code	isis19	
Empirical formula	C18 H20 N2 O6	
Formula weight	360.36	
Temperature	100(2) K	
Wavelength	0.71073 Å	
Crystal system	Orthorhombic	
Space group	P2(1)2(1)2(1)	
Unit cell dimensions	a = 10.8056(14) Å	a= 90°
	b = 16.240(2) Å	b= 90°
	c = 19.613(3) Å	g = 90°
Volume	3441.6(8) $Å^3$	
Z, Z’	8, 2	
Density (calculated)	1.391 g/cm^3	

Absorption coefficient	0.105 mm^{-1}
F(000)	1520
Crystal size	0.40 x 0.20 x 0.10 mm^3
Theta range for data collection	1.63 to 25.54°
Index ranges	-13<=h<=12, -16<=k<=19, -23<=l<=23
Reflections collected	14565
Independent reflections	6132 [R(int) = 0.0529]
Completeness to theta = 25.00°	98.6 %
Absorption correction	Multi-scan
Refinement method	Full-matrix least-squares on F^2
Data / restraints / parameters	6132 / 0 / 473
Goodness-of-fit on F^2	1.029
Final R indices [I>2sigma(I)]	R1 = 0.0515, wR2 = 0.1041
R indices (all data)	R1 = 0.0792, wR2 = 0.1190
Absolute structure parameter	0.3(11)
Largest diff. peak and hole	0.279 and -0.298 e $Å^{-3}$

Table S2. Atomic coordinates (x 10^4) and equivalent isotropic displacement parameters ($Å^2$x 10^3). U(eq) is defined as one third of the trace of the orthogonalized U^{ij} tensor.

	x	y	z	U(eq)
O(1)	5798(2)	8555(1)	10274(1)	26(1)
O(1')	6197(2)	1448(1)	10340(1)	29(1)
O(2)	5959(2)	7952(1)	9241(1)	19(1)
O(2')	6049(2)	2050(1)	9307(1)	20(1)
O(3)	7069(2)	5866(1)	8062(1)	24(1)
O(3')	4590(2)	4046(1)	8175(1)	31(1)
O(4)	4912(2)	6361(1)	9116(1)	22(1)
O(4')	7016(2)	3645(1)	9151(1)	22(1)
O(5)	-352(2)	7342(2)	8278(1)	30(1)
O(5')	12294(2)	3059(1)	8162(1)	29(1)
O(6)	2451(2)	5251(1)	8315(1)	26(1)
O(6')	9260(2)	4979(1)	8318(1)	26(1)
N(1)	3176(2)	6562(2)	8418(1)	19(1)
N(1')	8681(2)	3612(2)	8388(2)	20(1)
N(2)	1081(2)	6319(2)	8257(1)	22(1)
N(2')	10729(2)	3988(2)	8210(1)	20(1)
C(1)	4936(3)	9300(2)	8596(2)	18(1)
C(1')	7141(3)	707(2)	8677(2)	22(1)
C(2)	4290(3)	9931(2)	8278(2)	22(1)
C(2')	7762(3)	56(2)	8371(2)	25(1)
C(3)	3611(3)	10484(2)	8674(2)	24(1)
C(3')	8381(3)	-513(2)	8777(2)	28(1)

C(4)	3590(3)	10414(2)	9377(2)	28(1)
C(4')	8388(3)	-432(2)	9480(2)	27(1)
C(5)	4253(3)	9788(2)	9697(2)	25(1)
C(5')	7741(3)	211(2)	9786(2)	24(1)
C(6)	4932(3)	9230(2)	9299(2)	19(1)
C(6')	7108(3)	778(2)	9378(2)	19(1)
C(7)	5616(3)	8563(2)	9669(2)	19(1)
C(7')	6397(3)	1438(2)	9737(2)	19(1)
C(8)	6513(3)	7225(2)	9568(2)	20(1)
C(8')	5472(3)	2763(2)	9636(2)	22(1)
C(9)	7883(3)	7349(2)	9690(2)	25(1)
C(9')	4106(3)	2616(2)	9760(2)	28(1)
C(10)	6242(3)	6509(2)	9096(2)	20(1)
C(10')	5699(3)	3480(2)	9161(2)	21(1)
C(11)	6592(3)	6620(2)	8340(2)	19(1)
C(11')	5287(3)	3357(2)	8416(2)	20(1)
C(12)	5352(3)	6785(2)	7994(2)	19(1)
C(12')	6500(3)	3310(2)	8024(2)	20(1)
C(13)	4457(3)	6279(2)	8431(2)	20(1)
C(13')	7374(3)	3813(2)	8461(2)	20(1)
C(14)	2904(3)	7389(2)	8524(2)	21(1)
C(14')	9074(3)	2793(2)	8427(2)	21(1)
C(15)	1753(3)	7694(2)	8484(2)	22(1)
C(15')	10250(3)	2557(2)	8347(2)	20(1)
C(16)	1471(3)	8597(2)	8598(2)	30(1)
C(16')	10645(3)	1671(2)	8392(2)	29(1)
C(17)	734(3)	7139(2)	8336(2)	23(1)
C(17')	11186(3)	3187(2)	8234(2)	23(1)
C(18)	2240(3)	5997(2)	8334(2)	21(1)
C(18')	9539(3)	4237(2)	8311(2)	22(1)

Table S3. Bond lengths [Å] and angles [°].

O(1)-C(7)	1.201(4)	C(1)-C(2)	1.387(4)
O(1')-C(7')	1.203(4)	C(1')-C(6')	1.381(4)
O(2)-C(7)	1.352(4)	C(1')-C(2')	1.388(5)
O(2)-C(8)	1.471(4)	C(2)-C(3)	1.397(5)
O(2')-C(7')	1.357(4)	C(2')-C(3')	1.391(5)
O(2')-C(8')	1.465(4)	C(3)-C(4)	1.384(5)
O(3)-C(11)	1.436(4)	C(3')-C(4')	1.385(5)
O(3')-C(11')	1.430(4)	C(4)-C(5)	1.393(5)
O(4)-C(13)	1.437(4)	C(4')-C(5')	1.393(5)
O(4)-C(10)	1.458(4)	C(5)-C(6)	1.402(5)
O(4')-C(13')	1.433(4)	C(5')-C(6')	1.397(4)
O(4')-C(10')	1.449(4)	C(6)-C(7)	1.500(5)
O(5)-C(17)	1.224(4)	C(6')-C(7')	1.495(5)
O(5')-C(17')	1.223(4)	C(8)-C(9)	1.513(4)
O(6)-C(18)	1.234(4)	C(8)-C(10)	1.515(4)
O(6')-C(18')	1.241(4)	C(8')-C(10')	1.510(4)
N(1)-C(18)	1.376(4)	C(8')-C(9')	1.516(5)
N(1)-C(14)	1.389(4)	C(10)-C(11)	1.540(5)
N(1)-C(13)	1.459(4)	C(10')-C(11')	1.541(5)
N(1')-C(18')	1.383(4)	C(11)-C(12)	1.525(4)
N(1')-C(14')	1.398(4)	C(11')-C(12')	1.522(4)
N(1')-C(13')	1.457(4)	C(12)-C(13)	1.530(4)
N(2)-C(18)	1.366(4)	C(12')-C(13')	1.515(4)
N(2)-C(17)	1.392(4)	C(14)-C(15)	1.342(5)
N(2')-C(18')	1.362(4)	C(14')-C(15')	1.336(4)
N(2')-C(17')	1.393(4)	C(15)-C(17)	1.453(5)
C(1)-C(6)	1.385(4)	C(15)-C(16)	1.514(5)

C(15')-C(17')	1.456(4)	C(15')-C(16')	1.503(4)

C(7)-O(2)-C(8)	115.4(2)	O(1)-C(7)-O(2)	124.1(3)
C(7')-O(2')-C(8')	115.1(3)	O(1)-C(7)-C(6)	124.5(3)
C(13)-O(4)-C(10)	109.0(2)	O(2)-C(7)-C(6)	111.4(3)
C(13')-O(4')-C(10')	108.2(2)	O(1')-C(7')-O(2')	123.5(3)
C(18)-N(1)-C(14)	120.5(3)	O(1')-C(7')-C(6')	124.4(3)
C(18)-N(1)-C(13)	119.3(3)	O(2')-C(7')-C(6')	112.1(3)
C(14)-N(1)-C(13)	120.2(3)	O(2)-C(8)-C(9)	111.2(3)
C(18')-N(1')-C(14')	120.0(3)	O(2)-C(8)-C(10)	105.7(2)
C(18')-N(1')-C(13')	119.8(3)	C(9)-C(8)-C(10)	112.8(3)
C(14')-N(1')-C(13')	120.1(3)	O(2')-C(8')-C(10')	105.6(3)
C(18)-N(2)-C(17)	126.9(3)	O(2')-C(8')-C(9')	111.1(3)
C(18')-N(2')-C(17')	127.4(3)	C(10')-C(8')-C(9')	112.3(3)
C(6)-C(1)-C(2)	120.5(3)	O(4)-C(10)-C(8)	107.5(3)
C(6')-C(1')-C(2')	120.4(3)	O(4)-C(10)-C(11)	106.8(2)
C(1)-C(2)-C(3)	119.3(3)	C(8)-C(10)-C(11)	116.9(3)
C(1')-C(2')-C(3')	119.4(3)	O(4')-C(10')-C(8')	108.1(3)
C(4)-C(3)-C(2)	120.7(3)	O(4')-C(10')-C(11')	107.1(3)
C(4')-C(3')-C(2')	120.7(3)	C(8')-C(10')-C(11')	116.0(3)
C(3)-C(4)-C(5)	120.0(3)	O(3)-C(11)-C(12)	107.3(3)
C(3')-C(4')-C(5')	119.8(3)	O(3)-C(11)-C(10)	110.7(3)
C(4)-C(5)-C(6)	119.4(3)	C(12)-C(11)-C(10)	103.4(3)
C(4')-C(5')-C(6')	119.5(3)	O(3')-C(11')-C(12')	109.0(3)
C(1)-C(6)-C(5)	120.2(3)	O(3')-C(11')-C(10')	111.4(3)
C(1)-C(6)-C(7)	122.7(3)	C(12')-C(11')-C(10')	103.7(3)
C(5)-C(6)-C(7)	117.1(3)	C(11)-C(12)-C(13)	102.3(3)
C(1')-C(6')-C(5')	120.2(3)	C(13')-C(12')-C(11')	102.9(3)
C(1')-C(6')-C(7')	122.8(3)	O(4)-C(13)-N(1)	108.1(3)
C(5')-C(6')-C(7')	117.0(3)	O(4)-C(13)-C(12)	105.0(3)

N(1)-C(13)-C(12)	114.9(3)
O(4')-C(13')-N(1')	108.2(3)
O(4')-C(13')-C(12')	105.3(3)
N(1')-C(13')-C(12')	115.3(3)
C(15)-C(14)-N(1)	123.0(3)
C(15')-C(14')-N(1')	123.7(3)
C(14)-C(15)-C(17)	119.0(3)
C(14)-C(15)-C(16)	122.4(3)
C(17)-C(15)-C(16)	118.6(3)
C(14')-C(15')-C(17')	118.5(3)
C(14')-C(15')-C(16')	122.6(3)
C(17')-C(15')-C(16')	118.9(3)
O(5)-C(17)-N(2)	120.4(3)
O(5)-C(17)-C(15)	125.3(3)
N(2)-C(17)-C(15)	114.3(3)
O(5')-C(17')-N(2')	120.1(3)
O(5')-C(17')-C(15')	125.4(3)
N(2')-C(17')-C(15')	114.5(3)
O(6)-C(18)-N(2)	122.8(3)
O(6)-C(18)-N(1)	121.6(3)
N(2)-C(18)-N(1)	115.6(3)
O(6')-C(18')-N(2')	121.3(3)
O(6')-C(18')-N(1')	123.2(3)
N(2')-C(18')-N(1')	115.5(3)

Table S4. Anisotropic displacement parameters ($Å^2$x 10^3). The anisotropic displacement factor exponent takes the form: $-2p^2[h^2 a^{*2}U^{11} + ... + 2 h k a^* b^* U^{12}]$

	U^{11}	U^{22}	U^{33}	U^{23}	U^{13}	U^{12}
O(1)	26(1)	26(1)	27(2)	1(1)	-3(1)	1(1)
O(1')	33(2)	30(2)	24(2)	3(1)	3(1)	2(1)
O(2)	20(1)	15(1)	23(1)	0(1)	-1(1)	3(1)
O(2')	21(1)	17(1)	23(1)	-1(1)	1(1)	3(1)
O(3)	24(1)	21(1)	29(1)	-5(1)	6(1)	3(1)
O(3')	16(1)	31(1)	46(2)	10(1)	-5(1)	5(1)
O(4)	15(1)	26(1)	25(1)	4(1)	0(1)	-3(1)
O(4')	15(1)	27(1)	23(1)	-3(1)	-1(1)	-4(1)
O(5)	15(1)	44(2)	30(1)	3(1)	-3(1)	6(1)
O(5')	13(1)	35(2)	40(2)	-7(1)	1(1)	3(1)
O(6)	17(1)	22(1)	38(2)	-3(1)	0(1)	-6(1)
O(6')	20(1)	18(1)	41(2)	1(1)	-3(1)	-5(1)
N(1)	11(1)	17(2)	29(2)	-1(1)	-3(1)	-2(1)
N(1')	10(1)	13(1)	35(2)	2(1)	0(1)	1(1)
N(2)	12(2)	28(2)	25(2)	-4(1)	-3(1)	-4(1)
N(2')	13(2)	21(2)	27(2)	4(1)	2(1)	-1(1)
C(1)	14(2)	14(2)	26(2)	-1(1)	-2(1)	-3(2)
C(1')	19(2)	16(2)	29(2)	2(2)	-1(2)	-2(2)
C(2)	20(2)	19(2)	27(2)	3(2)	-1(2)	-5(2)
C(2')	23(2)	23(2)	30(2)	1(2)	3(2)	3(2)
C(3)	17(2)	18(2)	38(2)	3(2)	-2(2)	1(2)
C(3')	18(2)	19(2)	46(3)	-3(2)	2(2)	1(2)
C(4)	19(2)	17(2)	48(3)	-5(2)	2(2)	1(2)

C(4')	18(2)	24(2)	39(2)	7(2)	-6(2)	1(2)
C(5)	18(2)	28(2)	28(2)	-4(2)	2(2)	-8(2)
C(5')	22(2)	24(2)	26(2)	4(2)	0(2)	-4(2)
C(6)	13(2)	16(2)	28(2)	0(2)	-2(1)	-2(1)
C(6')	14(2)	14(2)	29(2)	-1(2)	0(2)	-4(2)
C(7)	15(2)	20(2)	23(2)	0(2)	2(2)	-5(2)
C(7')	17(2)	15(2)	25(2)	4(2)	-2(2)	-8(2)
C(8)	21(2)	16(2)	24(2)	6(2)	-3(2)	1(2)
C(8')	18(2)	19(2)	28(2)	-4(2)	4(2)	0(2)
C(9)	17(2)	22(2)	34(2)	-3(2)	-3(2)	3(2)
C(9')	23(2)	25(2)	36(2)	-1(2)	8(2)	-1(2)
C(10)	13(2)	18(2)	29(2)	5(2)	-4(2)	1(2)
C(10')	10(2)	20(2)	32(2)	2(2)	5(2)	-3(2)
C(11)	15(2)	14(2)	28(2)	-1(2)	4(2)	-1(1)
C(11')	12(2)	17(2)	30(2)	1(2)	1(2)	2(1)
C(12)	18(2)	16(2)	24(2)	0(1)	3(2)	0(2)
C(12')	10(2)	20(2)	31(2)	2(2)	-2(2)	-2(2)
C(13)	13(2)	20(2)	26(2)	-2(2)	-3(2)	2(2)
C(13')	15(2)	18(2)	28(2)	2(2)	2(2)	1(2)
C(14)	18(2)	20(2)	26(2)	1(1)	2(1)	0(2)
C(14')	19(2)	19(2)	25(2)	-1(2)	0(1)	-3(2)
C(15)	18(2)	23(2)	24(2)	3(2)	3(1)	1(2)
C(15')	15(2)	22(2)	24(2)	-2(2)	-1(2)	4(2)
C(16)	20(2)	29(2)	39(2)	3(2)	1(2)	6(2)
C(16')	22(2)	24(2)	42(2)	-3(2)	-4(2)	3(2)
C(17)	19(2)	28(2)	22(2)	4(2)	1(2)	3(2)
C(17')	17(2)	28(2)	22(2)	-3(2)	-1(2)	-1(2)
C(18)	14(2)	28(2)	21(2)	2(2)	-1(2)	-2(2)
C(18')	15(2)	27(2)	23(2)	-3(2)	-2(2)	- 2(2)

Table S5. Hydrogen coordinates (x 10^4) and isotropic displacement parameters ($Å^2$ x 10^3).

	x	y	z	U(eq)
H(3B)	7255	5936	7650	37
H(3'B)	3846	3909	8127	47
H(2B)	490	5971	8145	26
H(2'B)	11270	4378	8119	24
H(1A)	5383	8913	8329	22
H(1'A)	6737	1106	8401	26
H(2A)	4309	9985	7795	26
H(2'A)	7764	-1	7889	30
H(3A)	3158	10913	8459	29
H(3'A)	8802	-961	8571	33
H(4B)	3124	10793	9642	34
H(4'A)	8833	-814	9753	32
H(5A)	4246	9739	10179	30
H(5'A)	7729	264	10268	29
H(8A)	6093	7127	10015	24
H(8'A)	5893	2871	10081	26
H(9A)	8006	7815	10000	37
H(9B)	8297	7461	9255	37
H(9C)	8234	6850	9894	37
H(9'A)	4003	2152	10075	42
H(9'B)	3696	2489	9327	42
H(9'C)	3734	3111	9959	42
H(10A)	6675	6010	9274	24
H(10B)	5265	3974	9348	25
H(11A)	7182	7087	8274	23

H(11B)	4804	2835	8364	23
H(12A)	5354	6592	7515	23
H(12B)	5140	7378	8006	23
H(12C)	6788	2733	7981	24
H(12D)	6414	3552	7563	24
H(13A)	4494	5688	8289	24
H(13B)	7248	4412	8363	24
H(14A)	3564	7754	8630	26
H(14B)	8470	2381	8515	25
H(16A)	2233	8886	8725	44
H(16B)	861	8653	8965	44
H(16C)	1137	8836	8178	44
H(16D)	9915	1322	8458	44
H(16E)	11211	1602	8779	44
H(16F)	11067	1513	7970	44

Supplementary Table S6 – Sequence, design and analytical data on synthesized ASOs, blue letters represent cEt, black DNA and red represent modifications.

Ion #	Sequence (5′ to 3′)	Modification	UV purity	Mass calcd	Mass found
558807	GCATGTTCTCACATTA	DNA	96.0	5380.5	5379.6
942943	GCATGTTCTCACATTA	*R*-5′-Me DNA	94.3	5394.5	5392.8
942944	GCATGTTCTCACATTA	*R*-5′-Me DNA	93.8	5394.5	5392.9
957908	GCATGTTCTCACATTA	*S*-5′-Me DNA	96.4	5394.5	5393.7
957909	GCATGTTCTCACATTA	*S*-5′-Me DNA	98.5	5394.5	5394.1
957910	GCATGTTCTCACATTA	*R*-5′-allyl DNA	93.5	5420.6	5419.6
957911	GCATGTTCTCACATTA	*R*-5′-allyl DNA	96.1	5420.6	5419.6
957912	GCATGTTCTCACATTA	*S*-5′-allyl DNA	93.1	5420.6	5420.0
957913	GCATGTTCTCACATTA	*S*-5′-allyl DNA	95.3	5420.6	5419.9
989027	GCATGTTCTCACATTA	bcDNA	96.8	5406.6	5405.9
989028	GCATGTTCTCACATTA	bcDNA	96.1	5406.6	5405.9
1318996	GCATGTTCTCACATTA	*S*-5′-Me DNA	83.7	5394.5	5394.2
1123318	GCATGTTCTCACATTA	*S*-5′-Me DNA	93.0	5394.5	5394.3
957908	GCATGTTCTCACATTA	*S*-5′-Me DNA	96.4	5394.5	5393.67
957909	GCATGTTCTCACATTA	*S*-5′-Me DNA	98.5	5394.5	5394.1
1318997	GCATGTTCTCACATTA	*S*-5′-Me DNA	91.9	5394.5	5394.4
1318998	GCATGTTCTCACATTA	*S*-5′-Me DNA	85.8	5394.5	5394.3
1318999	GCATGTTCTCACATTA	*S*-5′-Me DNA	92.7	5394.5	5394.3
1319000	GCATGTTCTCACATTA	*S*-5′-Me DNA	95.5	5394.5	5394.3
1319001	GCATGTTCTCACATTA	*S*-5′-Me DNA	92.0	5394.5	5394.3
1319002	GCATGTTCTCACATTA	*S*-5′-Me DNA	85.1	5394.5	5394.2
1319011	GCATGTTCTCACATTA	2'-OMe; *S*-5′-Me DNA	91.6	5424.6	5424.4
1319003	GCATGTTCTCACATTA	*R*-5′-Me DNA	83.1	5394.5	5394.3
1123322	GCATGTTCTCACATTA	*R*-5′-Me DNA	95.6	5394.5	5394.4
942943	GCATGTTCTCACATTA	*R*-5′-Me DNA	94.3	5394.5	5392.8
942944	GCATGTTCTCACATTA	*R*-5′-Me DNA	93.8	5394.5	5392.9
1319004	GCATGTTCTCACATTA	*R*-5′-Me DNA	85.6	5394.5	5394.3
1319005	GCATGTTCTCACATTA	*R*-5′-Me DNA	84.0	5394.5	5394.4
1319006	GCATGTTCTCACATTA	*R*-5′-Me DNA	85.9	5394.5	5394.4
1319007	GCATGTTCTCACATTA	*R*-5′-Me DNA	95.6	5394.5	5394.1
1319008	GCATGTTCTCACATTA	*R*-5′-Me DNA	86.2	5394.5	5394.3
1319009	GCATGTTCTCACATTA	*R*-5′-Me DNA	92.4	5394.5	5394.3
1319010	GCATGTTCTCACATTA	2'-OMe; *R*-5′-Me DNA	86.0	5424.6	5424.3
1123320	GCATGTTCTCACATTA	*R*-5′-Me DNA	96.0	5394.5	5394.5
1123322	GCATGTTCTCACATTA	*S*-5′-Me DNA	95.6	5394.5	5394.4
1123479	GCATGTTCTCACATTA	5′-Allyl DNA	94.0	5420.6	5420.6
1175782	GCATGTTCTCACATTA	*R*-5′-Et DNA	93.1	5408.5	5408.2
1175785	GCATGTTCTCACATTA	*R*-5′-Et DNA	94.7	5408.5	5408.3
1175786	GCATGTTCTCACATTA	*S*-5′-Et DNA	94.4	5408.5	5408.4

1175787	GCATGTTCTCACATTA	*S*-5’-Et DNA	93.6	5408.5	5408.4
Ion #	Sequence (5’ to 3’)	Modification	UV purity	Mass calcd	Mass found
1133122	TAGTCTCTGTCAGTTA	OMe	94.3	5413.5	5412.6
1280765	TAGTCxTCTGTCAGTTA	MOP	92.9	5437.5	5436.6
1280766	TAGTCTCTGTCAGTTA	*R*-5’-Me DNA	94.3	5411.5	5411.3
1280767	TAGTCTCTGTCAGTTA	*R*-5’-Me DNA	83.9	5411.5	5411.6
1280768	TAGTCTCTGTCAGTTA	*R*-5’-Et DNA	86.4	5425.5	5425.7
1280769	TAGTCTCTGTCAGTTA	*R*-5’-Et DNA	86.7	5425.5	5425.6
1133201	TCATGTACCTATAGTC	OMe	95.7	5410.5	5410.2
1280770	TCATGxTACCTATAGTC	MOP	93.6	5420.5	5419.5
1280779	TCATGTACCTATAGTC	*R*-5’-Me DNA	93.6	5394.5	5394.2
1280789	TCATGTACCTATAGTC	*R*-5’-Me DNA	92.9	5394.5	5394.2
1280798	TCATGTACCTATAGTC	*R*-5’-Et DNA	86.3	5408.5	5409.1
1280804	TCATGTACCTATAGTC	5’-Et DNA	84.8	5408.5	5409.2
1133332	GTCAGTATCCCAGTGT	OMe	92.6	5451.5	5450.6
1280775	GTCAGxTATCCCAGTGT	MOP	93.7	5461.5	5460.5
1280784	GTCAGTATCCCAGTGT	*R*-5’-Me DNA	83.8	5435.5	5435.8
1280794	GTCAGTATCCCAGTGT	*R*-5’-Me DNA	90.6	5435.5	5435.5
1280803	GTCAGTATCCCAGTGT	*R*-5’-Et DNA	83.5	5449.6	5449.9
1281809	GTCAGTATCCCAGTGT	5’-Et DNA	84.6	5449.6	5449.8
1133247	GTTATTGTGGTTGGCG	OMe	97.4	5497.5	5496.7
1280774	GTTATxTGTGGTTGGCG	MOP	96.6	5521.5	5520.8
1280783	GTTATTGTGGTTGGCG	*R*-5’-Me DNA	92.8	5495.5	5495.9
1280793	GTTATTGTGGTTGGCG	*R*-5’-Me DNA	90.5	5495.5	5495.8
NT	GTTATTGTGGTTGGCG	*R*-5’-Et DNA	--	--	--
NT	GTTATTGTGGTTGGCG	*R*-5’-Et DNA	---	--	--
1202810	AGACTCTCGGTTCCGA	OMe	94.3	5436.5	5435.6
1280776	AGACTxCTCGGTTCCGA	MOP	96.8	5460.6	5459.8
1280785	AGACTCTCGGTTCCGA	*R*-5’-Me DNA	90.5	5434.5	5434.6
1280795	AGACTCTCGGTTCCGA	*R*-5’-Me DNA	94.9	5434.6	5434.5
1280804	AGACTCTCGGTTCCGA	*R*-5’-Et DNA	93.1	5448.8	5448.6
1280810	AGACTCTCGGTTCCGA	*R*-5’-Et DNA	92.1	5448.8	5448.7

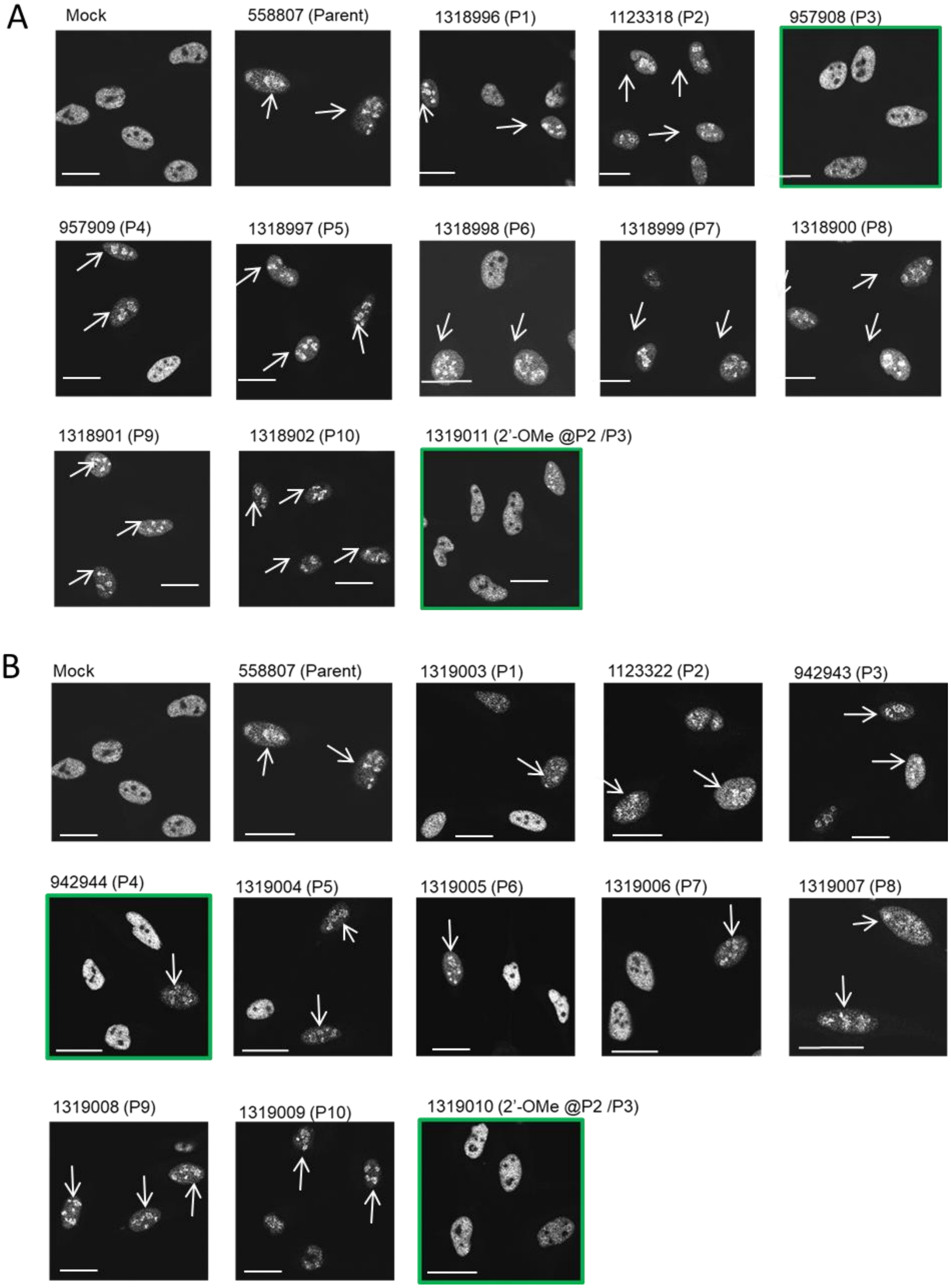

Supplementary Figure S2. Immunofluorescent staining of P54nrb in HeLa cells transected with 200 nM of (A) S-5'Me DNA and (B) R-5'-Me DNA ASOs for 2 h. Mis-localized P54nrb is exemplified with arrows. Scale bar: 20 µm.

A

Ion #		Gap modification	Caspase (% Mock)	ALT @150mg/kg	(%) P54nrb mislocalization
**558807**	GCATGTTCTCACATTA	Parent	2096	Dead	95
957910	GCATGTTCTCACATTA	R-5'-allyl DNA	96	32	11
957911	GCATGTTCTCACATTA	R-5'-allyl DNA	92	30	6
957912	GCATGTTCTCACATTA	S-5'-allyl DNA	112	30	14
957913	GCATGTTCTCACATTA	S-5'-allyl DNA	111	32	5
**1123479**	GCATGTTCTCACATTA	5'-Allyl DNA	302	2561	25
936053	GCATGTTCTCACATTA	OMe	NT	46*	8*

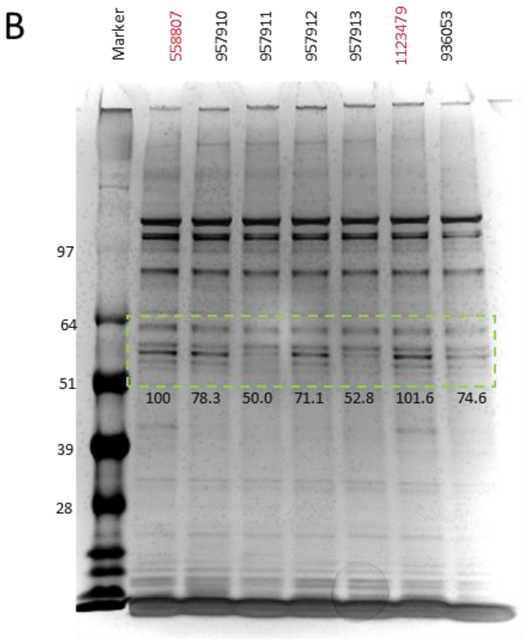

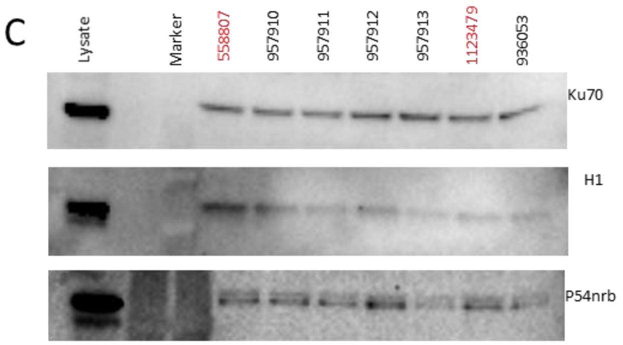

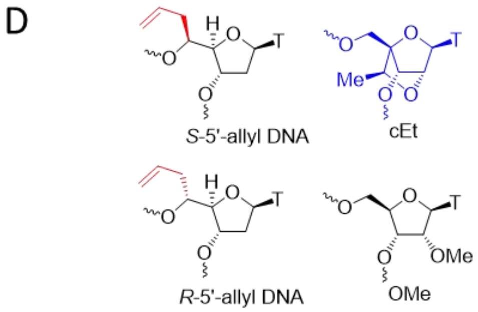

Supplementary Figure S3. 5'-allyl DNA modification that reduce toxicity also reduces protein binding and nucleolar mislocalization. A) 5'-allyl DNA modification can reduce toxicity and protein mislocalization. ASO toxicity is illustrated by increased caspase activity and ALT elevation. P54nrb mislocalization was detected by immunofluorescence staining. The percentage of cells containing mis-localized P54nrb protein was calculated based on manual counting of approximately 100 cells in each condition. B) Silver staining of proteins co-isolated with ASOs of different modifications. The toxic ASOs are marked in red. The proteins in green dotted box were quantified and the levels relative to parental ASO (558807) were calculated and presented below the green box. C) western analyses for the levels of RNase H1 and P54nrb co-isolated with ASOs, as described in panel B. Ku70 protein was probed as a control for loading as the binding of Ku70 is less affected by 2'-modifications. Data indicated by asterisk were obtained from a different experiment. (D) Structures of modifications used in the ASOs.

A

Ion #		Gap modification	Caspase (% Mock)	ALT @150mg/kg	(%) P54 mislocalization
**558807**	GCATGTTCTCACATTA	Parent	2096	Dead	95
957910	GCATGTTCTCACATTA	R-5'-allyl DNA	96	32	11
957911	GCATGTTCTCACATTA	R-5'-allyl DNA	92	30	6
957912	GCATGTTCTCACATTA	S-5'-allyl DNA	112	30	14
957913	GCATGTTCTCACATTA	S-5'-allyl DNA	111	32	5
**1123479**	GCATGTTCTCACATTA	5'-Allyl DNA	302	2561	25
**989027**	GCATGTTCTCACATTA	bicycloDNA	1488	14723	96
**989028**	GCATGTTCTCACATTA	bicycloDNA	399	1397	33

B

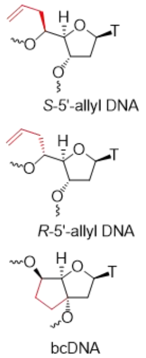

C

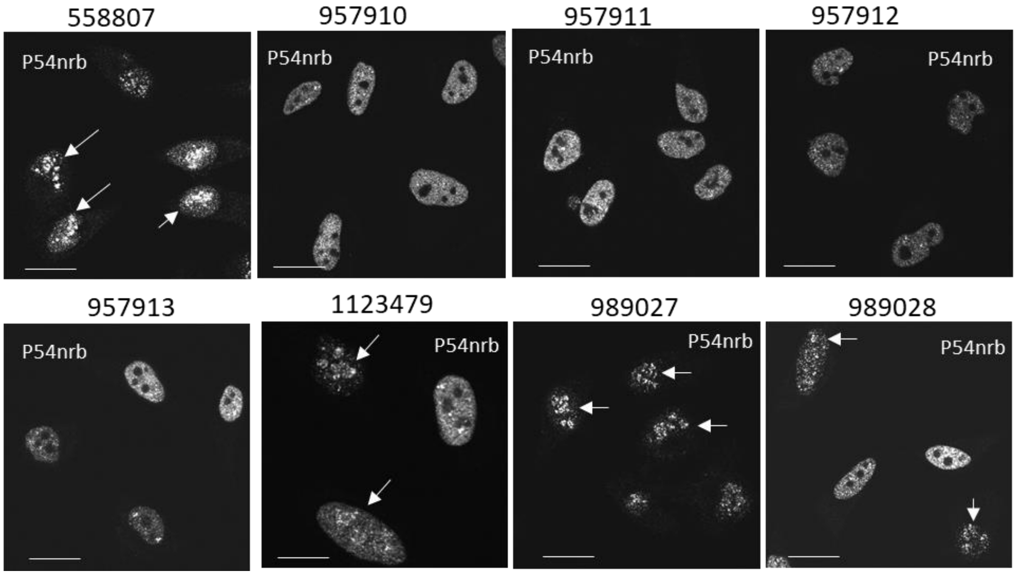

Supplementary Figure S4. (A) 5' allyl modification but not bcDNA can reduce toxicity and protein mislocalization. ASO toxicity is illustrated by increased caspase activity and ALT elevation. (B) Structures of 5'-allyl DNA and bicycloDNA (bcDNA). (C) P54nrb mislocalization was detected by immunofluorescence staining. The percentage of cells containing mis-localized P54nrb protein was calculated based on manual counting of approximately 100 cells in each condition. Immunofluorescent staining of P54nrb in HeLa cells transected with 200 nM of *R*- and *S*-5'-allyl DNA, and bicycloDNA modified ASOs for 2 h. Mis-localized P54nrb is exemplified with arrows. Scale bar: 20 µm.
